# The first theropod dinosaur (Coelurosauria, Theropoda) from the base of the Romualdo Formation (Albian), Araripe Basin, Northeast Brazil

**DOI:** 10.1038/s41598-020-67822-9

**Published:** 2020-07-10

**Authors:** Juliana Manso Sayão, Antônio Álamo Feitosa Saraiva, Arthur Souza Brum, Renan Alfredo Machado Bantim, Rafael Cesar Lima Pedroso de Andrade, Xin Cheng, Flaviana Jorge de Lima, Helder de Paula Silva, Alexander W. A. Kellner

**Affiliations:** 1 Laboratório de Paleobiologia e Microestruturas, Centro Acadêmico de Vitória, Universidade Federal de Pernambuco, Rua Alto do Reservatório, Bela Vista, Vitória de Santo Antão, Pernambuco 55608-680 Brazil; 2 Laboratório de Paleontologia da URCA, Universidade Regional do Cariri, Rua Carolino Sucupira, s/n, Crato, CE 63100-000 Brazil; 3 Laboratory of Systematics and Taphonomy of Fossil Vertebrates, Departamento de Geologia e Paleontologia, Museu Nacional/Universidade Federal do Rio de Janeiro, Quinta da Boa Vista s/n, São Cristóvão, Rio de Janeiro 20940-040 Brazil; 4 Programa de Pós-Graduação em Zoologia, Museu Nacional/Universidade Federal do Rio de Janeiro, Quinta da Boa Vista, São Cristóvão, Rio de Janeiro, RJ 20940-040 Brazil; 5 College of Earth Sciences, Jilin University, Str. Jianshe 2199, Chaoyang distinct, Changchun, 130061 Jilin Province China

**Keywords:** Palaeontology, Evolution

## Abstract

The Romualdo Formation (Araripe Basin) is worldwide known for the large number of well-preserved fossils but the dinosaur record is rather scarce. Here we describe a new coelurosaur, which is the first tetrapod recovered from the basal layers of this stratigraphic unit that consist of dark shales. *Aratasaurus museunacionali* gen. et sp. nov. is known by an incomplete but articulated right hind limb with the distal portion of the femur, proximal half of tibia and incomplete pes. The new species differs from other coelurosaurs by a medial fossa in the tibia and digits II, III and IV being symmetric. The phylogenetic analysis recovered *Aratasaurus museunacionali* closely related to *Zuolong salleei*, forming a basal coelurosaur lineage. The paleohistology indicate that the specimen is a juvenile, with an estimated body length around 3.12 m. The new taxon represents the first occurrence of basal coelurosaurians in the Araripe Basin and suggests a widespread distribution of this group during the Lower Cretaceous.

## Introduction

The dinosaur record in Brazil is still quite meager compared to the potential of the country^1,2^. So far, most specimens were recovered from the Bauru Group, including non-avian theropods^3,4,5,6,7^. The latter are less numerous in these deposits than other reptiles^8^, leading to several discussions, including niche partitioning^7^.

The limited amount of theropod material is not exclusive of Brazil^9^. Among the regions where such reptiles are found in the country is the Araripe Basin^10^. This tectonic structure is worldwide known for the well preserved and diverse fossil biota^11,12,13,14^. The most fossiliferous units are the Lower Cretaceous Crato and Romualdo formations, comprising the majority of fossil vertebrates of this basin^14^. As has been reported several times, the most common tetrapod in both units are pterosaurs^15^, while others tend to be rare^16,17,18^^.^

Until now all non-avian dinosaur from the Araripe Basin came exclusively from the Romualdo Formation and are represented by the spinosaurids *Irritator challengeri*^19^ and *Angaturama limai*^1^ and the non-avian coelurosaurs *Santanaraptor placidus*^20,21^ and *Mirischia assymetrica*^22^. All were preserved in the level of carbonate concretions from the upper portion of this stratigraphic unit^14,23,24^.

Here we describe a new theropod dinosaur from the Romualdo Formation that was collected in the lower section. The fossil is preserved in a slab of dark shale, housed at the Museu de Paleontologia Plácido Cidade Nuvens (MPPCN) of the Universidade Regional do Cariri (URCA), located in the municipality of Santana do Cariri, Ceará State under the number MPSC R 2089. It consists of an incomplete right hind limb, composed of partial femur, tibia and pes. The specimen was on loan to the Museu Nacional/UFRJ for preparation purposes and luckily not affected by the big fire of September 2nd of 2018^25^. The discovery of this new species increases the dinosaur distribution in the several lithological facies of this stratigraphic unit, that is more diverse than previously thought as pointed before^26^.

## Results

### Geological setting

The Mesozoic sedimentary succession of the Araripe Basin encompasses numerous different units, resulting in the proposition of several, sometimes opposing, lithostratigraphic schemes^27,28,29,30,31,32^. Nowadays, there is a consensus to consider the former Santana Formation^12,27^ as the Santana Group, that is further divided from base to top, into the Barbalha, Crato, Ipubi and Romualdo formations^29,31,33^.

**Figure 1:**
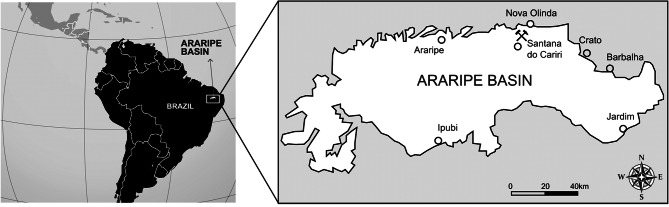
Location map of the Mina Pedra Branca, Ceará State. The crossed geologic hammers indicate where *Aratasaurus museunacionali* gen. et sp. nov. was found. Figure created by Renan Alfredo Machado Bantim on PS Adobe Photoshop CC, version 20.0.6.

**Figure 2:**
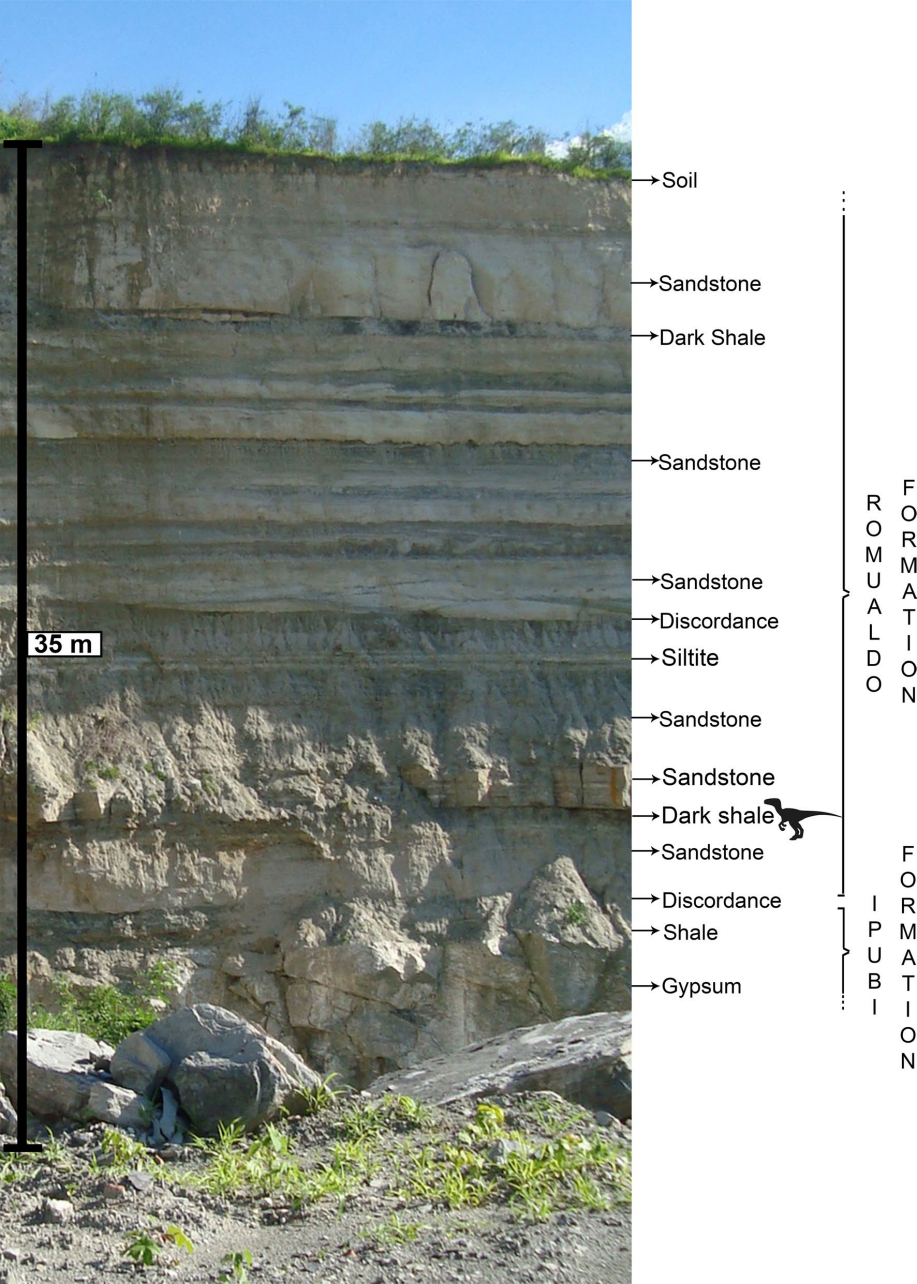
Outcrop of Mina Pedra Branca where *Aratasaurus museunacionali* gen. et sp. nov. was recovered with indication of the stratigraphy and where the dinosaur came from. Figure created by Renan Alfredo Machado Bantim on PS Adobe Photoshop CC, version 20.0.6.

The fossil material studied here is preserved in a dark shale slab with originally 120 cm by 80 cm, and a thickness of around 3 cm. The material was donated to the Museu de Paleontologia Plácido Cidade Nuvens by a local resident of Santana do Cariri who informed that it came from the Mina Pedra Branca. This mine is situated about 5.2 km from the village Santana do Cariri and has been the one of the major sources of fossils from this region^12,34^ (Fig. 1). Comparisons with the shale encompassing the fossil, and the layers of this mine are consistent with this assignment (Fig. 2).

**Figure 3:**
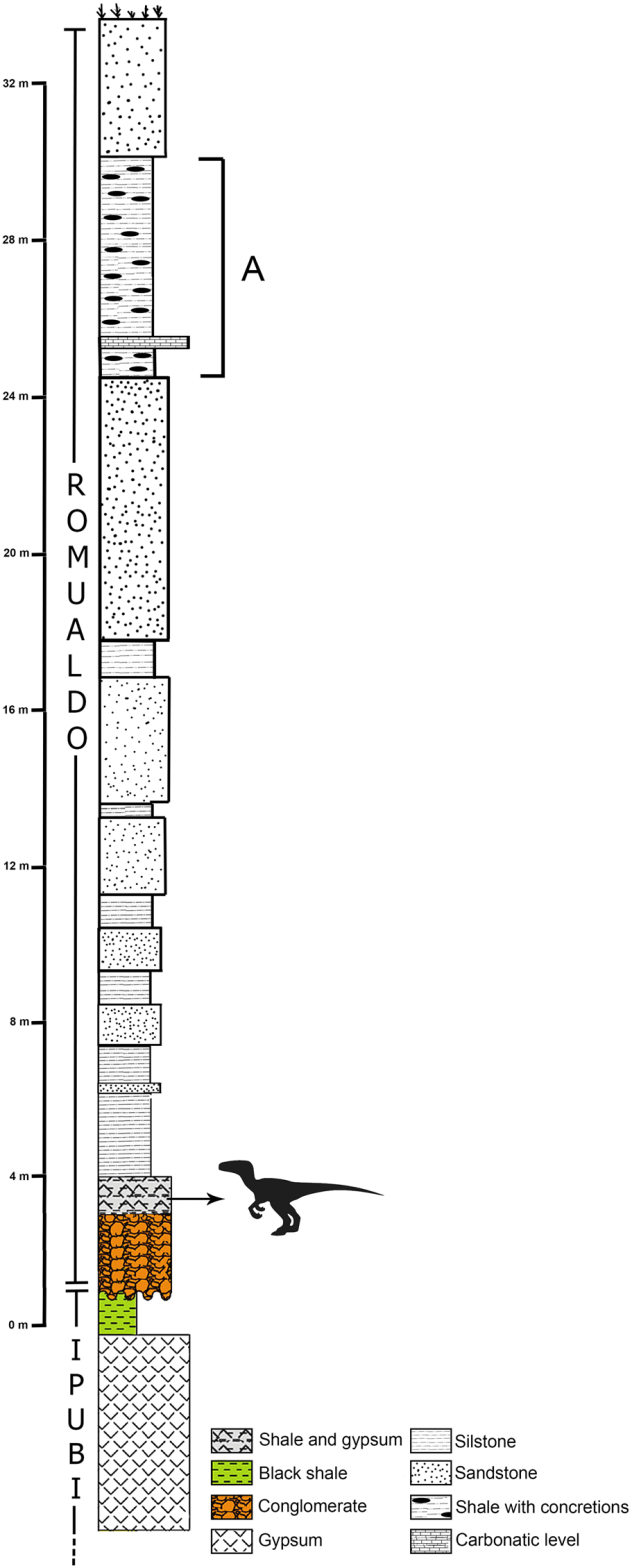
Composed stratigraphic section of Mina Pedra Branca quarry (Municipality of Santana do Cariri, Ceará State), showing the fossiliferous calcareous nodule level (A) and the dark shale horizon (dinosaur) where *Aratasaurus museunacionali* gen. et sp. nov. was collected. Figure created by Renan Alfredo Machado Bantim on PS Adobe Photoshop CC, version 20.0.6.

For about four decades, the Mina Pedra Branca is mined for gypsum and during this process exposes sections of the Ipubi and Romualdo formations (Fig. 3). The limits between these stratigraphic units is a well marked layer of conglomerate^29,33^.

A layer of dark fossiliferous shale with about 50 cm is found below the conglomerate and, regarding macrofossils, has yielded so far only coprolites, small fishes, plant material (macrocharcoal), and one turtle^35,36,37,38^.

Some 2.5 m above the conglomerate, at the base of the Romualdo Formation, another fossiliferous horizon is found. It is about 0.8 m thick and composed mainly of black shales with lenses of gypsum. This layer is positioned about 30 m below the extremely fossiliferous horizon with calcareous concretions^12,13^. A plethora of fossils, mostly undescribed, were recovered from this layer such as fishes (e.g., small clupeomorphs, large *Cladocyclus* and *Vinctifer*), and plant material. So far, the sole tetrapod known form this deposit is the dinosaur described here.

### Systematic Paleontology

Dinosauria Owen, 1842.

Theropoda Marsh, 1881.

Tetanurae Gauthier, 1986.

Coelurosauria Huene, 1914.

*Aratasaurus* gen. nov.

#### Type species

*Aratasaurus museunacionali* sp. nov., type by monotypy.

#### Etymology

From the combination of “ara” and “atá” from the Tupi language meaning born and fire, respectively; and “saurus”, from the Greek, meaning lizard.

#### Diagnosis

The same for the species.

*Aratasaurus museunacionali* new species.

*Etymology.* The species honors the Museu Nacional/Universidade Federal do Rio de Janeiro, which is the oldest scientific institution of Brazil and was recently devasted by a fire^25^.

#### Holotype

Incomplete but articulated right hind limb with the distal portion of the femur, proximal half of the tibia and mid-distal regions of metatarsals I–IV, phalanges I-1, II-1–2, III-1–3 and IV-1–4; unguals I, II and III. The specimen (MPSC R 2089) is housed at the Museu de Paleontologia of the Universidade Regional do Cariri, Santana do Cariri, Ceará State, and a cast will be deposited at the Museu Nacional/UFRJ.

#### Horizon and locality

Mina Pedra Branca, a quarry situated close to the town of Santana do Cariri, Ceará State, Northeastern Brazil. The specimen MPSC R 2089 was recovered from the lower strata of the Romualdo Formation (Aptian)^39,40^, in a dark shale located about 2.5 m above the contact with Ipubi Formation. Coordinates: S 39° 42′ 37″; W 92° 08′ 05″.

#### Diagnosis

*Aratasaurus museunacionali* differs from other basal coelurosaurs by the following combination of characters: tibia exhibiting a medial fossa; symmetric pes, with digits II and IV subequal in total length; distal condyles of metatarsi II, III and IV symmetric mediolaterally and with subequal width; width of metatarsi II and IV similar, presenting the dorsal surface of the distal articulation bulbous.

#### Description

**Figure 4:**
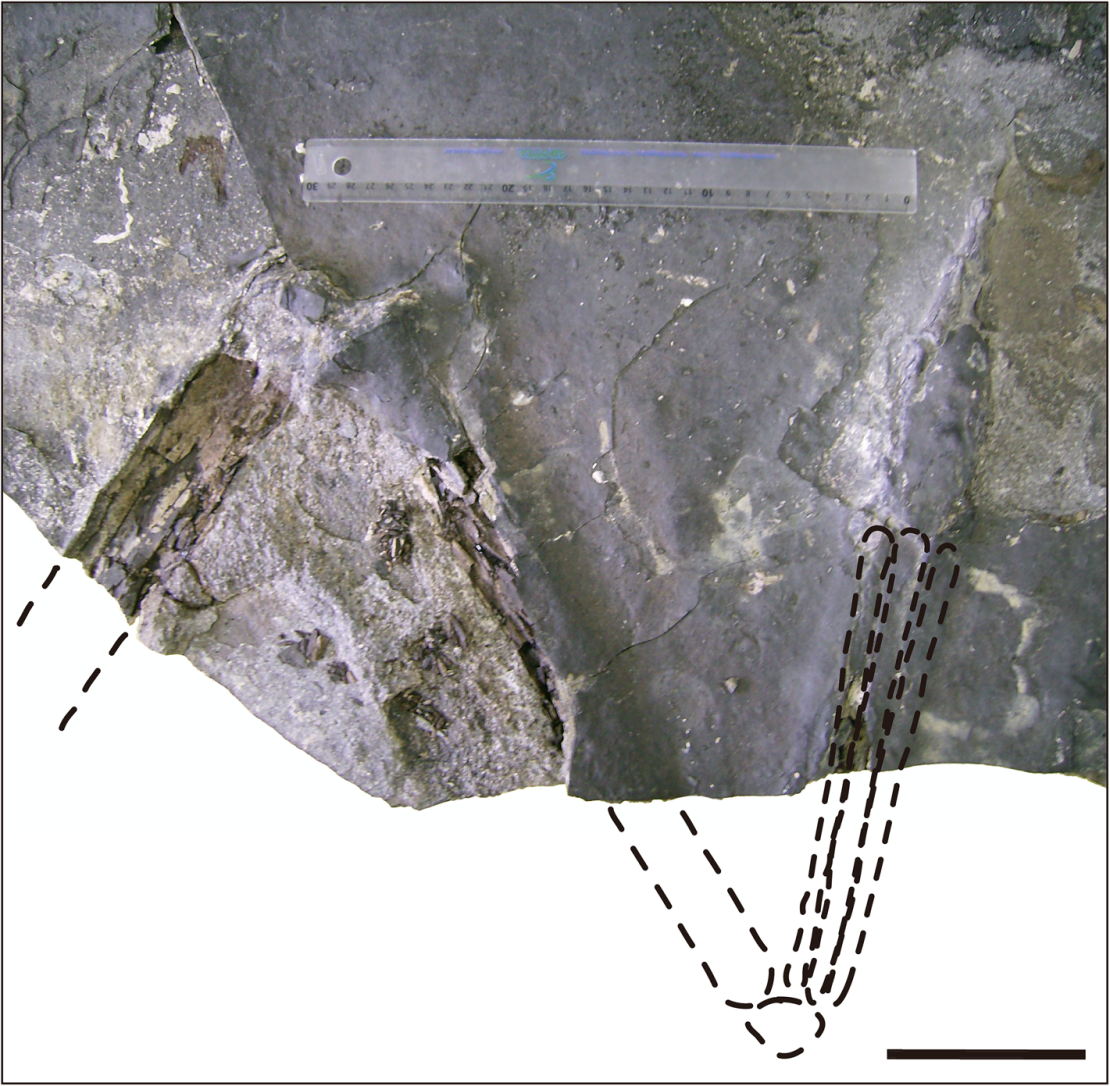
The holotype (MPSC R 2089) of *Aratasaurus museunacionali* gen. et sp. nov., showing the femur and tibia before preparation. Scale bar: 100 mm.

The specimen MPSC R 2089 was found in one slab and was articulated (Fig. 4). It consists of a hind limb including the pes. The tibia and most of the femur were complete and broken during the mining activity, suggesting that more of this individual was originally preserved, as commonly found in fossil material preserved in dark shales. Both femur and tibia were compacted during the fossilization and showed a cracked external bone surface, while the elements of the pes, especially the phalanges, still showed most of their original tridimensional shape.

**Figure 5:**
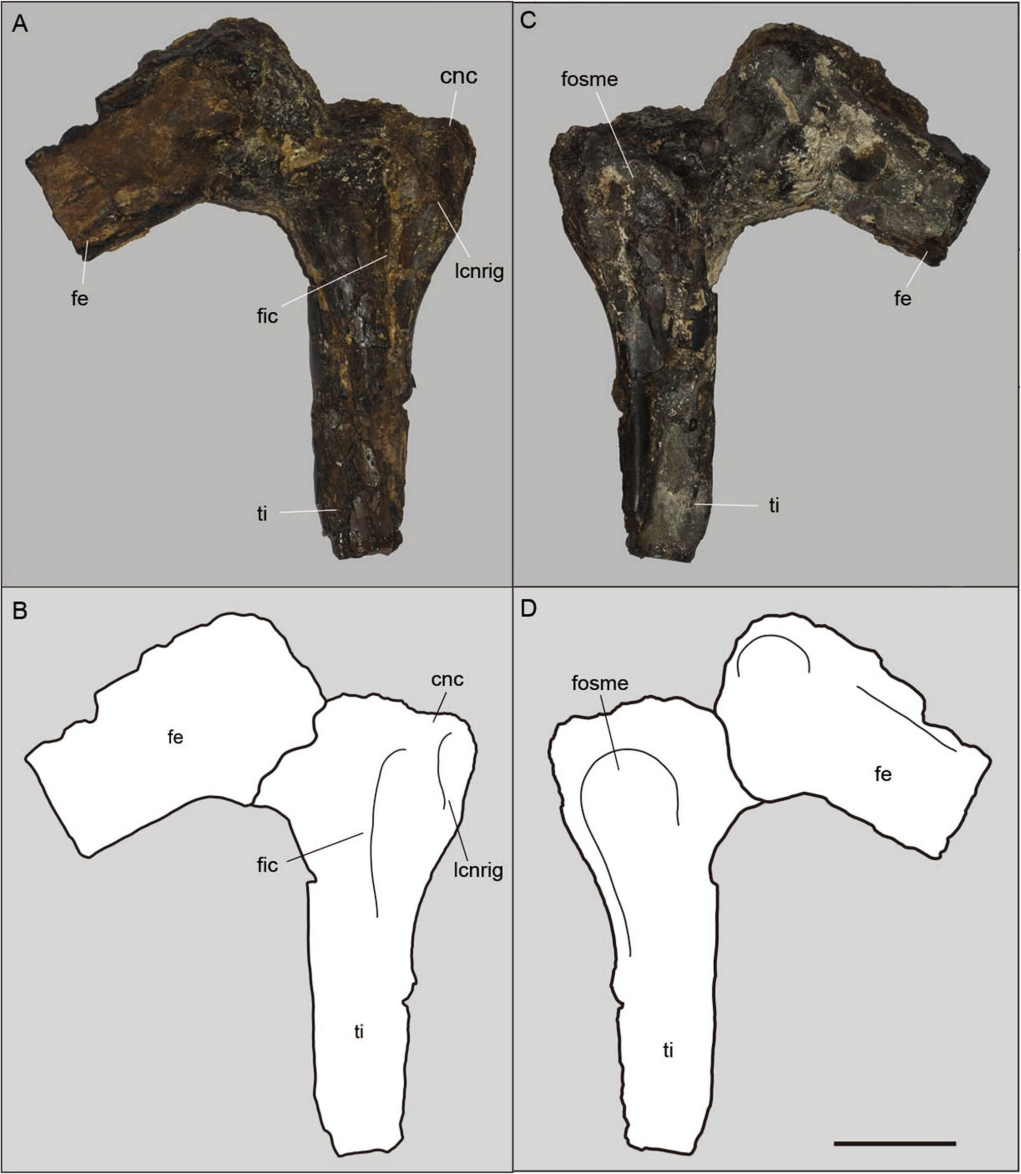
The holotype (MPSC R 2089) of *Aratasaurus museunacionali* gen. et sp. nov., photos and drawings from the lateral (A, B) and (C, D) medial views. Abbreviations: cnc, cnemial crest; fe, femur; fosme, fossa medial; lcnrig, lateral cnemial rigde; ti, tibia; tic, tibia crest. Scale bar: 50 mm.

Only a section of the distal portion of the femur is preserved (110 mm; Fig. 5). It is observable only from the medial view. The most distal region is in articulation with the proximal surface of the tibia, covering most of the posterior intercondylar fossa. A deep intercondylar fossa is observable. A marked groove separates the condyles.

The proximal half of the tibia is preserved, with a total preserved length of 175 mm. Based on the position of this element and the pes, the complete bone was about 412 mm (Fig. 4; Table 1). The proximal region is aligned with the main shaft. The cnemial crest is at the same level of the fibular condyle and poorly projected anteriorly (Fig. 5). It is bulbous and exhibits a lateral ridge. The fibular condyle forms a right angle with the anteroposterior axis of the articulation. The medial surface of the tibia is marked by a fossa, located close to the proximal articulation. In medial view, the fibular condyle is continuous with the fibular crest. A deep fossa separates the lateral cnemial ridge from the fibular crest.

**Table 1:** Measurements (mm) of the bones of the pes of *Aratasaurus museunacionali* gen. et sp. nov.

Bone element	Length	Width of the proximal condyle	Width of distal condyle
Tibia	412est		
Metatarsus I	25*		
Metatarsus II	114*	–	22
Metatartus III	153*		25
Metatarsal III	243est		
Metatarsus IV	118*		24
Phalanx I-1	23	–	
Phalanx II-1	62	22	19
Phalanx II-2	40	19	19
Phalanx III-1	65	24	21
Phalanx III-2	48	20	18
Phalanx III-3	41	19	15
Phalanx IV-1	42	24	
Phalanx IV-2	32		
Phalanx IV-3	17		14
Phalanx IV-4	17	12	12
Ungual I	10		–
Ungual II	30	11	–
Ungual III	32	12	–

*Preserved length, *est* estimated length.

**Figure 6:**
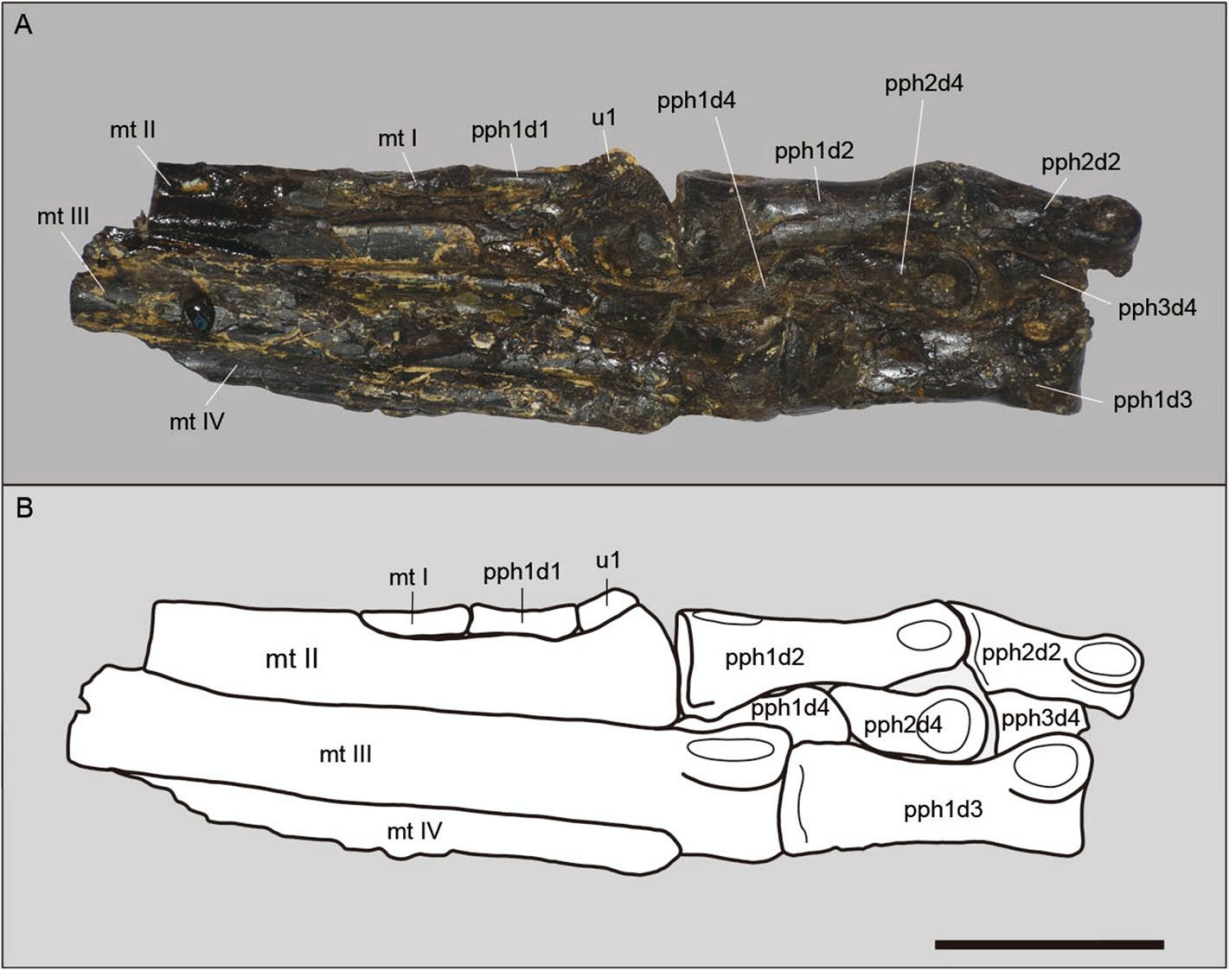
Part of the holotype (MPSC R 2089) of *Aratasaurus museunacionali* gen. et sp. nov., showing the (A) photo and (B) drawing of the right pes. Abbreviations: mt I-IV, metatarsal I-IV; pph1d1; pph1-2d2, first to second phalanx of pedal digit II; pph1d3, first phalanx of pedal digit III; pph1-3d4, first to third phalanx of pedal digit IV; u1, ungual I. Scale bar: 50 mm.

An incomplete pes is preserved (Fig. 6, Table 1). It is elongated and slender with straight metatarsi. Metatarsus I is almost complete while only the distal half of metatarsals II, III and IV are preserved. The digits are almost complete, lacking only digit V and the ungual IV.

Metatarsal I is elongated and thin. In comparison with the other metatarsals, it is reduced and has the same length as the first phalange of pedal digit I. The proximal articulation is flattened and blade-like. This bone contacts the mid-distal region of the medial surface of metatarsal II. The distal condyle is symmetrical.

Metatarsals II and IV are morphologically and proportionally similar, being expanded mediolaterally. All exhibit collateral ligament pits. The longest is metatarsal III, which, based on the relation of the foot relative to the tibia, was about 243 mm long (Fig. 4). The dorsal surface of the distal articulation of metatarsals II and IV are bulbous, being smoother in the latter. The articulation of metatarsal III is markedly ginglymoid, with an extensor pit on the dorsal surface. The collateral ligament pits are present in all metatarsals, being deeper in metatarsal III and shallower in metatarsal IV.

Digits II and IV are about the same length. The preserved pedal phalangeal formula is I-1, II-2, III-3 and IV-4 (Fig. 6). Although most of the phalanges are compressed, some of the ones of digit III were preserved in their original shape and exhibit an ellipsoid cross-section. They are long and slender, with a shortening of the distal phalanges, with digit IV possessing the shortest phalanges compared to the remaining digits. The collateral ligament pits of the phalanges of digits II and III are deep and symmetrical, being deepest in the proximal phalanges. Although these pits are also deep in digit IV, they exhibit a slight mediolateral asymmetry, being deeper in the lateral side in digit III. The dorsal surface of the proximal articulation of phalanges II-1 and III-1 is bulbous. The distal articulation of phalanges II-1 and III-2 are marked by an extensor pit marks on the dorsal surface. The phalanges II-2, III-2 and III-3 and all of the digit IV show an asymmetric shaft, with the proximal half of the ventral surface showing flexor processes. The phalanges III-2 and III-3 also exhibit a concave ventral surface, being more accentuated in the latter.

**Figure 7:**
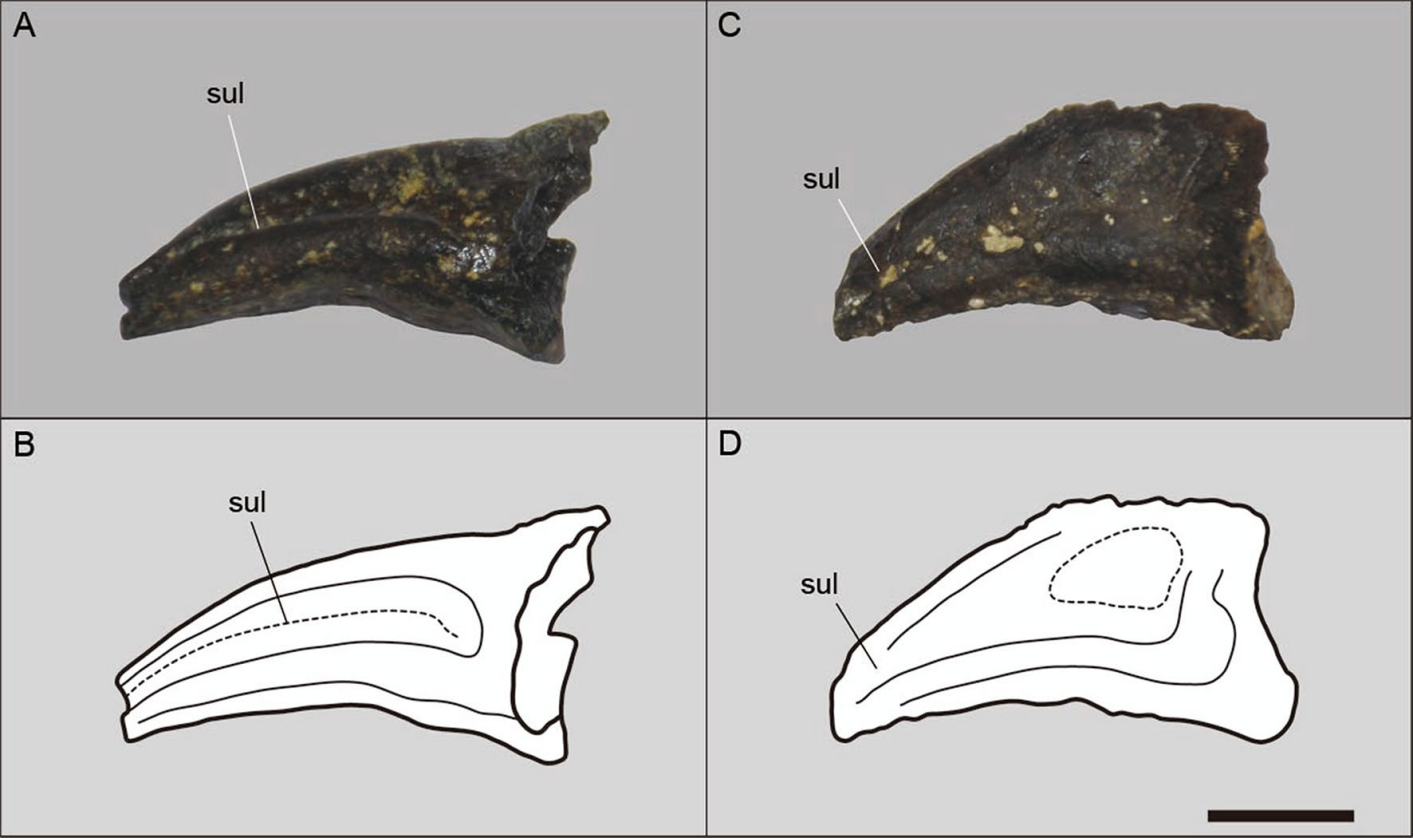
Pedal unguals (MPSC R 2089) of *Aratasaurus museunacionali* gen. et sp. nov. (A, B) Photo and schematic drawing of the second pedal digit and (C, D) photo and schematic drawing of the third pedal digit. Abbreviation: sul, sulcus. Scale bar: 10 mm.

Unguals I, II and III are preserved (Fig. 7). Most of the dorsal surface of the ungual I is covered by rock matrix. The ventral surface of all unguals show a faint flexor tubercle. The lateral and medial surfaces of the unguals II and III exhibit ridges, especially in the ungual II.

*Comparisons.* The tibia with a cnemial crest and the fibular condyle at the same level is observed in *Zuolong sallei*^41^, *Aorun zhaoi*^42^ and *Tanycolagreus topwilsoni*^43^. This feature is distinct from *Australovenator wintonensis*^44^ (see ^45^), *Tyrannosaurus rex*^46^ (see ^47^), Ornithomimosauria^48^ and Ceratosauria^49^. The rounded cnemial crest is also present in *Zuolong sallei*, *Aarun zhaoi* and *Tanycolagreus topwilsoni*. The lateral ridge on the cnemial crest is also observed in *Zuolong sallei.* A rounded fibular condyle and an elongated fibular crest is shared with *Aratasaurus museunacionali* and *Zuolong sallei*. This condyle in *Australovenator wintonensis* presents a ventral convexity, which is different from the flattened surface of *Aratasaurus museunacionali*.

Symmetric metatarsals present in the new species is also observed in *Aarun zhaoi* and *Tanycolagreus topwilsoni*, with metatarsals II and IV exhibiting a similar length, and distinct from the asymmetric condition of *Zuolong sallei*. This asymmetry was also observed in troodontids, ornithomimosaurs and tyrannosaurs^42,50^. The articulation of metatarsal II is "comma-shaped" in *Zuolong sallei*, while it is bulbous and symmetric in *Aratasaurus museunacionali*. The metatarsal III of *Aratasaurus museunacionali* is similar to *Aarun zhaoi* by lacking a flange on the anterolateral surface of the distal articulation, which is present in *Zuolong sallei*. *Aratasaurus museunacionali* differs from megaraptorans, with the last exhibiting wide metatarsal III with a deeply excavated crescent-shaped extensor fossa and the metatarsal III narrower than metatarsal II and IV in anterior view^51^. The width of the metatarsals II, III and IV are about the same in *Aratasaurus museunacionali*, while *Zuolong sallei* shows a metatarsal III twice the width of the metatarsals II and IV. As in *Tanycolagreus topwilsoni*, the distal articulation of metatarsals II and IV in *Aratasaurus museunacionali* are similar and differs from the condition of *Aarun zhaoi*, in which metatarsals II is the widest and tallest among the other metatarsi of the pes. The unguals of both *Zuolong sallei* and *Aratasaurus museunacionali* are also similar, presenting flexor tubercles and symmetrical grooves in lateral facets.

To summarize, the material known from the *Aratasaurus museunacionali* differs from derived coelurosaurian groups (e.g. Tyrannosauroidea, Ornithomimosauria) and Megaraptora mainly regarding by the cnemial crest and the disposition and morphology of metatarsals. Among basal coelurosaurs, the new Brazilian theropod has a tibia similar to that of *Zuolong salleei*, and the pes more similar with that of *Aarun zhaoi* and *Tanycolagreus topwilsoni*.

#### Osteohistology

**Figure 8:**
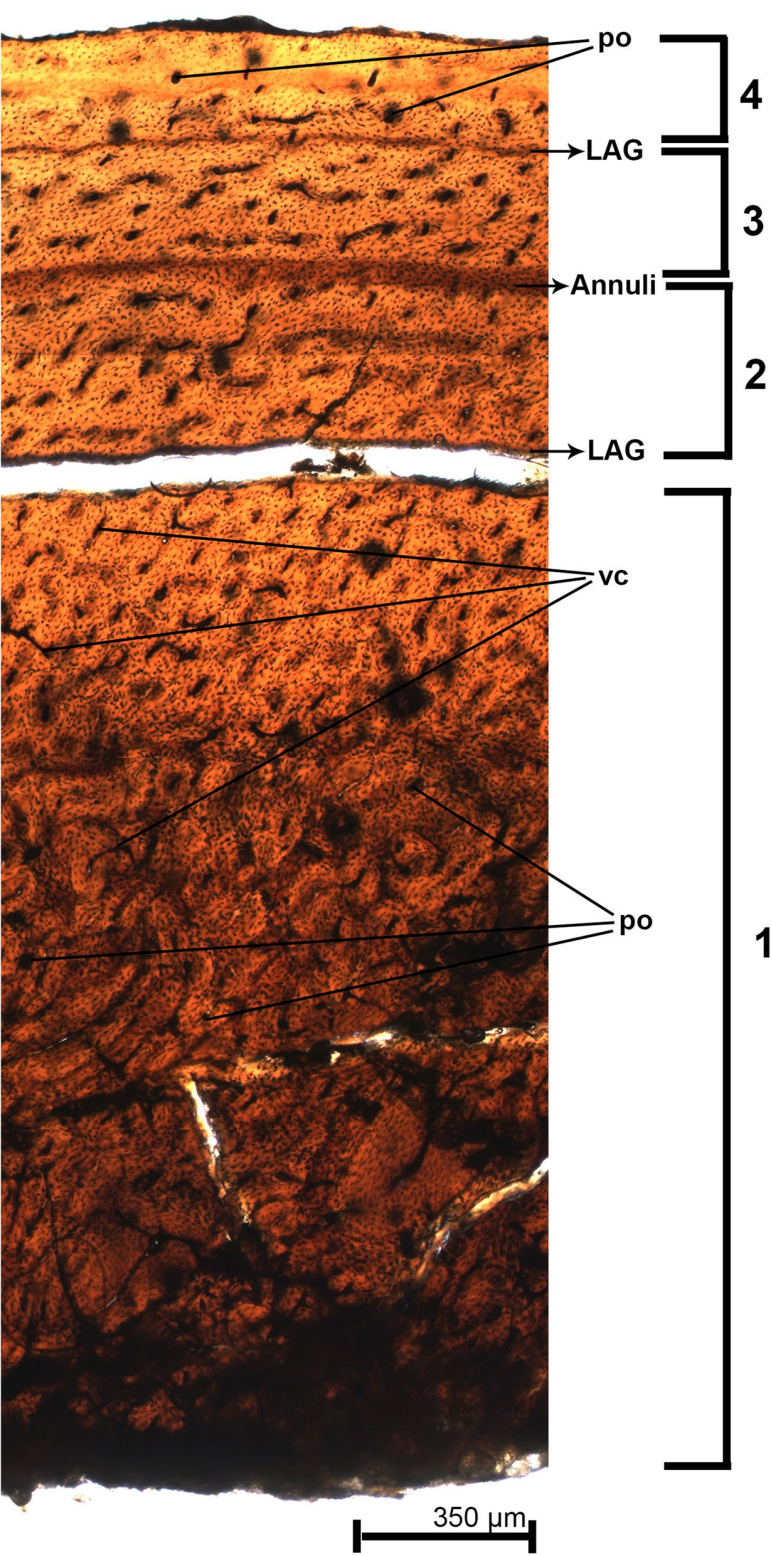
Osteohistological section of the second metatarsal of *Aratasaurus museunacionali* gen. et sp. nov., showing the four growth cycles (numbers 1–4) marked by two lines of arrested growth and one annulus. po—primary osteons; LAG—lines of arrested growth.

The primary cortex in the second metatarsal is primarily composed of fibrolamellar bone tissue. It is evidenced by random orientation of the bone fibers, which is found in animals with high metabolic rates (Fig. 8). The vascular network is present in the whole cortex, being much higher in the endosteal region, and decreases towards the outer cortex. The vascular network is composed essentially of simple vascular canals and primary osteons (which are found in different levels of development). The simple vascular canals follow a lamellar distribution along the cortex and some of these canals anastomoses with each other. The number of primary osteons decreases towards the outer cortex and they seem to form a parallel sequence in the outer portions. The osteocytes lacunae are rounded, fully distributed along the sample, and also around the primary osteons (which indicates a higher metabolic activity in these areas). Secondary osteons are present in the inner cortex, but in a lower number and in their early stages of development. This fact is due to the absence of the well-pronounced lamellae around the secondary osteons. It indicates that the remodeling process was still in its early stages. The growth marks (GMs) are represented by two lines of arrested growth (LAGs) and one annulus. The formation of LAGs indicates an effective cessation of the bone growth, whereas the annulus represents a decrease of the rate of bone deposition. The annulus is the second GM and is located in the middle portion of the cortex. The last GM is a LAG located in the outer cortex. These three GMs indicate at least four growth cycles in MPSC R 2089. No external fundamental system (EFS) was observed in the periosteal surface. The absence of this structure indicates that *Aratasaurus museunacionali* was still under active growth and had not reached the asymptotic growth at time of death.

#### Phylogenetic analysis

**Figure 9:**
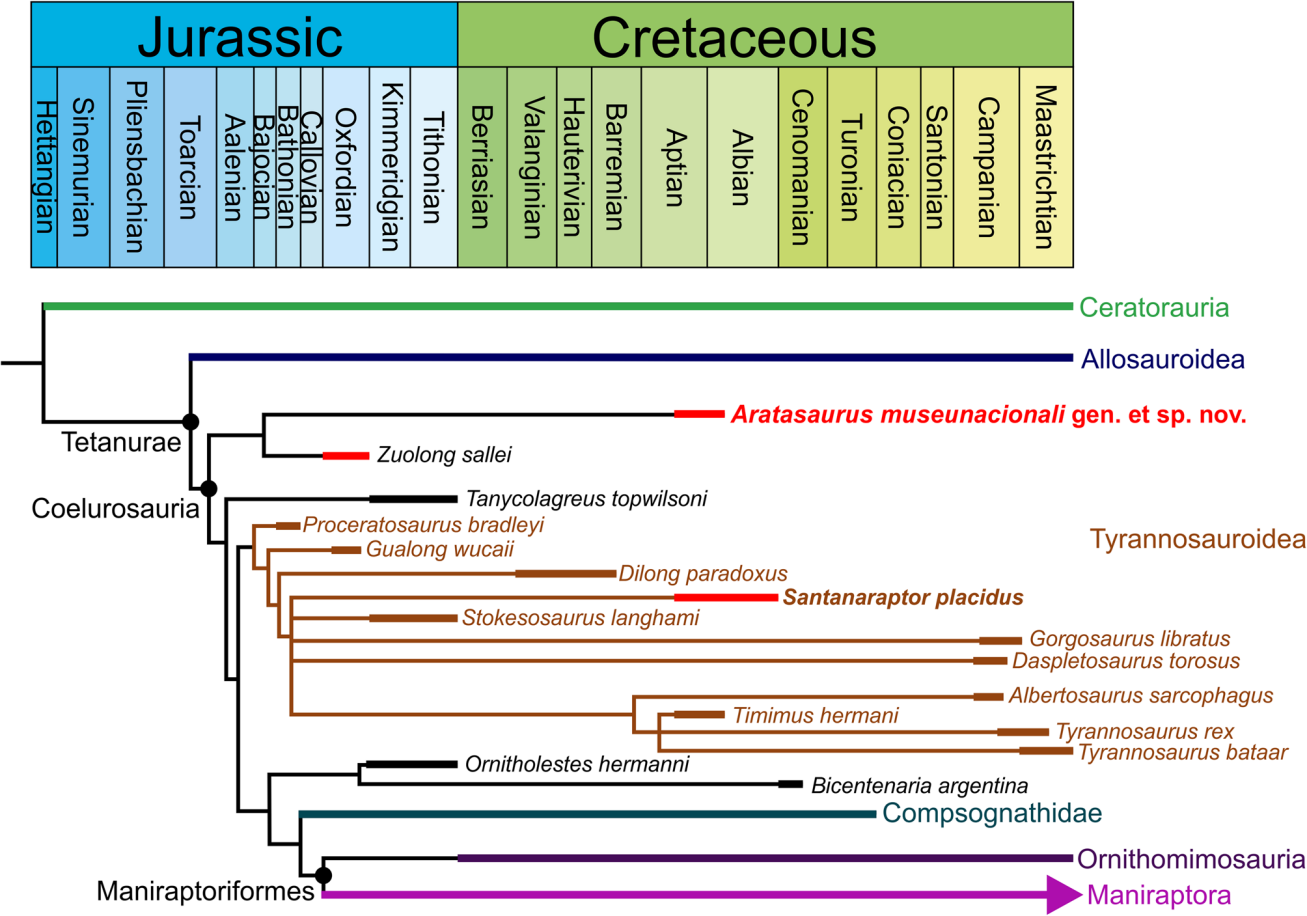
Simplified time-calibrated phylogenetic tree showing the relationships of *Aratasaurus museunacionali* gen. et sp. nov. within Tetanurae. The phylogeny is based on Choinere et al*.*^52^ for Coelurosauria, adding the codification provided by Delcourt and Grillo^53^ for *Santanaraptor placidus* and *Timimus hermani* (see supplementary information). Stratigraphic chart modified from Cohen et al. (2013).

The heuristic search resulted in 1,056 most parsimonious trees of length 2,984, with Consistency Index of 0.223 and Retention Index of 0.599 (Fig. 9). All the major groups of Theropoda were recovered as in recent analysis^52,53^. The topology was the same obtained by Delcourt and Grillo^53^, especially the resolution of basal coelurosaurs and the relationship within Tyrannosauroidea. The ambiguous synapomorphies are indicated by an asterisk (*). *Aratasaurus museunacionali* was grouped with *Zuolong salleei* at the base of Coelurosauria (Fig. 9, Supplementary Information), sharing as unambiguous synapomorphy the distal end of metatarsal III ginglymoidal (character 553:0 > 1^52^). Although no hind limb character in our analysis supported Coelurosauria, this clade was recovered by 6 unambiguous synapomorphies: (1) antorbital fossa with a dorsal border in lateral view (character 32:1 > 0^41,52^); (2) ectopterygoid deeply excavated and medial opening constricted into a foramen (character 119:2 > 3^52^); (3) premaxillary tooth crows with a D-shaped cross-section, with a flat lingual surface (character 224:0 > 1^41,52^); (4) ventral surface of anterior caudal vertebrae rounded or with a distinct keel, sometimes bearing a narrow shallow groove on its midline (character 316:2 > 0^52^); (5) brevis fossa presenting a shelf-like, narrow with subparallel margins (character 436:1 > 0^52^); (6) pubic boot with little or no anterior process (character 457:0 > 1^52^). No hind limb character supported the node Tyrannoraptora (sensu ^54^) including Tyrannosauroidea and other coelurosaurs. The four synapomorphies of this node are: (1) circular orbit in lateral or dorsolateral view (character 98:1 > 0^52^); (2) mandible with the attachment of the m. depressor mandibulae on the retroarticular process facing posterodorsally (character 206:0 > 1^52^); (3) medial side of the metacarpus II unexpanded (character 398:0 > 1^52^); (4) tibia with the medial proximal condyle arcuate and posteriorly angular in proximal view (character 513*:0 > 1^52^). The assignment of the taxon *Tanycolagreus topwinsoni* was different in our analysis from the one obtained by Choiniere et al.^42^, who found this taxon at the base of Tyrannosauroidea while we recovered it as a basal coelurosaur (Fig. 9). The assignment of *Bicentenaria argentina* was more derived than recovered by Novas et al.^55^. It was grouped with *Ornitholestes hermanni*^56^ based on two unambiguous synapomorphies: most anterior level of the jugal process of the quadratojugal, anterior to the infratemporal fenestra (character 66:0 > 1^52^) and the olecranon process of the ulna weakly developed (character 376:1 > 0^52^).

The fibular crest clearly separated from the proximal articular surface of the tibia (character 516:0 > 1^52^) supports the placement of *Aratasaurus museunacionali* within Tetanurae. Comparing with Tyrannosauroidea, *Aratasaurus museunacionali* exhibits an accessory ridge on the lateral surface of the cnemial crest, which differs from the absent condition that supports the group that includes *Dilong paradoxus* and *Tyrannosaurus* (character 510:1 > 0^52^) within tyrannosauroids. In addition, *Aratasaurus museunacionali* is also distinct from the group that unites *S. placidus* and *Tyrannosaurus* by the unexpanded medial side of the anterior surface of the distal end of metatarsus III (character 556:0 > 1*^52^ in tyrannosauroids). Therefore, based on the plesiomorphic characters and close relationships with *Zuolong salleei*, *Aratasaurus museunacionali* integrates the most basal lineage of Coelurosauria.

## Discussion

**Figure 10:**
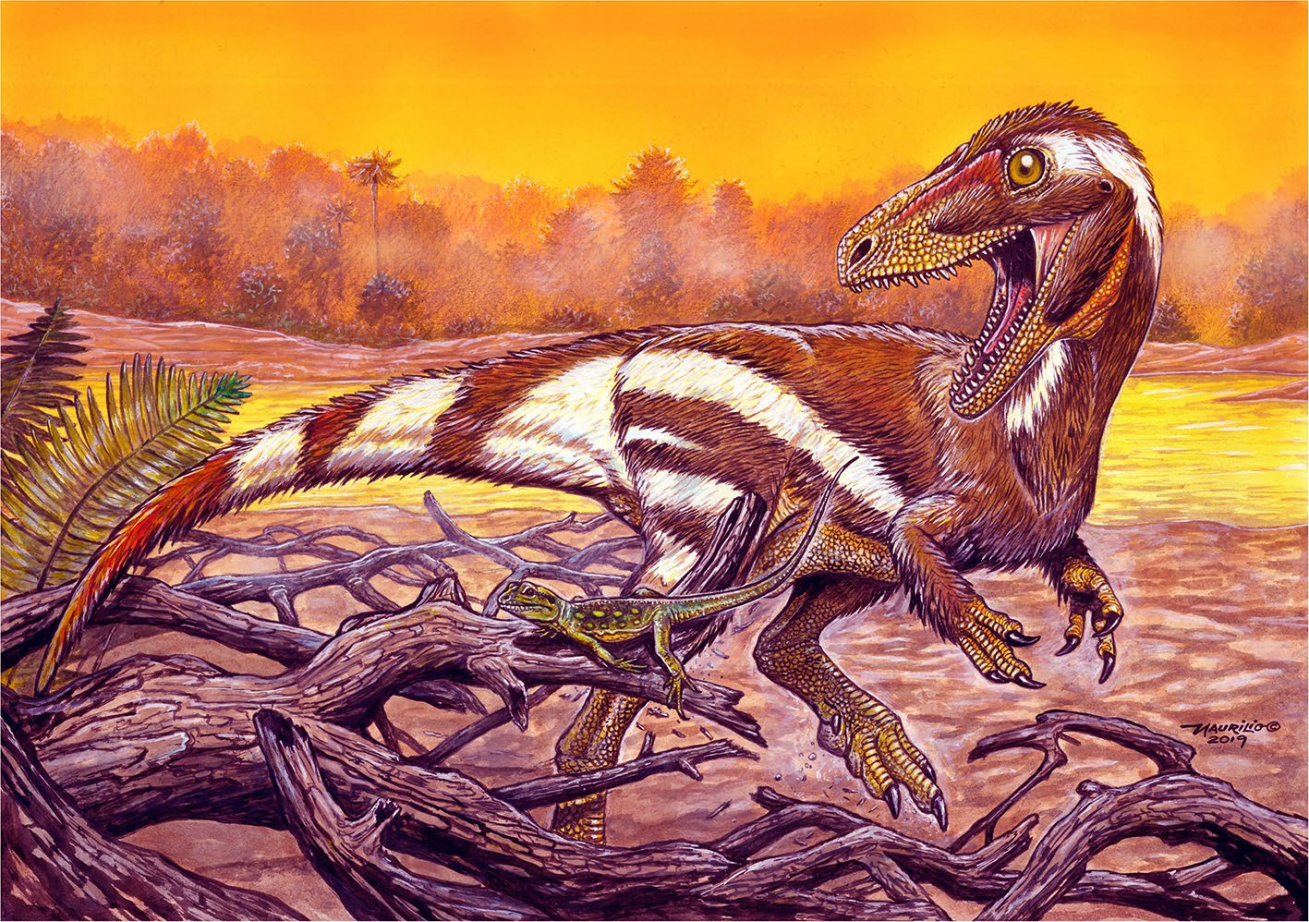
Life reconstruction of *Aratasaurus museunacionali* gen. et sp. nov.. Art work of Maurilio Oliveira.

Although the fossil material is incomplete and very compressed, the cross sections of the femur, tibia and metatarsi are similar to the ones of *Zuolong salleei*^41^, which suggests that *Aratasaurus museunacionali* exhibited a similar body dimensions, estimated in 34.25 kg of body mass and 3.12 m of total length (Fig. 10). The Brazilian species presents morphological similarity with taxa from the Upper Jurassic of Asia and North America^41,42,43^. Based on the few theropods recorded from the Romualdo Formation, there is indication that the cosmopolitism and diversification of basal coelurosaurian lineages advanced through the Lower Cretaceous, with further isolation of derived forms (e.g. tyrannosauroids^53^, dromaeosaurids^57,58^ and compsognathids^59^). This also matches with the separation between South America and Africa by the South Atlantic Sea opening in the late Albian (~ 104 Ma^60^). It should be noted that there is still a basic gap in the basal coelurosaurs, making their diversification processes unclear.

Establishing ontogenetic state is quite hard in fossils overall, including theropod dinosaurs^61^, for all based on incomplete material. However, based on osteohistology, the animal was still growing at the time of its death. Total account of growth lines is the most common method by which paleohistologists estimates age in dinosaurs^62^. These animals are known to have a higher metabolic and growth rates^63,64,65^ when compared to slow-growing archosaurs relatives like crocodyliformes^17,66,67,68^. The species *Aratasaurus museunacionali* showed four growth cycles and three growth marks throughout its growing history. The first cycle is the thickest one and it is interrupted by a LAG. Its means that *Aratasaurus museunacionali* grew continuously and faster during its first year of life. This aspect is common to other dinosaurs^69,70,71^ and Crocodylomorpha^67,68^, but unlikely in pterosaurs^72,73,74^ and birds^75^ which show a continuous and accelerated metabolic process which might erase the some of the growth cycles.

The second, third and fourth cycles gradually decrease their width and number of vascular canals. In the second cycle the animal laid down primary bone until it forms an annulus (Fig. 8). The presence of annulus means that the growth has decreased for a period, but has not stopped. The third and fourth cycles are similar in their width and separated by a LAG. The presence of the LAG indicates that growth has effectively stopped during an annual cycle. This cyclical growth pattern is common amongst dinosaurs and other outgroup clades like Actinopterygia (ray-finned fish), Amphibia (amphibians), Lepidosauria (tuatara and squamates) and Crocodylia (crocodilians)^76,77,78^. However, previous works argued that dinosaurs without growth LAGs in their skeleton are the exception rather than the rule^79^. Dinosaurs growth curve is known to be higher in the initial stages and it decreases until the animal stops to grow. When skeletal maturity is attained, the animal forms the *External Fundamental System* (EFS). This has been already detected in many clades of archosaurs (see ^80^ for a review). Specifically, in dinosaurs the EFS has been reported within many taxa^63,78,81,82^ but has not been identified in *Aratasaurus museunacionali*. The absence of EFS, secondary osteons and a high number of primary osteons implies on a juvenile/young adult ontogenetic stage for this animal, which probably was, at least, four years old at time of death. The ontogenetic stage attributed to *Aratasaurus museunacionali* probably explains its reduced proportions, when compared to its related taxa, because its asymptotic size was not reached, indicating that this animal could have grown further.

## Methods

### Heuristic tree search

We scored the *Aratasaurus museunacionali* in the dataset of Choinere et al*.*^52^ to Coelurosauria, adding the codification provided by Delcourt & Grillo^53^ to *Santanaraptor placidus*^21^ and *Timimus hermani*^83^. We also codded the Argentinean taxon *Bicentenaria argentina*^55,84^ totalizing 568 characters and 101 theropod taxa. This dataset was employed due to the inclusion *Santanaraptor placidus*, which is from upper strata of the Romualdo Formation, and to comprise best resolution of basal coelurosaurian lineages. The analysis was performed in TNT 1.1^85^ using the following parameters: hold 800,000 trees, traditional search tree bisection and reconnection (TBR) branch swapping with zero random seed, 3,000 replicates and 10 saved trees per replication. The obtained trees were reanalyzed in TBR with the parameter “stop when maxtrees hit”.

The coding of *Aratasaurus museunacionali* gen. et sp. nov. in the matrix published by Choiniere et al*.*^52^, with additional coding for *Santanaraptor placidus* and *Timimus hermani* as follows:

?????????????????????????????????????????????????????????????????????????????????????????????????????????????????????????????????????????????????????????????????????????????????????????????????????????????????????????????????????????????????????????????????????????????????????????????????????????????????????????????????????????????????????????????????????????????????????????????????????????????????????????????????????????????????????????????????????????????????????????????????????????????????????0?10?0001,010111?0??????????????????????00?000?1?00?1??01?0??000000-

The coding of *Bicentenaria argentina*^55,84^ in this matrix is as follows:

????????????????????????????????????????????????????00????10?00001???????????????????????????????????????00??0?1?110113???????????00111?1??????????????????????????????????????????????????????????????0?01???00???????0?0???0110?????0??0????????????????????????????????????????????????????????????????????010???????001?0???0????????????????????????????????????00?????????????00?0???????????????????????????????????????????????????????????00?????????100???????????????????????????????????????101100010000100100?00????????????????????????0??????????????????????????????0???

### Paleohistological protocols

In order to assess the osteohistological arrangement of *Aratasaurus museunacionali* gen. et sp. nov., the second metatarsal was sampled. All the bones were measured, photographed and described in advance, according to the methodology by Lamm^86^. Two casts were also made to preserve external morphological data. The bone was sectioned in previous existing breaking area, preserving most of the original length. A bone sample with approximately 1 cm of thickness was obtained. It was embedded in clear epoxy resin Resapol T-208 catalyzed with Butanox M50, cut with a micro rectify (Dremel 4000 with extender cable 225) mounted to a diamond disk. Then, the mounting side was wet ground and polished using a metal polishing machine (AROPOL-E, AROTEC LTDA) using AROTEC. Abrasive sandpaper of different grits were used in this step (grit size 60 / P60, 120 / P120, 320 / P400, 1200/P2500). Finally, the section was examined and photographed under a transmitted light microscope (Zeiss Inc. Barcelona, Spain) mounted to an AxioCam camera with Axio Imager, after the histological slide was prepared. The M2 imaging software was used in the examination procedure.

## Supplementary information

Supplementary Figure 1.

Supplementary material 2: The coding matrix for nexus and TNT used in the present study.

Supplementary material 3: The coding matrix for nexus and TNT used in the present study.
