# Author Correction: The first theropod dinosaur (Coelurosauria, Theropoda) from the base of the Romualdo Formation (Albian), Araripe Basin, Northeast Brazil

**DOI:** 10.1038/s41598-020-70349-8

**Published:** 2020-08-05

**Authors:** Juliana Manso Sayão, Antônio Álamo Feitosa Saraiva, Arthur Souza Brum, Renan Alfredo Machado Bantim, Rafael Cesar Lima Pedroso de Andrade, Xin Cheng, Flaviana Jorge de Lima, Helder de Paula Silva, Alexander W. A. Kellner

**Affiliations:** 1 Laboratório de Paleobiologia e Microestruturas, Centro Acadêmico de Vitória, Universidade Federal de Pernambuco, Rua Alto do Reservatório, Bela Vista, Vitória de Santo Antão, Pernambuco 55608‑680 Brazil; 2 Laboratório de Paleontologia da URCA, Universidade Regional do Cariri, Rua Carolino Sucupira, s/n, Crato, CE 63100‑000 Brazil; 3 Laboratory of Systematics and Taphonomy of Fossil Vertebrates, Departamento de Geologia e Paleontologia, Museu Nacional/Universidade Federal do Rio de Janeiro, Quinta da Boa Vista s/n, São Cristóvão, Rio de Janeiro, 20940‑040 Brazil; 4 Programa de Pós‑Graduação em Zoologia, Museu Nacional/Universidade Federal do Rio de Janeiro, Quinta da Boa Vista, São Cristóvão, Rio de Janeiro, RJ 20940‑040 Brazil; 5 College of Earth Sciences, Jilin University, Str. Jianshe 2199, Chaoyang distinct, Changchun, 130061 Jilin Province China

Correction to: *Scientific Reports*https://doi.org/10.1038/s41598-020-67822-9, published online 10 July 2020

Sayão et al. (2020), which was published electronically, does not include evidence of registration in ZooBank within the work itself, as required by Art. 8.5.3 of the amended Code of the International Commission on Zoological Nomenclature (ICZN, 1999)^1^. Therefore, the newly proposed genus-group name *Aratasaurus* and species-group name *A. museunacionali* are not available from that work. These names are established in accordance with the Code herein.

This publication has been registered in ZooBank with the LSID: urn:lsid:zoobank.org:pub:9B1D5AAB-CC72-4F1F-A132-237890E5545E. Below, we give the ZooBank LSID numbers for each of the two new names, along with the Systematic Palaeontology section of the original Article^2^.

**Systematic Paleontology**

Dinosauria Owen, 1842.

Theropoda Marsh, 1881.

Tetanurae Gauthier, 1986.

Coelurosauria Huene, 1914.

*Aratasaurus* gen. nov.

urn:lsid:zoobank.org:act:0c0664a3-2e2a-4e90-a445-c4078a0eac04

**Type species**

*Aratasaurus museunacionali* sp. nov., type by monotypy.

**Etymology**

From the combination of “ara” and “atá” from the Tupi language meaning born and fire, respectively; and “saurus”, from the Greek, meaning lizard.

**Diagnosis**

The same for the species.

*Aratasaurus museunacionali* new species.

urn:lsid:zoobank.org:act:aae5fbfc-4097-4d7a-a363-6ce54a845d49

**Etymology**

The species honors the Museu Nacional/Universidade Federal do Rio de Janeiro, which is the oldest scientific institution of Brazil and was recently devasted by a fire.

**Holotype**

Incomplete but articulated right hind limb with the distal portion of the femur, proximal half of the tibia and mid-distal regions of metatarsals I–IV, phalanges I-1, II-1–2, III-1–3 and IV-1–4; unguals I, II and III. The specimen (MPSC R 2089) is housed at the Museu de Paleontologia of the Universidade Regional do Cariri, Santana do Cariri, Ceará State, and a cast will be deposited at the Museu Nacional/UFRJ.

**Horizon and locality**

Mina Pedra Branca, a quarry situated close to the town of Santana do Cariri, Ceará State, Northeastern Brazil. The specimen MPSC R 2089 was recovered from the lower strata of the Romualdo Formation (Aptian), in a dark shale located about 2.5 m above the contact with Ipubi Formation. Coordinates: S 39° 42′ 37″; W 92° 08′ 05″.

**Diagnosis**

*Aratasaurus museunacionali* differs from other basal coelurosaurs by the following combination of characters: tibia exhibiting a medial fossa; symmetric pes, with digits II and IV subequal in total length; distal condyles of metatarsi II, III and IV symmetric mediolaterally and with subequal width; width of metatarsi II and IV similar, presenting the dorsal surface of the distal articulation bulbous.
