# Supplementary material for: The first theropod dinosaur (Coelurosauria, Theropoda) from the base of the Romualdo Formation (Albian), Araripe Basin, Northeast Brazil
Source: Sci Rep. 2020 Jul 10;10:10892. doi: 10.1038/s41598-020-67822-9 (PMC7351750; doi:10.1038/s41598-020-67822-9)
Supplement: Supplementary file 1 — Supplementary Figure 1. [file 41598_2020_67822_MOESM1_ESM.docx]

**SUPPLEMENTARY MATERIAL**

**The first theropod dinosaur (Coelurosauria, Theropoda) from the base of the Romualdo Formation (Albian), Araripe Basin, Northeast Brazil**

Juliana Manso Sayão^1,3^, Antônio Álamo Feitosa Saraiva^2^, Arthur Souza Brum^3,4^, Renan Alfredo Machado Bantim^2^, Rafael Cesar Lima Pedroso de Andrade^1^, Xin Cheng^2,5^, Flaviana Jorge de Lima^2^, Helder de Paula Silva^3^ & Alexander W.A. Kellner^3*^

^1^Laboratório de Paleobiologia e Microestruturas, Centro Acadêmico de Vitória, Universidade Federal de Pernambuco, Rua Alto do Reservatório, Bela Vista, Vitória de Santo Antão, 55608-680, Pernambuco, Brazil. ^2^Laboratório de Paleontologia da URCA, Universidade Regional do Cariri, Rua Carolino Sucupira, s/n, 63100-000, Crato, CE, Brazil. ^3^Laboratory of Systematics and Taphonomy of Fossil Vertebrates, Departamento de Geologia e Paleontologia, Museu Nacional/Universidade Federal do Rio de Janeiro, Quinta da Boa Vista s/n, São Cristóvão, Rio de Janeiro, 20940-040 Brazil. ^4^Programa de Pós-Graduação em Zoologia, Museu Nacional-Universidade Federal do Rio de Janeiro, Quinta da Boa Vista, São Cristóvão, 20940-040, Rio de Janeiro, RJ, Brazil. ^5^College of Earth Sciences, Jilin University, Str. Jianshe 2199, Chaoyang distinct, Changchun, Jilin Province, 130061, China.

^*^Correspondence and request for materials should be addressed to A.W.A.K. (email: [kellner@mn.ufrj.br](mailto:kellner@mn.ufrj.br))


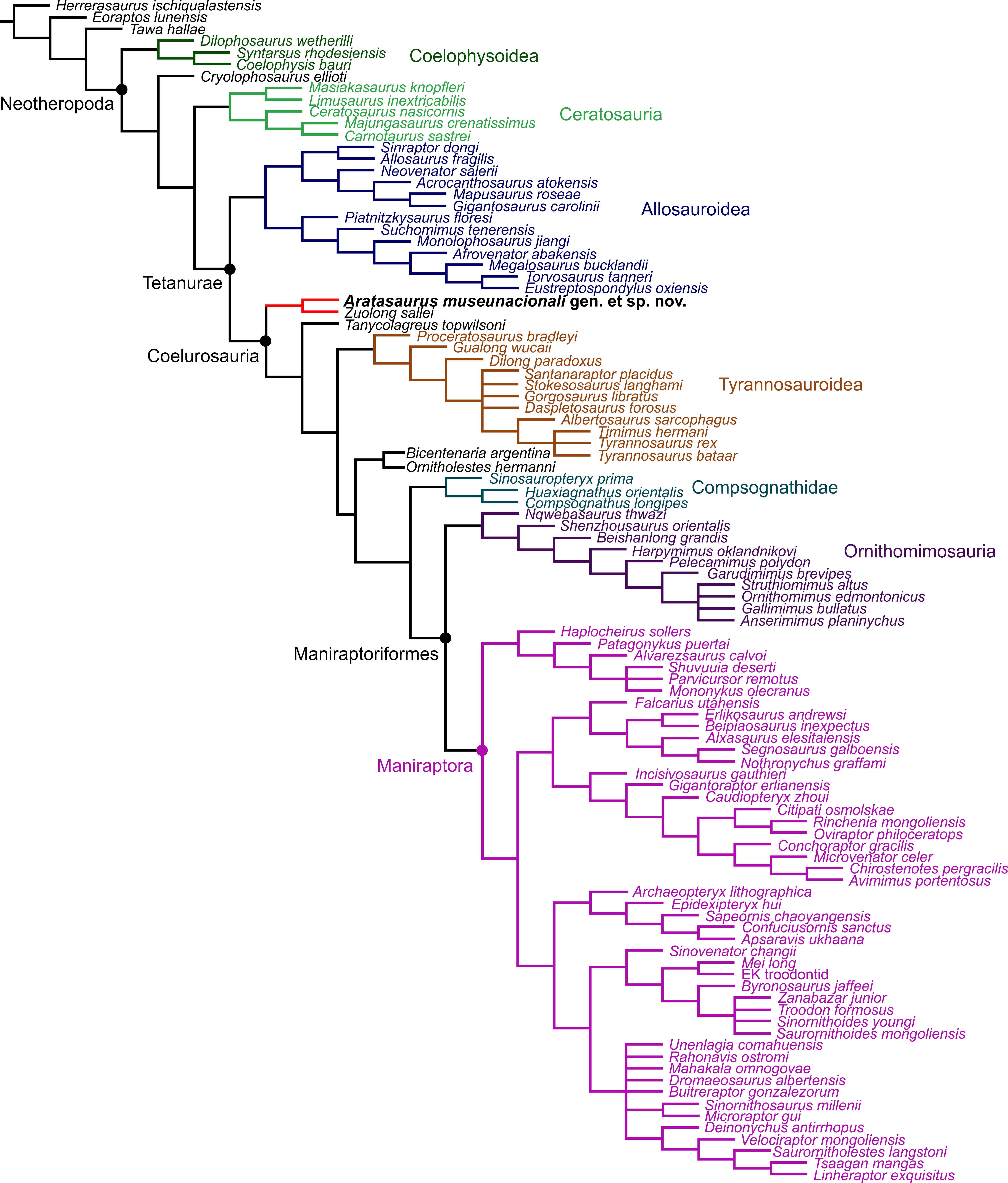


Supplementary Figure 1: complete consensus phylogenetic tree showing the relationships of *Aratasaurus museunacionali* gen. et sp. nov. The phylogeny is based on Choinere *et al.*^52^ for Coelurosauria, adding the codification provided by Delcourt & Grillo^53^ for *Santanaraptor placidus* and *Timimus hermani*, as well as the coding for the new species and *Bicentenaria argentina* provided in the present study.

Supplementary material 2 and 3: the coding matrix for nexus and TNT used in the present study.
