# Supplementary material for: The first theropod dinosaur (Coelurosauria, Theropoda) from the base of the Romualdo Formation (Albian), Araripe Basin, Northeast Brazil
Source: Sci Rep. 2020 Jul 10;10:10892. doi: 10.1038/s41598-020-67822-9 (PMC7351750; doi:10.1038/s41598-020-67822-9)
Supplement: Supplementary file 2 — Supplementary material 2: The coding matrix for nexus and TNT used in the present study. [file 41598_2020_67822_MOESM2_ESM.docx]

#NEXUS

[written Wed Apr 29 19:10:24 BRT 2020 by Mesquite version 3.40 (build 877) at PC-CASA/192.168.18.16]

BEGIN TAXA;

TITLE Taxa;

DIMENSIONS NTAX=101;

TAXLABELS

Herrerasaurus_ischigualastensis Nqwebasaurus_thwazi Acrocanthosaurus_atokensis Afrovenator_abakensis Albertosaurus_sarcophagus Allosaurus_fragilis Alvarezsaurus_calvoi Alxasaurus_elesitaiensis Anserimimus_planinychus Apsaravis_ukhaana Archaeopteryx_lithographica Avimimus_portentosus Beipiaosaurus_inexpectus Beishanlong_grandis Buitreraptor_gonzalezorum Byronosaurus_jaffeei Carnotaurus_sastrei Caudipteryx_zhoui Ceratosaurus_nasicornis Chirostenotes_pergracilis Citipati_osmolskae Coelophysis_bauri Compsognathus_longipes Conchoraptor_gracilis Confuciusornis_sanctus Cryolophosaurus_ellioti Daspletosaurus_torosus Deinonychus_antirrhopus Dilong_paradoxus Dilophosaurus_wetherilli Dromaeosaurus_albertensis EK_troodontid Eoraptor_lunensis Epidexipteryx_hui Erlikosaurus_andrewsi Eustreptospondylus_oxiensis Falcarius_utahensis Gallimimus_bullatus Garudimimus_brevipes Giganotosaurus_carolinii Gigantoraptor_erlianensis Gorgosaurus_libratus Guanlong_wucaii Haplocheirus_sollers Harpymimus_okladnikovi Huaxiagnathus_orientalis Incisivosaurus_gauthieri Limusaurus_inextricabilis Linheraptor_exquisitus Mahakala_omnogovae Majungasaurus_crenatissimus Mapusaurus_roseae Masiakasaurus_knopfleri Megalosaurus_bucklandii Mei_long Microraptor_gui Microvenator_celer Monolophosaurus_jiangi Mononykus_olecranus Neovenator_salerii Nothronychus_graffami Ornitholestes_hermanni Ornithomimus_edmontonicus Oviraptor_philoceratops Parvicursor_remotus Patagonykus_puertai Pelecanimimus_polyodon Piatnitzkysaurus_floresi Proceratosaurus_bradleyi Rahonavis_ostromi Rinchenia_mongoliensis Sapeornis_chaoyangensis Saurornithoides_mongoliensis Saurornitholestes_langstoni Segnosaurus_galboensis Shenzhousaurus_orientalis Shuvuuia_deserti Sinornithoides_youngi Sinornithosaurus_millenii Sinosauropteryx_prima Sinovenator_changii Sinraptor_dongi Stokesosaurus_langhami Struthiomimus_altus Suchomimus_tenerensis Syntarsus_rhodesiensis Tanycolagreus_topwilsoni Tarbosaurus_bataar Tawa_hallae Torvosaurus_tanneri Troodon_formosus Tsaagan_mangas Tyrannosaurus_rex Unenlagia_comahuensis Velociraptor_mongoliensis Zanabazar_junior Zuolong_salleei Santanaraptor_placidus Timimus_hermani Bicentenaria_argentina Aratasaurus_museunacionali

;

END;

BEGIN CHARACTERS;

TITLE Character_Matrix;

DIMENSIONS NCHAR=568;

FORMAT DATATYPE = STANDARD GAP = - MISSING = ? SYMBOLS = " 0 1 2 3 4 5 6";

CHARSTATELABELS

1 Contour_feathers / absent present,

2 Vaned_feathers_on_forelimb / symmetric asymmetric,

3 'Shape of premaxillary body (portion in front of the external naris)' / 'wider than high, or approx. as wide as high' significantly_higher_than_wide,

4 Premaxillae / unfused fused,

5 'Premaxillary-nasal suture dorsal view' / 'v-shaped' 'w-shaped',

6 'Premaxillary-maxillary suture' / scarf_or_butt_joint interlocking_joint,

7 Premaxillary_body_in_front_of_external_nares / rostrocaudally_shorter_than_body_below_nares_and_angle_between_anterior_margin_and_alveolar_margin_more_than_75_degrees 'rostrocaudally longer than body below the nares and angle less than 70 degrees, naris overlaps premaxillary tooth row' 'much longer than body below naris, naris located posterior to premaxillary tooth row',

8 'Ventral process at the posterior end of premaxillary body (gives the posterior process a forked appearance in lateral view)' / absent present,

9 Maxillary_process_of_premaxilla / contacts_nasal_to_form_posterior_border_of_nares reduced_so_that_maxilla_participates_broadly_in_external_naris extends_posteriorly_to_separate_maxilla_from_nasal__posterior_to_nares,

10 Internarial_bar_ / dorsoventrally_rounded dorsoventrally_flat,

11 Crenulate_margin_on_buccal_edge_of_premaxilla_ / absent present,

12 Caudal_margin_of_naris / farther_rostral_than_the_rostral_border_of_the_antorbital_fossa nearly_reaching_or_overlapping_the_rostral_border_of_the_antorbital_fossa,

13 Premaxillary_symphysis_ / 'acute, V-shaped' 'rounded, U-shaped',

14 Subnarial_foramen / absent present,

15 'Groove on lateral surface of premaxilla, extending ventrally from the narial fossa' / absent present,

16 Maxillary_fenestra / absent present,

17 Maxillary / fenestra recessed within a 'shallow,' caudally or '^ncaudodorsally' open 'fossa,' which is itself located within the maxillary antorbital fossa absent present,

18 Longitudinal_position_of_maxillary_fenestra / situated_at_rostral_border_of_antorbital_fossa situated_posterior_to_rostral_border_of_antorbital_fossa,

19 Latitudinal_position_of_maxillary_fenestra / 'situated approximately mid-height of the antorbital fossa' displaced_dorsally_in_antorbital_fossa,

20 Foramen_on_caudal_edge_interfenestral_bar_between_the_maxillary_and_antorbital_fenestrae / absent 'present, pierces ventral portion of bar',

21 'Promaxillary fenestra (fenestra promaxillaris)' / absent present,

22 Palate_formed_by / premaxilla_only 'premaxilla, maxilla and vomer',

23 Palatal_shelf_of_maxilla_ / flat 'with midline ventral "tooth-like" projection',

24 Ventrolateral_margin_of_the_maxilla_posterior_to_ascending_process / flat_or_rounded_as_it_grades_onto_tooth_row 'developed as a sharp, ventrolaterally-projecting ridge',

25 Anteroposterior_length_of_palatal_shelf_of_maxilla / short 'long, with extensive palatal shelves',

26 Orientation_of_the_maxillae_towards_each_other_as_seen_in_dorsal_view / acutely_angled subparallel,

27 Ascending_process_of_the_maxilla / confluent_with_anterior_rim_of_maxillary_body_and_gently_sloping_posterodorsally offset_from_anterior_rim_of_maxillary_body,

28 Form_of_anterior_projection_of_maxilla / 'offset from anterior rim of maxillary body, with anterior projection of maxillary body shorter than high' 'offset from anterior rim of maxillary body, with anterior projection of maxillary body as long as high or longer',

29 Ascending_process_of_maxilla / 'prominent, exposed laterally and medially' 'weakly developed, lacking lateral exposure and only slight medial exposure',

30 Anterior_margin_of_maxillary_antorbital_fossa / rounded_or_pointed square,

31 Dorsal_border_of_the_internal_antorbital_fenestra_lateral_view / formed by lacrimal 'and^n' maxilla formed_by_nasal_and_lacrimal,

32 Dorsal_border_of_the_antorbital_fossa_lateral_view / formed by lacrimal 'and^n' maxilla formed_by_nasal_and_lacrimal 'formed by maxilla, premaxilla and lacrimal',

33 Lateral_exposure_of_lamina_of_the_ventral_ramus_of_nasal_process_of_maxilla / 'present,' large '^nbroad' exposure 'present, reduced to small triangular exposure',

34 Maxillary_antorbital_fossa_in_front_of_the_internal_antorbital_fenestra / 40%_or_less_of_the_length_of_the_external_antorbital_fenestra more_than_40%_of_the_length_of_the_external_antorbital_fenestra,

35 Extent_of_antorbital_fossa_on_jugal_ramus_of_maxilla / less_than_half_the_dorsoventral_height_of_jugal_ramus more_than_half_dorsoventral_height_of_jugal_ramus,

36 'Maxilla, pneumatic region on medial side of maxilla posteroventral to maxillary fenestra' / absent present,

37 Horizontal_ridge_on_the_lateral_surface_of_maxilla_at_the_ventral_border_of_the_antorbital_fossa / absent present,

38 Medial_constriction_between_articulated_premaxillae_and_maxillae_in_dorsal_or_ventral_view / absent present,

39 Subnarial_gap_between_maxilla_and_premaxilla_at_the_alveolar_margin / absent present,

40 Maxillary_paradental_plates / unfused fused,

41 'Medial surface of maxillary paradental (interdental) plates' / smooth_or_finely_pitted dorsoventrally_striated,

42 'Maxillary paradental (interdental) plates, ventral extent' / to_the_same_ventral_level_as_lateral_maxillary_wall dorsal_to_ventral_level_of_maxillary_wall,

43 'Maxillary paradental plates, dorsal margin of anterior end' / horizontal inclined_anteroventrally,

44 Ventral_edge_of_maxillary_body_and_ventral_ramus / ventrally_flat ventrally_convex,

45 Nasals / unfused fused,

46 Dorsal_surface_of_the_nasals / smooth rugose,

47 Nasal_crest / absent 'present, single median crest' 'present, bilateral crests along lateral nasal margins',

48 Pneumatic_foramen_in_ventrolateral_margins_of__the_nasals / absent present,

49 Shape_of_nasals / expanding_posteriorly of_subequal_width_throughout_their_length,

50 'Pronounced lateral rims of the nasals, sometimes bearing lateral cranial crests' / absent present,

51 External_nares / facing_laterally facing_anterolaterally,

52 Length_of_nares / less_than_20_percent_skull_length greater_than_20_percent_skull_length,

53 Jugal_pneumatic_recess_in_posteroventral_corner_of_antorbital_fossa_ / present absent,

54 Medial_jugal_foramen / present_on_medial_surface_ventral_to_postorbital_bar absent,

55 Sublacrimal_part_of_jugal / tapering bluntly_squared_anteriorly expanded,

56 Anterior_end_of_jugal / reaches_internal_antorbital_fenestra excluded_from_the_internal_antorbital_fenestra,

57 Form_of_anterior_end_of_jugal / without_anterior_process_underneath_antorbital_fenestra expressed_at_the_rim_of_the_internal_antorbital_fenestra_and_with_a_distinct_process_that_extends_anteriorly_underneath_it,

58 Jugal_antorbital_fossa / absent_or_developed_as_a_slight_depression 'large, crescentic depression on the anterior end of the jugal',

59 Jugal / 'broad, plate-like' 'very slender, rod-like',

60 Jugal_contribution_to_postorbital_bar / contribute_equally_to_postorbital_bar ascending_process_of_jugal_reduced,

61 Anteroposterior_width_of_postorbital_bar / subequal_to_preorbital_bar 'expanded, greater than twice width of preorbital bar',

62 Rugosity_on_ventrolateral_surface_of_jugal_below_orbit / absent present,

63 Jugal_and_quadratojugal_ / separate fused_and_not_distinguishable_from_one_another,

64 Quadratojugal / 'hook-shaped, with a dorsoventrally tall, mediolaterally short process that wraps around the lateral margin of the quadrate and is visible in posterior view' 'with a dorsoventrally short, anteroposteriorly long process only visible in lateral view',

65 Quadratojugal_and_quadrate / sutural_connection_present sutural_connection_absent,

66 Anteriormost_level_of_jugal_process_of_quadratojugal_relative_to_infratemporal_fenestra / ventral_to anterior_to,

67 Supraorbital_crests_on_lacrimal_in_adult_individuals / absent present,

68 Form_of_supraorbital_crests / dorsal_crest_above_orbit lateral_expansion_anterior_and_dorsal_to_orbit,

69 Enlarged_foramen_or_foramina_opening_laterally_at_the_angle_of_the_lacrimal / absent present,

70 Lacrimal_foramen_number / single paired,

71 Lacrimal_foramina / exposed_laterally_ developed_within_a_pocket_formed_by_a_lateral_lacrimal_sheet_of_bone_and_a_rostrally_open_pocket_in_the_lacrimal_angle,

72 Height_of_the_lacrimal / 'significantly less than height of the orbit, and usually fails to reach the ventral margin of the orbit' 'as high as the orbit, and contacts jugal at the level of the ventral margin of orbit',

73 Orientation_of_jugal_ramus_of_lacrimal / strongly_sloping_anteroventrally subvertical sloping_posteroventrally,

74 Dorsoventral_thickness_of_maxillary_ramus_of_lacrimal / 'very slender, much less than anteroposterior thickness of jugal ramus' 'moderate, less than or subequal to anteroposterior thickness of jugal ramus' greater_than_anteroposterior_thickness_of_jugal_ramus,

75 Suborbital_spur_on_posterior_edge_of_ventral_ramus_of_lacrimal / absent present,

76 Lacrimal_posterodorsal_process / absent present,

77 Length_of_lacrimal_posterodorsal_process / subequal_in_length_to_maxillary_ramus much_shorter_than_maxillary_ramus,

78 Direction_of_lacrimal_posterodorsal_process / projects_horizontally projects_posterodorsally_or_completely_dorsally,

79 Passage_of_the_nasolacrimal_duct / leading_through_the_body_of_the_ventral_process_of_the_lacrimal 'ventral process of lacrimal not pierced, lateral side depressed below the level of the surrounding bones, and nasolacrimal duct passes lateral to the process',

80 Jugal_ramus_of_lacrimal / 'broadly triangular, articular end nearly twice as wide anteroposteriorly as lacrimal body at lacrimal angle' 'strut-like, roughly same width anteroposteriorly throughout ventral ramus',

81 Prefrontal / absent present,

82 Size_of_prefrontal / 'small, forms anterolateral rim of orbit with descending process proceeding along medial surface of the descending process of the lacrimal' 'small, forms small portion of skull roof and not expressed at orbital margin, no descending process' 'hypertrophied, forms portion of orbital rim and skull roof, with descending process',

83 Configuration_of_lacrimal_and_frontal / lacrimal_separated_from_frontal_by_prefrontal lacrimal_contacts_frontal,

84 Frontals / narrow_anteriorly_as_a_wedge_between_nasals 'end abruptly anteriorly, suture with nasal transversely oriented' 'nasals extend further medially than laterally, invading anteromedial contact between frontals',

85 Frontal_supratemporal_fossa / limited_extension_of_supratemporal_fossa_onto_frontal supratemporal_fossa_coovers_most_of_postorbital_process_of_the_frontal_and_extends_anteriorly_onto_the_dorsal_surface_of_the_frontal,

86 'Groove on orbital rim of frontal, possibly for reception of frontal process of postorbital' / absent present,

87 Anterior_emargination_of_supratemporal_fossa_on_frontal_ / straight_or_slightly_curved strongly_sinusoidal_and_reaching_onto_postorbital_process,

88 'Frontal postorbital process (dorsal view): ' / smooth_transition_from_orbital_margin sharply_demarcated_from_orbital_margin,

89 Orbital_margin_of_frontal / without_groove with_groove_for_articulation_with_frontal_process_of_the_postorbital,

90 Frontal_edge / smooth_in_region_of_lacrimal_suture edge_notched,

91 Postorbital_in_lateral_view_ / 'with straight anterior (frontal) process' frontal_process_curves_anterodorsally_and_dorsal_border_of_temporal_bar_is_dorsally_concave,

92 Lateral_surface_of_anterior_process_of_postorbital / thin_and_unornamented dorsoventrally_thickened_into_a_laterally_projecting_and_rugose_platform,

93 Contact_between_lacrimal_and_postorbital / absent present,

94 'Cross-section of the ventral process of the postorbital' / triangular 'U-shaped',

95 Jugal_process_of_the_postorbital / ventrally_directed_and_tapering with_suborbital_anterior_spur,

96 Postorbital_jugal_process_anterior_suborbital_spur / small large_curving_flange,

97 'Supraorbital shelf formed mostly by an additional ossification (palpebral)' / absent present,

98 Orbit / circular_in_lateral_or_dorsolateral_view dorsoventrally_long,

99 Parietals / separate fused,

100 Parietal_supratemporal_fenestra / separated_by_a_horizontal_plate_formed_by_the_parietals 'contact each other posteriorly, but separated anteriorly by an anteriorly widening triangular plate formed by the parietals' nearly_confluent_over_parietals_and_only_separated_by_a_thin_line_of_bone_along_the_sagittal_suture,

101 Anteromedial_corner_of_supratemporal_fossa / open_dorsally roofed_by_shelf_of_frontoparietal,

102 Sagittal_crest / dorsal_surface_of_parietals_smooth_with_no_sagittal_crest sagittal_crest_present,

103 Form_of_sagittal_crest / parietals_dorsally_convex_with_very_low_sagittal_crest_along_midline 'dorsally convex with well-developed sagittal crest',

104 'Posteriorly placed, knob-like dorsal projection of the parietals' / absent present,

105 Connections_of_quadratojugal_process_of_squamosal / contacts_quadratojugal does_not_contact_quadratojugal,

106 Infratemporal_fenestra_shape / 'rectangular, postorbital bar parallels quadratojugal and squamosal articular area' lower_temporal_fenestra_constricted_mesially_by_squamosal_and_quadratojugal_approaching_postorbital_bar,

107 Shape_of_quadratojugal_process_of_the_squamosal / tapering 'broad, and usually somewhat expanded',

108 Posterolateral_shelf_on_squamosal_overhanging_quadrate_head_ / absent present,

109 Quadrate_head / covered_by_squamosal_in_lateral_view quadrate_cotyle_of_squamosal_open_laterally_exposing_quadrate_head,

110 Descending_process_of_squamosal_ / parallels_quadrate_shaft nearly_perpendicular_to_quadrate_shaft,

111 Supratemporal_fenestra / bounded_laterally_and_posteriorly_by_the_squamosal supratemporal_fenestra_extended_as_a_fossa_on_to_the_dorsal_surface_of_the_squamosal,

112 Quadrate / solid hollow,

113 Mandibular_joint / approximately_straight_below_quadrate_head significantly_posterior_to_quadrate_head significantly_anterior_to_quadrate_head,

114 'Quadrate medial pneumatic recess (depression and foramen in the area of the mandibular condyle on medial surface)' / absent 'fossa adjacent to mandibular condyle, foramen at base of pterygoid ramus',

115 Quadrate_posterior_pneumatic_recess / absent 'present as a lens-shaped fossa extending dorsally or dorsomedially from the quadrate foramen',

116 Dorsal_end_of_the_quadrate / with_a_single_head_that_fits_into_a_slot_on_the_ventral_side_of_the_squamosal 'double-headed, medial head contacts the braincase',

117 Quadrate_foramen / absent present,

118 Quadrate_foramen / developed_as_a_distinct_opening_between_the_quadrate_and_quadratojugal almost_entirely_closed_in_the_quadrate,

119 Ectopterygoid / 'slender, without ventral fossa' 'expanded, with a ventral depression medially' 'expanded, with a deep groove leading into the ectopterygoid body medially' deeply_excavated_and_medial_opening_constricted_into_a_foramen,

120 Dorsal_recess_on_ectopterygoid_ / absent present,

121 Ectopterygoid / posterior_to_palatine lateral_to_palatine,

122 Palatine_and_ectopterygoid / separated_by_pterygoid contact,

123 Contact_between_pterygoid_and_palatine / continuous 'discontinuous in the mid-region, resulting in a subsidiary palatal fenestra',

124 Flange_of_pterygoid_ / well_developed reduced_in_size_or_absent,

125 Shape_of_palatine_in_ventral_view / 'plate-like trapezoidal or subrectangular' tetraradiate jugal_process_strongly_reduced_or_absent,

126 Suborbital_fenestra_ / similar_in_length_to_orbit reduced_in_size_or_absent,

127 Infratemporal_fenestra / smaller_than_or_subequal_in_size_to_orbit 'strongly enlarged, more than 1.5 times the size of the orbit',

128 Postorbital_part_of_the_skull_roof / as_high_as_orbital_region deflected_ventrally_in_adult_individuals,

129 'Preorbital region of the skull in post-hatchling individuals' / 'elongate, nasals considerably longer than frontals, maxilla at least twice the length of the premaxilla' 'shortened, nasals subequal in length to frontals or shorter, maxillary length less than twice the length of the premaxillary body',

130 Occipital_region_of_the_skull_faces / posteriorly posteroventrally,

131 Basipterygoid_processes / 'well-developed, extending as a distinct process from the base of the basisphenoid' abbreviated_or_absent,

132 Basipterygoid_processes_well_developed_and / 'anteroposteriorly short and finger-like (approximately as long as wide)' longer_than_wide significantly_elongated_and_tapering,

133 Basipterygoid_processes / ventral_or_anteroventrally_projecting lateroventrally_projecting caudally_projecting,

134 Basipterygoid_processes / solid hollow,

135 Basipterygoid_recesses_on_dorsolateral_surfaces_of_basipterygoid_processes_ / absent present,

136 Basisphenoid_bulla / absent present,

137 Basisphenoid_recess / absent_or_poorly_developed 'deep and well-developed',

138 Passage_of_internal_carotids_between_posterior_end_of_skull_and_pituitary_fossa / no_bony_tubes_present enclosed_by_bony_tubes_extending_along_ventral_surface_of_basisphenoid,

139 Basisphenoid_recess_position / between_basisphenoid_and_basioccipital entirely_within_basisphenoid,

140 Posterior_opening_of_basisphenoid_recess_ / single 'divided into two small, circular foramina by a thin bar of bone',

141 Basisphenoid_between_basal_tubera_and_basipterygoid_processes / 'approximately as wide as long, or wider' 'significantly elongated, at least 1.5 times longer than wide',

142 Basisphenoid_in_lateral_view / oriented_subhorizontally 'anterior portion located much more ventrally than posterior portion, recess visible in posterior view',

143 Base_of_cultriform_process_ / not_highly_pneumatised 'expanded and pneumatic (parasphenoid bulba)',

144 Vestibular_and_Cochlear_branches_of_CN_VIII,

145 'Exits of CN X-XII' / flush_with_surface_of_exoccipital 'located together in a bowl-like basisphenoid depression',

146 Exits_of_CN_X_and_XI / laterally_through_the_jugular_foramen 'posteriorly through a foramen (metotic foramen) lateral to the exit of cranial nerve XII and the occipital condyle',

147 Exoccipital_lateral_to_occipital_condyle / forms_roof_over_exits_for_CN_X_and_XII unexpanded_and_does_not_form_roof,

148 Supraoccipital_sagittal_crest / with_pronounced_sagittal_crest sagittal_crest_reduced_or_absent,

149 Paroccipital_process_shape_ / elongate_and_slender 'short, deep ',

150 Paroccipital_process_direction / 'straight, projects laterally or posterolaterally' project_ventrolaterally pendant,

151 Paroccipital_process_dorsal_edge / with_straight_dorsal_edge 'distal end twists rostrally, distal ends of the processes oriented transversely rather than vertically',

152 Ventral_rim_of_the_basis_of_the_paroccipital_processes / above_or_level_with_the_dorsal_border_of_the_occipital_condyle 'situated at mid-height of occipital condyle or lower',

153 Foramen_magnum / 'subcircular, slightly wider than tall' 'oval, taller than wide',

154 Foramen_magnum_size / smaller_than_or_subequal_in_width_to_occipital_condyle larger_in_width_than_occipital_condyle,

155 Occipital_condyle / without_constricted_neck subspherical_with_constricted_neck,

156 Infracondylar_fossa_of_occipital_condyle / absent present,

157 Form_of_infracondylar_fossa_of_occipital_condyle / 'narrow and groove-like' 'broad depression approximately two-thirds the width of the occipital condyle',

158 Basal_tubera / present absent,

159 Basal_tubera_composition / equally_formed_by_basioccipital_and_basisphenoid_and_not_subdivided 'subdivided by a lateral longitudinal groove into a medial part entriely formed by the basioccipital, and a lateral part, entirely formed by the basisphenoid',

160 Basal_tubera_spacing / 'set far apart, level with or beyond lateral edge of occipital condyle and/or foramen magnum' 'tubera small, directly below condyle and foramen magnum, and separated by a narrow notch',

161 Subcondylar_recess / absent 'present in basioccipital/exoccipital lateral and ventral to occipital condyle',

162 Subcondylar_recess_form / 'isolated from nervous foramina CNX-CNXII' subcondylar_recess_and_cranial_nerves_exit_together_in_a_deep_depression_encompassing_multiple_pneumatic_fossae_and_enclosed_by_a_well_developed_rim,

163 'Exit of mid-cerebral vein' / included_in_trigeminal_foramen vein_exits_braincase_through_a_separate_foramen_anterodorsal_to_the_trigeminal_foramen,

164 Brain_proportions / forebrain_small_and_narrow forebrain_significantly_enlarged_and_triangular,

165 Anterior_tympanic_recess_in_the_braincase / absent present,

166 Prootic_pneumatic_recess / absent present,

167 Form_of_pneumatic_prootic_recess / 'dorsally open fossa on prootic/opisthotic' 'deep, posterolaterally directed concavity',

168 Crista_interfenestralis_ / confluent_with_lateral_surface_of_prootic_and_opisthotic distinctly_depressed_within_middle_ear_opening,

169 'Accessory dorsal tympanic recess (dorsal to crista interfenestralis) ' / absent present,

170 Form_of_dorsal_tympanic_recess / small_pocket_present extensive_with_indirect_pneumatisation,

171 'Caudal (posterior) tympanic recess ' / absent present,

172 Form_of_caudal_tympanic_recess / present_as_opening_on_anterior_surface_of_paroccipital_process 'extends into opisthotic posterodorsal to fenestra ovalis, confluent with this fenestra',

173 Exoccipitals_ventral_to_posterior_pneumatic_recess / no_lip 'form anteriorly projecting, posterodorsally curling, dorsally concave, tablike process',

174 Otosphenoidal_crest_ / 'vertical on basisphenoid and prootic, and does not border an enlarged pneumatic recess' 'well-developed, crescent-shaped, thin crest forms anterior edge of enlarged pneumatic recess',

175 'Subotic recess (pneumatic fossa ventral to fenestra ovalis) ' / absent present,

176 'Depression (possibly pneumatic) on ventral surface of postorbital process of laterosphenoid' / absent present,

177 Interorbital_region_in_adults / unossified ossified,

178 Prominent_endocranial_expansion_of_vertical_semicircular_canal / absent present,

179 Mandibular_foramen / absent_or_reduced large 'hypertrophied, greater than 50% dentary length',

180 Shape_of_mandibular_foramen / oval subdivided_by_a_spinous_rostral_process_of_the_surangular,

181 Paradental_plates_of_dentary / lack_paradental_plates with_paradental_plates_on_the_medial_surface_of_the_tooth_row,

182 Internal_mandibular_fenestra / 'small and slit-like' large_and_rounded,

183 Shape_of_anterior_end_of_dentary / blunt_and_unexpanded 'dorsoventrally expanded, rounded and slightly upturned' 'with anteroventral process giving a "squared off" appearance in lateral view',

184 Dorsal_edge_of_anterior_end_of_dentary_in_lateral_view / dorsally_flat 'with dorsally expanded, arcuate eminence',

185 Symphyseal_region_of_dentary / 'Broad and straight, paralleling lateral margin' medially_recurved,

186 Degree_of_medial_recurvature_of_dentary_symphysis / medially_recurved_slightly strongly_recurved_medially,

187 Dentary_symphyseal_fusion / absent present,

188 Dentary_anterior_end_in_lateral_view / in_line_with_main_part_of_buccal_edge anterior_end_deflected_ventrally,

189 Width_of_dentary_symphyseal_region / 'no broader than transverse width of post-symphyseal region' 'broader than post-symphyseal region',

190 Orientation_of_dentary_symphysis_in_lateral_view / vertical_to_subvertical 'projects strongly cranially, oblique with respect to dentary ventral margin',

191 Posterior_end_of_dentary / without_posterodorsal_process_dorsal_to_mandibular_fenestra with_dorsal_process,

192 Form_of_dentary_posterodorsal_process / developed_only_above_anterior_end_of_mandibular_fenestra with_elongate_dorsal_process_extending_over_most_of_fenestra,

193 Labial_face_of_dentary_ / flat with_lateral_ridge_and_inset_tooth_row,

194 Nutrient_foramina_on_external_surface_of_dentary_ / superficial descend_strongly_posteriorly_within_a_deep_groove,

195 Form_of_nutrient_foraminal_groove / thin_groove_of_constant_height_as_it_extends_posteriorly posterior_end_of_groove_is__dorsoventrally_expanded,

196 Dentary_shape_in_lateral_view / with_subparallel_dorsal_and_ventral_edges subtriangular_in_lateral_view,

197 Form_of_triangular_dentary / low_triangular high_triangular,

198 Ventral_edge_of_dentary_in_lateral_view / straight_or_nearly_straight descends_strongly_posteriorly,

199 Dentary_paradental_groove_separating_interdental_plates_from_medial_wall_of_dentary / absent present,

200 Pronounced_coronoid_eminence_on_the_surangular / absent present,

201 Foramen_in_lateral_surface_of_surangular_rostral_to_mandibular_articulation / absent present,

202 Number_of_surangular_foramina / one two,

203 Laterally_inclined_flange_along_dorsal_edge_of_surangular_for_articulation_with_lateral_process_of_lateral_quadrate_condyle / absent present,

204 Anterior_portion_of_the_surangular / less_than_half_the_height_of_the_mandible_above_the_mandibular_fenestra more_than_half_the_height_of_the_mandible_at_the_level_of_the_mandibular_fenestra,

205 Retroarticular_process_of_the_mandible / 'narrow, rod-like' 'broadened, with groove posteriorly for the attachment of the m. depressor mandibulae',

206 Attachment_of_the_m._depressor_mandibulae_on_retroarcticular_process_of_mandible / facing_dorsally facing_posterodorsally,

207 Retroarticular_process / points_posteriorly curves_gently_posterodorsally,

208 Articular_ / 'without elongate, slender medial, posteromedial, or mediodorsal process from retroarticular process' with_process,

209 Angular / 'exposed almost to end of mandible in lateral view, reaches or almost reaches articular' excluded_from_posterior_end_angular_suture_turns_ventrally_and_meets_ventral_border_of_mandible_rostral_to_glenoid,

210 Coronoid_ossification / absent present,

211 Form_of_coronoid_ossification / large thin_splint,

212 Splenial / not_widely_exposed_on_lateral_surface_of_mandible exposed_as_a_broad_triangle_between_dentary_and_angular_on_lateral_surface_of_mandible,

213 'Foramen in the ventral part of the splenial (mylohyal foramen)' / absent present,

214 Form_of_mylohyal_foramen / completely_enclosed_in_the_splenial opened_anteroventrally,

215 Posterior_end_of_splenial / straight forked,

216 Articular_glenoid_fossa / as_long_as_distal_end_of_quadrate 'twice or more as long as quadrate surface, allowing anteroposterior movement of mandible',

217 Palatal_teeth / present absent,

218 Premaxillary_teeth / present absent,

219 Number_of_premaxillary_teeth / three four five more_than_five,

220 First_premaxillary_tooth_size / slightly_smaller_or_the_same_size_as_2_and_3 much_smaller_than_2_and_3 much_larger_than_2_and_3,

221 Second_premaxillary_tooth / approximately_equivalent_in_size_to_other_premaxillary_teeth markedly_larger_than_third_and_fourth_premaxillary_teeth,

222 Premaxillary_tooth_direction / decumbent_or_ventrally_projecting procumbent,

223 Serrations_on_premaxillary_teeth / present absent,

224 'In cross section, premaxillary tooth crowns' / 'sub-oval to sub-circular' 'D-shaped with flat lingual surface',

225 Maxillary_teeth / present absent,

226 Length_of_maxillary_tooth_row / extends_posteriorly_to_approximately_half_the_length_of_the_orbit ends_at_the_anterior_rim_of_the_orbit 'completely antorbital, tooth row ends anterior to the vertical strut of the lacrimal' ends_below_the_junction_between_the_maxillary_body_and_the_ascending_process,

227 Number_of_maxillary_teeth / '10-14' '15-19' 20_or_more,

228 Maxillary_tooth_direction / ventrally_or_posteriorly_inclined procumbent,

229 'Maxillary and dentary teeth, mesial (anterior) carina' / present absent,

230 'Mesial (anterior) carina of maxillary and dentary teeth present and' / extends_to_base_of_crown 'terminates ventrally at approximately mid-crown level or more dorsally',

231 Shape_of_maxillary_teeth / 'mediolaterally flattened, dorsoventrally taller than anteropostiorly wide' 'lanceolate and subsymmetrical (as in therizinosaurs)' 'simple, conical, incisive crowns (as in Alvarezsaurs) ',

232 Degree_of_curvature_of_maxillary_tooth_crowns / crowns_curve_posteriorly_as_they_extend_distally very_little_curvature_or_crowns_straight,

233 Serrations_on_maxillary_and_dentary_teeth_ / present some_without_serrations_anteriorly absent,

234 Maxillary_tooth_implantation / separate_alveoli set_in_an_open_groove,

235 Roots_of_maxillary_and_dentary_teeth / mediolaterally_compressed 'circular in cross-section',

236 Dentary_tooth_row / fully_toothed only_teeth_rostrally edentulous fully_toothed_with_short_edentulous_anterior_portion,

237 Number_of_dentary_teeth / 'large, fewer than 25 in dentary' 'moderate number of small teeth (25-30 in dentary)' 'relatively small and numerous (more than 30 in dentary)',

238 Dentary_teeth_distribution / homodont 'increasing in size anteriorly, becoming more conical in shape' 'Decreasing in size anteriorly, becoming more densely packed',

239 Shape_of_dentary_teeth / 'mediolaterally flattened, dorsoventrally taller than anteroposteriorly wide' 'lanceolate and subsymmetrical (as in therizinosauroids)' 'simple, conical, incisive crowns (as in Alvarezsaurs) ',

240 Third_dentary_alveolus / subequal_in_size_to_other_alveoli circular_and_enlarged_relative_to_other_alveoli,

241 Dentary_tooth_implantation / separate_alveoli set_in_an_open_groove,

242 Dentary_tooth_direction / dorsally_or_posteriorly_inclined 'procumbent (anteriorly inclined)',

243 Serrations_on_maxillary_and_dentary_teeth / 'simple, denticles convex' 'distal and often mesial edges of teeth with large, hooked denticles that point toward the tip of the crown',

244 Serration_size / large small,

245 Constriction_between_tooth_crown_and_root / absent present,

246 Enamel_of_tooth_crowns / smooth 'horizontally wrinkled, especially flanking the serrations',

247 Form_of_enamel_wrinkles / bands_extending_across_labial_and_lingual_tooth_surfaces adjacent_to_carinae_but_do_not_extend_across_labial_and_lingual_tooth_surfaces,

248 Vertical_striations_of_enamel_of_tooth_crowns / absent present,

249 Axial_diapophyses / moderate reduced_or_absent,

250 Axial_parapophyses / prominent_or_moderate reduced_or_absent,

251 Axial_neural_spine / 'flared transversely and sheet-like' 'compressed mediolaterally, anteroposteriorly reduced, and rodlike',

252 Epipophyses_on_axis / absent present,

253 Form_of_axial_epipophyses / present_as_small_ridges 'strongly pronounced (overhanging the zygapophyses)',

254 Pleurocoel_in_axis / absent present,

255 Number_of_cervical_vertebrae / 10 More_than_10,

256 Pleurocoels_in_cervical_vertebrae / absent present,

257 Number / of pleurocoels in 'cervicals^n^n''' one two,

258 Arrangement_of_two_foramina_in_cortical_surface_of_cervical_centra / 'one in anterior half of lateral surface, one in posterior half' both_foramina_in_anterior_half,

259 Pleurocoels_developed_as / deep_depressions foramina,

260 Interior_pneumatic_spaces_in_cervicals / 'Structure camerate (few chambers)' 'Structure camellate (many chambers separated by delicate lamellae)',

261 Ventral_surface_of_anterior_cervicals / keeled smooth ventral_depression,

262 Posterolateral_crests_on_lateral_surfaces_of_cervical_centra / absent present,

263 Anterior_cervical_centra_length / less_than_twice_transverse_centrum_width between_two_and_three_time_transverse_width three_to_five_times_transverse_width,

264 Anterior_articular_facet_of_anterior_cervical_vertebrae / approximately_as_high_as_wide_or_higher significantly_wider_than_high 'wider than high and higher laterally than medially (kidney-shaped), with neural canal emarginating dorsal aspect',

265 Anterior_cervical_centra_relative_length_ / level_with_or_shorter_than_posterior_extent_of_neural_arch centra_extending_beyond_posterior_limit_of_neural_arch,

266 Articulation_surfaces_of_cervical_centra / 'amphi- to platycoelus' opisthocoelus heterocoelus,

267 Carotid_process_on_posterior_cervical_centra / absent present,

268 Epipophyses_in_anterior_cervical_vertebrae / absent_or_poorly_developed 'well-developed',

269 'Form of well-developed cervical epipophyses' / proximal_to_postzygapophyseal_facets strongly_overhanging_postzygapophyseal_facets,

270 'Prezygapophyseal-epipophyseal lamina on dorsal surface of neural arch' / absent_or_poorly_developed extending_anteriorly_from_epipophysis_as_a_mediolaterally_thin_ridge_that_separates_dorsal_surface_of_diapophysis_from_rest_of_dorsal_neural_arch,

271 'Postzygapophyses of cervical vertebrae 2-4' / 'well-separated, or connected only at the base' medially_connected_along_their_entire_length_by_a_intrazygapophyseal_lamina_that_is_dorsally_concave_for_attachment_of_the_interspinous_ligaments,

272 Cervical_neural_spines_ / anteroposteriorly_long 'anteroposteriorly short and centered on neural arch, giving arch an "X" shape in dorsal view' 'extremely short anteroposteriorly, less than 1/3 length of neural arch',

273 Cervical_neural_spine_height / 'dorsoventrally tall, subequal to or exceeding height of neural arch from centrum to base of neural spine' 'moderate, less than neural arch height' 'strongly reduced, less than half height of neural arch (not including spine itself)',

274 Prezygapophyses_in_anterior_cervicals / transverse_distance_between_prezygapophyses_less_than_width_of_neural_canal prezygapophyses_situated_lateral_to_the_neural_canal,

275 Prezygapophyses_in_anterior_postaxial_cervicals / straight 'anteroposteriorly convex, flexed ventrally anteriorly',

276 Pneumaticity_of_dorsal_neural_arches / absent_to_moderate extreme,

277 Hypapophyses_in_anterior_dorsals / absent_or_poorly_developed pronounced,

278 Pleurocoels_in_dorsal_vertebrae / absent 'present in anterior dorsals (''pectorals'')' present_in_all_dorsals,

279 Dorsal_centra_articular_surfaces / amphiplatyan opisthocoelus,

280 Ventral_keel_in_anterior_dorsals / absent_or_very_poorly_developed pronounced,

281 Shape_of_dorsal_centra_in_anterior_view / subcircular_or_oval significantly_wider_than_high triangular,

282 Posterior_dorsal_vertebrae / 'strongly shortened, centra much shorter than high' 'relatively short, centra approximately as high as long, or only slightly longer' 'significantly elongated, much longer than high',

283 'Posterior dorsal vertebrae, basal webbing of neural spines' / absent present,

284 'Posterior dorsal vertebrae, orientation of neural spines' / vertically_or_posteriorly anteriorly,

285 Anterior_dorsal_vertebrae_height_of_prezygadiapophyseal_lamina / less_than_or_subequal_to_height_of_centrum 'hypaxially inflated, height significantly greater than centrum height',

286 'Anterior dorsal vertebrae, anterior and posterior infrazygapophyseal fossae' / single with_one_or_more_accessory_centrodiapophyseal_laminae_dividing_fossa_into_multiple_chambers,

287 Transverse_processes_of_anterior_dorsal_vertebrae / subhorizontal_to_vertically_inclined pendant,

288 Parapophyseal_facets_of_anterior_dorsal_vertebrae / 'moderate in size (less than half height of centrum)' 'hypertrophied (greater than two thirds centrum height)',

289 'Hyposphene-hypantrum articulation in dorsal vertebrae' / absent present,

290 'Step-like ridge lateral to hyposphene running posterodorsally from the dorsal border of the neural canal to the posterior edge of the postzgyapophyses of dorsal vertebrae (visible in lateral view)' / absent present,

291 Postzygapophyses_of_the_dorsal_vertebrae_in_posterior_view / without_lateral_flanges 'with lateral, small, flange-like lateral extensions of postzygapophyseal facets',

292 Postzygapophyses_of_dorsal_vertebrae / 'abutting one another above neural canal, opposite hyposphenes meet to form lamina' 'zygapophyses placed lateral to neural canal and separated by groove for interspinous ligaments, hyposphenes separated',

293 Neural_spines_on_posterior / 'rectangular_or_square' '/' 'anteroposteriorly expanded distally, fan-shaped',

294 Neural_spines_of_dorsal_vertebrae_in_dorsal_view / not_expanded_distally 'expanded laterally in dorsal view to form "spine table"',

295 Scars_for_interspinous_ligaments / terminate_at_apex_of_neural_spine_in_dorsal_vertebrae terminate_below_apex_of_neural_spine,

296 Neural_spines_of_posterior_dorsals / broadly_rectangular_and_approximately_as_dorsoventrally_high_as_anteroposteriorly_long 'high rectangular, significantly dorsoventrally higher than anteroposteriorly long',

297 'Hook-like extension on anterior end of dorsal neural spines in lateral view' / absent 'present (with associated depression immediately caudal to the projection for spinous ligament attachment)',

298 Parapophyses_of_posterior_dorsal_vertebrae / flush_with_neural_arch distinctly_projected_on_pedicels,

299 Parapophyses_in_posteriormost_dorsals / on_same_level_as_transverse_process distinctly_below_transverse_process,

300 Transverse_processes_of_anterior_dorsal_vertebrae / proximodistally_long_and_anteroposteriorly_thin 'proximodistally short, anteroposteriorly wide',

301 Notarium_of_dorsal_vertebrae / absent present,

302 Number_of_sacral_vertebrae / two three four five six seven eight nine_or_more,

303 Pleurocoels_in_centra_of_sacral_vertebrae / absent present_on_anterior_sacrals_only present_on_all_sacrals,

304 Ventral_surface_of_posterior_sacral_centra / 'gently rounded, convex' 'flattened ventrally, sometimes with shallow sulcus' 'centrum strongly constricted transversely, ventral surface keeled',

305 'Transverse dimensions of mid-sacral centra relative to other sacral centra' / subequal mediolaterally_narrower mediolaterally_wider,

306 Sacral_vertebrae / with_unfused_zygapophyses with_fused_zygapophyses_forming_a_sinuous_ridge_in_dorsal_view,

307 Last_sacral_centrum_ / with_flat_posterior_articulation_surface convex_articulation_surface,

308 Fenestrae_between_neural_spines_of_sacral_vertebrae / present absent,

309 Sacral_ribs / 'slender and well-separated' forming_a_more_or_less_continuous_sheet_in_ventral_or_dorsal_view very_massive_and_strongly_expanded,

310 Sacral_neural_arch_pneumaticity / absent_to_moderate extreme,

311 Number_of_caudal_vertebrae / more_than_40 '25-40' fewer_than_25,

312 Pygostyle / absent 'present, centra of distal caudal vertebrae fused',

313 Pleurocoels_in_centra_of_anterior_caudal_vertebrae / absent present,

314 Caudal_centra / amphiplatyan procoelus,

315 Shape_of_anterior_caudal_centra / oval 'subrectangular and box-like' laterally_compressed_with_a_ventral_keel,

316 Ventral_surface_of_anterior_caudals / rounded 'with a distinct keel sometimes bearing a narrow, shallow groove on its midline' grooved,

317 Relative_length_of_distal_caudal_centra / significantly_elongated_in_relation_to_centrum_height not_elongated_in_relation_to_centrum_height,

318 Caudal_vertebrae / 'with distinct transition point, from shorter centra with long transverse processes proximally to longer centra with small or no transverse processes distally' 'homogeneous in shape, with no transition point',

319 Position_of_transition_point / distal_to_the_tenth_caudal_vertebra between_the_7th_and_10th_caudal_vertebrae proximal_to_the_7th_caudal_vertebra,

320 Location_of_transverse_processes_of_proximal_caudals / centrally_positioned_on_centrum anteriorly_displaced,

321 Centrodiapophyseal_laminae_of_anterior_caudal_vertebrae / weak 'prominent, as well developed as those of dorsal vertebrae',

322 Neural_spines_on_distal_caudals / form_a_low_ridge spine_absent midline_sulcus_in_center_of_the_neural_arch,

323 Neural_spines_of_caudal_vertebrae / 'simple, undivided' separated_into_anterior_and_posterior_alae_throughout_much_of_caudal_sequence,

324 'Neural spines of mid-caudals' / 'rod-like and posteriorly inclined' 'rod-like and vertical' 'subrectangular and sheet-like',

325 Prezygapophyses_of_distal_caudal_vertebrae / 'between 1/3 and whole centrum length' 'with extremely long extensions of the prezygapophyses (up to 10 vertebral segments in some taxa)' strongly_reduced_as_in_Archaeopteryx_lithographica,

326 'Anterior margin of neural spines of anterior mid-caudal vertebrae' / straight 'with distinct kink, dorsal part of anterior margin more strongly inclined posteriorly than ventral part',

327 'Long, hair-like cervical ribs' / absent present,

328 Shaft_of_cervical_ribs / slender_and_longer_than_vertebra_to_which_they_articulate broad_and_shorter_than_vertebra,

329 Posterior_cervical_ribs_and_centra / separate fused,

330 Ossified_uncinate_processes / absent present,

331 Ossified_sternal_ribs / absent present,

332 Lateral_gastral_segment / shorter_than_medial_one_in_each_arch distal_segment_longer_than_proximal_segment,

333 Cranial_process_at_base_of_chevrons / absent present,

334 Proximal_surface_of_chevrons / distinct_transverse_ridge_dividing_surface_into_anterior_and_posterior_facets 'no ridge, low mounds may be present laterally',

335 Proximal_end_of_chevrons_of_proximal_caudals_ / 'short anteroposteriorly, shaft cylindrical' 'proximal end elongate anteroposteriorly, flattened and plate-like',

336 'Mid-caudal chevrons' / 'rod-like or only slightly expanded ventrally' 'L-shaped',

337 Distal_chevrons / 'rod-like or L-shaped' 'skid-like',

338 Distal_caudal_chevrons_ / simple anteriorly_bifurcate bifurcate_at_both_ends,

339 Ossified_sternal_plates / separate_in_adults fused,

340 Ventral_keel_on_sternum / absent present,

341 Sternum / without_distinct_lateral_xiphoid_process_posterior_to_costal_margin with_lateral_xiphoid_process,

342 Furcula / absent present,

343 Furcula_shape / 'v-shaped' 'u-shaped, with bowed epicleidea',

344 Hypocleidium_on_furcula / absent present,

345 'Articular facet of coracoid on sternum (conditions may be determined by the articular facet on coracoid in taxa without ossified sternum' / anterolateral_or_more_lateral_than_anterior almost_anterior,

346 Anterior_edge_of_sternum / grooved_for_reception_of_coracoids without_grooves,

347 Coracoid_in_lateral_view_ / 'subcircular, with low ventral blade and no or small posterior process' shallow_ventral_blade_with_elongate_posterior_process subquadrangular_with_extensive_ventral_blade 'strut-like, very tall ventral blade with little or no posterior process',

348 Posterior_edge_of_coracoid / not_or_shallowly_indented_below_glenoid 'posterior edge of coracoid deeply notched just ventral to glenoid, glenoid lip everted',

349 External_surface_of_coracoid_ventral_to_glenoid_fossa_and_along_dorsal_margin_of_posterventral_blade / unexpanded 'expanded, forms triangular subglenoid fossa bounded laterally by coracoid tuber',

350 Coracoid_tubercle / absent present,

351 Coracoid_tubercle_form / 'anteroposteriorly short, mound-like' 'anteroposteriorly elongated, ridge-like',

352 Coracoid_foramen / present absent,

353 Scapula_shape / 'short and broad (ratio length/minimal height of shaft <9)' 'slender and elongate (ratio >10)',

354 Scapulocoracoid_junction_anterior_surface / indented_or_notched_between_the_scapular_acromial_process_and_the_coracoid_suture smoothly_curved_and_uninterrupted_across_the_contact_between_the_scapula_and_coracoid,

355 Acromion_margin_of_scapula / continuous_with_blade anterior_edge_enlarged_and_projects_anteriorly_at_approximately_a_right_angle,

356 'Flange on supraglenoid buttress on scapula (see Nicholls and Russell, 1985) ' / absent present,

357 Distal_end_of_scapula / expanded not_expanded,

358 Glenoid_fossa_ / faces_posteriorly_or_posterolaterally faces_laterally,

359 Scapula_and_coracoid_ / separate fused_into_scapulocoracoid,

360 Scapula_and_coracoid_orientation / continuous_arc_in_posterior_and_anterior_views 'coracoid inflected medially, scapulocoracoid L shaped in lateral view',

361 Scapula_length / longer_than_humerus shorter_than_humerus,

362 Deltopectoral_crest_length / less_than_one_quarter_humeral_length approximately_one_third_humeral_length greater_than_one_half_humeral_length,

363 Deltopectoral_crest / 'large and distinct, proximal end of humerus quadrangular in anterior view' 'less pronounced, forming an arc rather than being quadrangular' 'very weakly developed, proximal end of humerus with rounded edges' 'extremely long (as in Shuvuuia and Mononykus)',

364 Deltopectoral_crest_orientation / longitudinal oblique_distolaterally_and_distal_end_of_crest_oriented_laterally_rather_than_anteriorly_from_the_humeral_shaft,

365 Lateral_surface_of_distal_end_of_deltopectoral_crest / smooth with_distinct_muscle_scar_near_lateral_edge_along_distal_end_of_crest_for_insertion_of_biceps_muscle,

366 'Ratio femur/humerus' / more_than_2.5 between_1.2_and_2.2 less_than_1,

367 Outline_of_proximal_articular_facet_of_humerus / 'broadly oval (more than twice as broad transversely than anteroposteriorly)' 'distinctly rounded, often globular (less than twice as broad anteroposteriorly than transversely)',

368 Internal_tuberosity_of_humerus / small_and_confluent_with_humeral_head 'offset from humeral head by distinct notch, often projects proximally above humeral head' 'hypertrophied but not distinct from humeral head (as in Suchomimus)',

369 Shape_of_internal_tuberosity_on_humerus_in_anterior_view / 'triangular, often rounded' rectangular,

370 Humerus_in_lateral_view / sigmoidal straight,

371 Transverse_width_of_distal_humerus / greater_than_2.7_times_shaft_width between_2_and_2.5_times_humeral_shaft_width less_than_twice_shaft_width,

372 'Ectepicondyle of humerus (lateral epicondyle)' / 'small, often rectangular and does not form articular surface' 'large, rounded and forms articular surface',

373 'Entepicondyle of humerus (medial epicondyle)' / absent_or_small_and_tabular 'large, projects medially from ulnar condyle as a distinct process and is distally separated from ulnar condyle by a groove',

374 Distal_humeral_condyles / 'primarily developed on distal end of humerus, but may also have some articular surface extending to anterior edge' 'limited to anterior surface, condylar surfaces not present on distal end',

375 Ulnar_shaft / straight bowed,

376 Olecranon_process_of_ulna / absent_or_weakly_developed 'well-developed' hypertrophied,

377 Shape_of_olecranon_process / transversely_broad 'mediolaterally thin, blade-like',

378 Crest_extending_along_posterior_surface_of_ulnar_shaft_from_olecranon_process / absent present,

379 Proximal_surface_of_ulna_ / single_continuous_articular_facet divided_into_two_distinct_fossae,

380 Proximal_end_of_the_ulna_in_proximal_view / without_extensive_coronoid_process_and_radial_process_on_radial_side_of_proximal_end coronoid_and_medial_processes_large,

381 Distal_articular_surface_of_ulna_ / flat 'convex, semilunate surface',

382 Distal_condyle_articular_surface_of_ulna / 'unexpanded or spatulate, articular surface limited to distal end' 'bulbous, trochlear articular surface extends onto dorsal surface of ulna',

383 Radius_length / more_than_half_the_length_of_humerus less_than_half_the_length_of_humerus,

384 Radial_shaft / straight bowed_laterally,

385 Radius_and_ulna / 'well-separated' with_distinct_adherence_or_syndesmosis_distally,

386 Ossified_carpals / absent present,

387 'Lateral proximal carpal (ulnare?) ' / quadrangular triangular_in_proximal_view,

388 Trochlea_on_the_proximal_surface_of_distal_carpal_1 / absent present,

389 Two_distal_carpals_ / 'in contact with metacarpals, one covering the base of Mc I (and perhaps contacting Mc II) , the other covering the base of Mc II' 'two distal carpals not present, single distal carpal capping Mc I and II',

390 Distal_carpals_ / not_fused_to_metacarpals 'fused to metacarpals, forming carpometacarpus',

391 Rectangular_buttress_on_ventrolateral_surface_of_proximal_end_of_Mc_I / absent present,

392 Length_of_Mc_I / approximately_half_the_length_of_Mc_II subequal_in_length_to_Mc_II,

393 Shape_of_Mc_I / significantly_longer_than_broad 'very stout, approximately as long as broad',

394 Contact_between_Mc_I_and_Mc_II / metacarpals_contact_each_other_at_their_bases_only 'Mc I closely appressed to Mc II, at least the proximal half of McI flattened',

395 'Medial tab on proximal end of Mc I (''proximo-radial process of Gishlick and Gauthier,2007)' / absent_or_poorly_developed 'well-developed, extending far medially',

396 Distal_end_of_Mc_I / condyles_more_or_less_symmetrical 'condyles strongly asymmetrical, the medial condyle being positioned more proximally than the lateral',

397 Distal_articular_end_of_metacarpal_I / ginglymoid rounded_and_smooth,

398 Medial_side_of_Mc_II / expanded_proximally not_expanded,

399 Distal_articular_end_of_McII / ginglymoid without_ginglymus,

400 Shaft_of_Mc_III / subequal_in_width_to_Mc_II 'considerably more slender than Mc II (less than 70% of the width of Mc II)',

401 Proximal_articular_end_of_Mc_III / expanded_and_similar_in_width_to_Mc_I_and_II 'not expanded, very slender when compared to Mc I and II',

402 Proximal_outline_of_Mc_III / subrectangular 'triangular, apex dorsal',

403 Shaft_of_Mc_III / straight bowed_laterally,

404 Extensor_pits_on_the_dorsal_surface_of_the_distal_end_of_metacarpals / absent_or_poorly_developed 'deep, well-developed',

405 Number_of_manual_digits_with_one_or_more_phalanges / five four three two,

406 Number_of_metacarpals / five four three,

407 Paired_flexor_processes_on_proximal_ventral_surfaces_of_proximalmost_phalanges / absent present,

408 'Flexor surface of manual phalanx I-1' / convex_or_flat 'concave, ''axial furrow'' along proximodistal axis',

409 'Shaft diameter of phalanx I-1' / less_than_shaft_diameter_of_radius greater_than_shaft_diameter_of_radius,

410 'Proximodistal length of phalanx I-1/length of Mc I' / 1_or_less between_1_and_1.5 more_than_1.5,

411 Penultimate_phalanx_of_the_second_finger / shorter_than_first_phalanx longer_than_first_phalanx,

412 Penultimate_phalanx_of_the_third_finger / 'as long as, or shorter than, more proximal phalanges' longer_than_each_of_the_more_proximal_phalanges longer_than_both_proximal_phalanges_taken_together,

413 Length_of_third_manual_digit / longer_than_second_finger shorter_than_or_equal_in_length_to_second_finger,

414 'Proximal articular surface of manual ungual I-2' / dorsoventrally_much_taller_than_mediolaterally_wide mediolaterally_as_broad_as_tall,

415 Unguals_on_all_manual_digits / generally_similar_in_size digit_I_bearing_large_ungual_and_unguals_of_other_digits_distinctly_smaller,

416 Transverse_ridge_immediately_dorsal_to_the_articulating_surface_of_unguals / absent present,

417 Flexor_tubercle_placement / proximal distal absent,

418 Curvature_of_ventral_surface_manual_ungual_I / strongly_curved weakly_curved straight,

419 Curvature_of_ventral_surface_of_manual_unguals_II_and_III / strongly_curved weakly_curved_ straight,

420 Flexor_tubercle_size / 'large (> 1/3 articular facet height)' 'small (< 1/3 articular facet height)',

421 'Lateral grooves of manual ungual I-2 in ventral view' / unenclosed proximal_end_of_grooves_partially_enclosed_by_lateral_notches proximal_end_of_grooves_passes_through_foramena_on_ventral_surface_of_ungual,

422 Fusion_of_pelvic_elements_in_adults / absent present,

423 Ilium / brachyiliac dolichoiliac,

424 Ilium_pneumaticity / little_or_none large_external_pneumatic_foramina_and_internal_spaces,

425 Dorsal_margin_of_ilium / subhorizontal_or_gently_inclined_relative_to_axis_of_pubic_and_ischial_contact 'rises steeply as it extends anteriorly, at least 30 degree angle from the axis of the pubic and ischial contact',

426 Ventral_edge_of_anterior_ala_of_ilium_ / straight_or_gently_curved ventral_edge_hooked_anteriorly,

427 Form_of_hook_of_preacetabular_ala_of_ilium / weak strong,

428 Preacetabular_part_of_ilium / significantly_shorter_than_postacetabular_part subequal_in_length_to_postacetabular_part significantly_longer_than_postacetabular_process,

429 Anterior_rim_of_ilium / shallowly_convex_or_straight strongly_convex_or_pointed_anteriorly,

430 'Dorsally-positioned, anteriorly-concave notch on anterior rim of ilium' / absent present,

431 'Preacetabular part of ilium (height)' / 'approximately as high as postacetabular part (excluding the ventral expansion)' significantly_higher_than_postacetabular_part significantly_lower_than_the_postacetabular_part,

432 Cuppedicus_fossa / absent present,

433 Form_of_cuppedicus_fossa / 'deep, ventrally concave' 'fossa shallow or flat, with no lateral overhang',

434 Cuppedicus_fossa_position / ridge_bounding_fossa_terminates_rostral_to_acetabulum_or_curves_ventrally_onto_anterior_end_of_pubic_peduncle rim_extends_far_posteriorly_and_is_confluent_or_almost_confluent_with_acetabular_rim,

435 Preacetabular_portion_of_ilium / parasagittal moderately_laterally_flaring,

436 Brevis_fossa_shape / 'shelf-like, narrow with subparallel margins' 'deeply concave, expanded posteriorly with lateral overhang',

437 Brevis_fossa_lateral_view / 'Poorly developed adjacent to ischial peduncle, without lateral overhang and medial edge of the brevis fossa is visible' 'well developed fossa along full length of postacetabular blade, lateral overhang extends along full length of fossa, medial edge of brevis fossa covered in lateral view',

438 Medial_brevis_shelf / 'strongly developed, projects medially' low_ridge_on_medial_surface_of_postacetabular_ala,

439 Shape_of_postacetabular_ala_of_ilium_in_lateral_view / squared acuminate,

440 Postacetabular_ala_of_ilium_in_lateral_view / ventral_edge_flat ventral_edge_concave ventral_edge_concave_and_distal_end_extends_ventrally_below_level_of_the_ventral_margin_of_the_ischial_peduncle,

441 Articulation_of_iliac_blades_with_sacrum / 'vertical, well-separated above sacrum' 'strongly inclined mediodorsally, almost contacting each other or sacral neural spines at midline',

442 Vertical_ridge_on_iliac_blade_above_acetabulum / absent low_ridge_with_associated_foramina 'well-developed',

443 Shape_of_pubic_peduncle_of_ilium / transversely_broad_and_roughly_triangular_in_outline anteroposteriorly_elongated_and_narrow,

444 Iliac_pubic_peduncle_length_relative_to_iliac_ischial_peduncle / 'significantly longer than ischial peduncle, ischial peduncle tapering ventrally and without clearly defined articular facet' subequal_in_length_to_ischial_peduncle anteroposteriorly_shorter_than_the_ischial_peduncle,

445 Articulation_facet_of_pubic_peduncle_of_ilium / 'facing more ventrally than anteriorly, and without a pronounced kink' with_pronounced_kink_and_anterior_part_facing_almost_entirely_anteriorly,

446 Anterior_margin_of_pubic_peduncle / straight_or_convex concave,

447 Supraacetabular_crest / absent_ present_,

448 Form_of_supraacetabular_crest / forms_hood_over_femoral_head 'reduced, not forming hood',

449 Antitrochanter_posterior_to_acetabulum_ / absent_or_poorly_developed prominent,

450 Postacetabular_blades_of_ilia_in_dorsal_view_ / parallel diverge_posteriorly,

451 'Tuber along dorsal edge of ilium, dorsal or slightly posterior to acetabulum ' / absent present,

452 Dorsal_margin_of_postacetabular_ala_in_lateral_view / convex_or_straight 'concave, brevis shelf extends caudal to lateral ilium making it appear concave in lateral view',

453 Caudal_end_of_postacetabular_ala_in_dorsal_view / rounded_or_squared_in_dorsal_view 'lobate, with brevis shelf extending caudally beyond caudal terminus of the postacetabular ala',

454 Ilium_and_ischium_articulation / 'flat or slightly concavo-convex' with_process_projecting_into_socket_in_ischium,

455 Pubic_orientation / propubic vertical moderately_posteriorly_oriented opisthopubic,

456 Strongly_expanded_pubic_boot / absent present,

457 Pubic_boot_projects / anteriorly_and_posteriorly with_little_or_no_anterior_process only_expanded_anteriorly,

458 Ratio_length_of_pubic_boot_to_length_of_pubic_shaft / less_than_0.3 more_than_0.5,

459 'Pubic boot outline, distal view' / triangular 'narrow, with subparallel margins',

460 Pubic_apron / present absent,

461 Form_of_pubic_apron / extends_medially_from_middle_of_cylindrical_pubic_shaft shelf_extends_medially_from_anterior_edge_of_anteroposteriorly_flattened_shaft,

462 Pubic_apron / about_half_of_pubic_shaft_length 'less than 1/3 of shaft length',

463 Pubic_apron / completely_closed with_medial_opening_distally_above_the_pubic_boot,

464 Pubic_obturator_foramen / present absent,

465 Form_of_pubic_obturator_foramen / completely_enclosed 'open ventrally (obturator notch)',

466 Pubic_fenestra_below_obturator_foramen / absent present,

467 Pubic_shafts_in_lateral_view / straight anteriorly_convex anteriorly_concave,

468 Lateral_face_of_pubic_shafts / smooth with_prominent_lateral_tubercle_about_halfway_down_the_shaft,

469 Length / of 'Ischium^n^n''' 'more than two-thirds pubis length' two_thirds_or_less_of_pubic_length,

470 Obturator_process_of_ischium_ / absent present,

471 Position_of_obturator_process / proximal_in_position located_near_middle_of_ischiadic_shaft located_at_distal_end_of_ischium,

472 Ischial_shaft / Rodlike anteroposteriorly_wide_and_plate_like,

473 Lateral_blade_of_ischium / flat_or_laterally_convex laterally_concave 'with longitudinal ridge subdividing lateral surface into anterior (including obturator process) and posterior parts',

474 'Ischium, lateral view' / straight distally_curved_anteriorly distally_curved_posteriorly,

475 'Ischium, anterior view' / straight laterally_convex twisted_at_midshaft_and_with_flexure_of_obturator_process_toward_midline_so_that_distal_end_is_horizontal laterally_concave,

476 Contact_of_obturator_process_of_ischium / does_not_contact_pubis contacts_pubis,

477 Ventral_notch_at_distal_edge_of_ischial_obturator_process_ / 'absent, grades smoothly into ischial shafts' present,

478 Obturator_process_on_ischium / confluent_with_pubic_peduncle offset_from_pubic_peduncle_by_a_distinct_notch,

479 Morphology_of_offset_triangular_obturator_process_of_ischium / 'wide base along ischiac shaft, rostral process short' 'narrow base, rostral process elongate',

480 Distal_end_of_ischium / 'strongly expanded, forming ischial "boot"' slightly_expanded tapering,

481 Distal_ends_of_ischia_ / form_symphysis approach_one_another_but_do_not_form_symphysis widely_separated,

482 Distally_placed_process_on_caudal_margin_of_ischium / absent present,

483 Tubercle_on_anterior_edge_of_ischium / absent present,

484 'Posterior process (ischial tuberosity) on posteroproximal part of ischium' / absent_ 'well-developed',

485 'Form of posteroproximal ischial process (ischial tuberosity)' / 'small, tablike' 'large, proximodorsally hooked and separated from the iliac peduncle by a notch',

486 Semicircular_scar_on_posterior_part_of_the_proximal_end_of_the_ischium / absent present,

487 Femoral_length / longer_than_tibia shorter_than_tibia,

488 Femoral_head / without_fovea_capitalis circular_fovea_present_in_center_of_medial_surface_of_head,

489 Oblique_ligament_groove_on_the_posterior_surface_of_femoral_head / absent_or_very_shallow 'deep, bound medially by a well-developed posterior lip',

490 Femoral_head_and_greater_trochanter / confluent_with_greater_trochanter separated_from_greater_trochanter__by_a_distinct_cleft,

491 Femoral_head_direction_anteroposterior / directed_anteromedially directed_strictly_medially,

492 Femoral_head_direction_dorsoventral / ventromedial horizontal dorsomedial,

493 Greater_trochanter / anteroposteriorly_narrow_and_narrowing_from_medial_to_lateral 'anteroposteriorly expanded, forming a trochanteric crest',

494 Lesser_trochanter / separated_from_greater_trochanter_by_a_deep_cleft trochanters_separated_by_small_groove 'completely fused (or absent) to form crista trochanteris',

495 Lesser_trochanter_shape / alariform cylindrical_in_cross_section 'very short and ridge-like',

496 Proximal / extent of lesser 'trochanter^n^n''' at_distal_end_of_femoral_head 'more proximally placed, but distal to greater trochanter' as_proximal_or_more_proximal_than_greater_trochanter,

497 Accessory_trochanteric_crest_on_distal_end_of_lesser_trochanter / absent present,

498 Posterolateral_trochanter / absent_or_represented_only_by_rugose_area 'posterior trochanter distinctly raised from shaft, mound-like',

499 Fourth_trochanter_on_femur_ / present absent,

500 Broad_groove_on_extensor_surface_of_distal_femur / absent_or_poorly_developed well_developed,

501 'Femoral medial epicondyle (medial distal crest, expanded medial lamella)' / stout_ridge_or_absent 'flange like, medially extensive',

502 Popliteal_fossa_on_distal_end_of_femur / open_distally closed_off_distally_by_contact_between_distal_condyles,

503 Infrapopliteal_ridge_present_posteriorly_between_medial_condyle_and_crista_tibiofibularis / absent present,

504 Distal_end_of_femur / anteroposteriorly_broad_and_distally_flattened less_broad_and_well_rounded,

505 Lateral_femoral_distal_condyle / distally_rounded distally_conical,

506 Distal_projection_of_lateral_femoral_distal_condyle / approximately_the_same_level_as_the_medial_condyle distinctly_further_than_medial_condyle_and_distal_surface_of_medial_condyle_is_flattened,

507 Anteroposterior_length_of_proximal_end_of_tibia_in_proximal_view / exceeds_mediolateral_width less_than_mediolateral_width,

508 Cnemial_crest_proximal_projection / approximately_at_the_same_level_as_posterior_condyles projects_strongly_proximal_to_posterior_condyles,

509 Anteroposterior_length_of_cnemial_crest / prominent_but_not_expanded anteroposteriorly_expanded,

510 Accessory_ridge_on_lateral_surface_cnemial_crest / absent present,

511 'Medial cnemial crest and lateral cnemial crest(also called the cranial cnemial crest in birds)' / absent present,

512 Fibular_condyle_on_proximal_end_of_tibia / confluent_with_cnemial_crest_anteriorly_in_proximal_view strongly_offset_from_cnemial_crest,

513 Medial_proximal_condyle_on_tibia / round_in_proximal_view arcuate_and_posteriorly_angular_in_proximal_view,

514 Posterior_cleft_between_medial_part_of_the_proximal_end_of_the_tibia_and_fibular_condyle / absent present,

515 'Fibular crest (ridge on lateral side of tibia for connection with fibula)' / absent present,

516 Form_of_fibular_crest / extending_from_proximal_articular_surface_distally clearly_separated_from_proximal_articular_surface,

517 Shape_of_fibular_crest / quadrangular low_and_rounded_,

518 Fibular_crest_distal_extension / proximally_positioned extends_to_midshaft_of_tibia,

519 Fibular_crest_length / 'short, less than one fifth tibial length' 'long, between one quarter and one third tibial length',

520 Bracing_for_ascending_process_of_astragalus_on_anterior_side_of_distal_tibia / 'distinct ''step'' running obliquely from mediodistal to lateroproximal' anterior_side_of_tibia_flat 'Step-like ridge running proximodistally rather than obliquely',

521 Fibula / reaches_proximal_tarsals 'short, tapering distally, and not in contact with proximal tarsals ',

522 Lateral_surface_of_proximal_fibula / shallow_longitudinal_trough_situated_posteriorly 'trough absent or weak groove present, surface convex',

523 Proximal_fibular_margin / subhorizontal cranial_portion_extends_proximally_beyond_level_of_posterior_portion,

524 Fibular_proximal_dimensions_in_proximal_view / anterior_portion_subequal_to_posterior_portion_in_mediolateral_width anterior_portion_mediolaterally_wider_than_posterior_portion,

525 Insertion_of_m._iliofibularis_on_fibular_shaft / not_especially_marked 'present as a well-developed anterolateral tubercle',

526 Position_of_insertion_of_m._iliofibularis_on_fibular_shaft / proximal midshaft,

527 'Ridge on medial side of proximal end of fibula, that runs anterodistally from the posterproximal end' / absent present,

528 Medial_surface_of_proximal_end_of_fibula / concave_along_long_axis flat,

529 Deep_oval_fossa_on_medial_surface_of_fibula_near_proximal_end / absent present,

530 Astragalus_and_Calcaneum / condyles_indistinct_or_poorly_separated distinct_condyles_separated_by_prominent_vertical_tendinal_groove_on_anterior_surface,

531 Astragalus_and_calcaneum / separate_from_tibia fused_to_each_other_and_to_the_tibia_in_late_ontogeny,

532 Fibular_facet_on_astragalus / large_and_facing_partially_proximally reduced_and_facing_laterally_or_absent,

533 Height_of_ascending_process_of_the_astragalus / lower_than_astragalar_body higher_than_astragalar_body more_than_twice_the_height_of_astragalar_body,

534 Shape_of_ascending_process_of_the_astragalus / 'broad, covering most of anterior surface of distal end of tibia' 'narrow, covering only lateral half of anterior surface of tibia',

535 Notch_on_medial_edge_of_ascending_process_of_the_astragalus / absent present,

536 'Fossa on anterior surface of mesial base of ascending process of astragalus, sometimes bearing accessory fenestrations' / absent present,

537 Ascending_process_of_astragalus_and_astragalar_body / confluent_or_only_slightly_offset_from_astragalar_body offset_from_astragalar_body_by_a_pronounced_groove,

538 Astragalar_condyles / almost_entirely_below_tibia_and_face_distally significantly_expanded_proximally_on_anterior_side_of_tibia_and_face_anterodistally,

539 Horizontal_groove_across_astragalar_condyles_anteriorly / absent present,

540 Calcaneum / without_facet_for_tibia 'well-developed facet for tibia present',

541 Distal_tarsals / 'separate, not fused to metatarsals' form_metatarsal_cap_with_intercondylar_prominence_that_fuses_to_metatarsal_early_in_postnatal_ontogeny,

542 Metatarsals_coossification / 'not co-ossified' coossified,

543 'Shafts of metatarsals II-IV' / not_closely_appressed_beyond_proximal_half_of_metatarsus 'closely appressed throughout most of metatarsus, adjacent surfaces flattened for contact',

544 Maximum_length_of_metatarsals / greater_than_50%_tibia_length less_than_50%_tibia_length,

545 Metatarsal_I / present absent,

546 Metatarsal_I / 'attenuates proximally ,without proximal articulating surface' 'proximal end of Mt I similar to that of Mt II-IV',

547 Metatarsal_I / contacts_the_ankle_joint does_not_contact_the_ankle_joint,

548 'Position of distally-placed Mt I' / 'reduced, elongated and splint-like, articulates in the middle of the medial surface of Mt II' broadly_triangular_and_attached_to_the_distal_quarter_of_Mt_II,

549 Metatarsal_II_proximal_end_of_flexor_surface / flat_or_small_tab_present large_quadrangular_flange_present,

550 Distal_end_of_metatarsal_II / 'smooth, not ginglymoid' with_developed_ginglymus,

551 Tuber_along_extensor_surface_of_MtII / absent present,

552 Posteromedial_margin_MtII_diaphysis / 'well-developed flange absent or area rugose' with_flange_projecting_caudally_or_medially,

553 Distal_end_of_metatarsal_III / 'smooth, not ginglymoid' with_developed_ginglymus,

554 Metatarsal_III / subequal_in_width_to_Mt_II_and_IV_proximally pinched_between_II_and_IV_and_not_visible_in_anterior_view_proximally does_not_reach_the_proximal_end_of_the_metatarsus mediolaterally_much_wider_than_either_II_or_IV,

555 Metatarsal_III_shape_of_proximal_end / 'rectangular, medial and lateral surfaces pinched' 'hourglass-shaped, medial and or lateral surface(s) concave',

556 Medial_side_of_anterior_surface_of_distal_end_of_MtIII / unexpanded expanded,

557 Metatarsal_III_shape_of_shaft_in_cross_section / rectangular 'wedge-shaped, plantar surface pinched',

558 Shaft_of_MT_IV / round_or_thicker_dorsoventrally_than_wide_in_cross_section shaft_of_Mt_IV_mediolaterally_widened_and_flat_in_cross_section,

559 Length_of_MtIV / subequal_to_Mt_II markedly_longer_than_Mt_II,

560 Posterolateral_margin_of_MtIV_diaphysis / 'well-developed flange absent or area rugose' with_flange_projecting_caudally_or_laterally,

561 Metatarsal_V / with_rounded_distal_articular_facet strongly_reduced_and_lacking_distal_articular_facet 'short, without articular surface, transversely flattened and bowed anteriorly distally',

562 Pedal_digit_IV / 'significantly shorter than III and subequal in length to II, foot is symmetrical' 'significantly longer than II and only slightly shorter than III, foot is asymmetrical',

563 Extensor_ligament_pits_on_dorsal_surface_of_phalanges_of_pedal_digit_IV / 'shallow, extensor ridges not sharp' 'deep and extensive proximally, corresponding extensor ridges sharply defined in dorsal view',

564 Pedal_phalanges_of_digit_IV / 'anteroposteriorly short, with proximal and distal articular surfaces very close together, particularly in distal elements' 'anteroposteriorly long, proximal and distal articular surfaces well-separated',

565 Shape_of_ventral_surface_of_pedal_unguals / ventrally_concave_in_lateral_view straight_in_lateral_view,

566 Ungual_and_penultimate_phalanx_of_pedal_digit_II / similar_to_those_of_III 'highly modified for extreme hyperextension, ungual more strongly curved and about 50% larger than that of III',

567 Ventral_surface_of_pedal_unguals / 'without a flexor fossa, ventral surface of proximal end convex' with_a_pronounced_flexor_fossa_on_ventral_surface_of_proximal_end,

568 Form_of_flexor_fossa_on_pedal_unguals / without_development_of_flexor_tubercle small_flexor_tubercle_present_within_flexor_fossa ;

MATRIX

Herrerasaurus_ischigualastensis ??000000?00??100----???0010-00000000000????0?00000001?2010000000000-0--01100--0010000???0-?00?0-00?00??00?1????01???11??000?0?0000?0????0???000???01?1?0?0?0-000????0???????????0???1?000-0?0011???????010?000??????????1010?000?010000000???0?000??00-0000100?0????0010?001000?0000000000000000100?0??1??0?00000???200000000?000?0000?????????00?????????0??0-00?1010?00110?1000010??0100?0?00001?0??010000?10000011000?11100000???0000?0-0??0????00-000001001????00??1010???00000?0101????00-1?1?0-??010000?2000000000000000?0000----0?0??100????001000000??000?0-0?00?0000?000?01000-

Nqwebasaurus_thwazi ????????2??????1000??1011?0-00??00?1???----0??????????????????????0-10?1?20111011002010011??0????010?0-0???????0?00?10??????????????????????????010?00?101???0000-??????????????????????????????????????????????????????????????03?11-21211???????--00-0???????10-1??01200?0-?11111?10010?????????????????????????????????????????????0???????0???????????1011101?10000??100?100?010?10??0?00000010?000000110101010122101111001010110??????????????????????????????????1???0?0????00??????????????????1??0??1?????000001000001011111?0010?0010?0?0002001110?001?0011000000001000?0101010

Acrocanthosaurus_atokensis ???010100?0??101?0??10?0?10-?0?1?0110001001000?0?0?0112011??0?00001010??1110--?11?0??0??0?01101111?00??10110??01001?1120?0??1?0??0?????01?0?0????1??0101?0?10000????10-?????????0?????200-?0000-?10???0?10111???0??0?0??101??000021001?00?0?0??1?0???1000?1??1?111110??001?11??0?10??2?1??01????10?1?00??01?0?1?????????0?02??????10?1?0????1?010?????????0???1?101?10?0?100000??10???02??00?101??0?0??01111?0?10100220002??100?0???00???10??????????????10??????????101010??0?0(0 1)0??0????00?11?10?00???01012?1001?011?0??00100?101?????0?0?????0??0??100????001?000-00??0?1010??2?01000-

Afrovenator_abakensis ?????0???????1?101001??0??11010100110?00000?1???????0?201100??0?001010011100--00??????????00010-01??0????010000001?010????????0?0???????????????????????????????????????????????????????????????????????????????????????????????02000100000???????0100-0110110?10-1?0001?1?11???0100?(0 1)?1010?????1?00?????0???????????0???0??0?0?0?100???????11?00??????????????????????????0??0?01??????00?????????1???011?1?0?????12100?2???010000000100100??0????00?00010000100?000000-0?0?01???000100010011010000-?0?0?1100010001000100????0?0??????001????00?0000100?111??1?0?110????01?????2???0???

Albertosaurus_sarcophagus ??1????0000011??0????10?1???0??0??0100000101110?11??002011001100011111011201??000-120000010111100112010101?001010100103000001000000000001?00010?1??1000?00110000100?10-?00???0000?001??00-?0?00-00-1000010111?011100???010??00?10?00??0?0000000?0001010???011?0???????0000011?00????0?0?0???????1??0000?00?00410?00???10??0??00??0??0?00?0????0??0000??01000010???00?000011?001???????00??0?00??01000000010?0???????320?0??-?0000????????10101?100000000?20???1000000?010??0001???00010000000102000101?1101100110000100100000001?1110011001?1000100?2000110?001?0011000001111000?0?1000-

Allosaurus_fragilis ??0010(0 1)01001010?0000?0000?1000010?110?0100100001?1?011?1-000000000101???(0 1)100--?1110100000-01000-010?0???01?0100010101120?00010??000100101?00?00?10?00101001100100-1?10-?0-0-?000000010000-?0000-00-1000011111?000100101010200000021001000000000000010100000111010-10000001011000010011010001000010000001001003000000000000020000001001000???1?0100???100??0001101010000001000000001000000000000001010000111100010101210002111000000000100101000100010000010000100000000101000?1010000100000011010000-00010110001100110010001000101110000?00010000000110001110?1?00110000001010?02001000-

Alvarezsaurus_calvoi ???????????????????????????????????????????????????????????????????????????????????????????????????????????????????????????????????????????????????????????????????????????????????????????????????????????????????????????????????????????????????????????????0---?00?0?0?0-001210000?1?????????????????????3020????????12100?0?1002???????????1??????????000-01?00?000?????????????????????????????????????????????????????1?011?1101000-10020--0110021002001101?00??????????????????????????????????00?11?1??001????????????????????10????????1011??11111001??????00?00?01110??10000-

Alxasaurus_elesitaiensis ???????????????????????????????????????0????????????????????????????????????????????????????????????????????????????????????????????????????????????????????????????????????????????1?00110101??10-101?0????????????????????????????00110?102110001010?0?????????????????0?????????0?00?010010011??00001001004?1??0??????????01??00?2?01??????0000?????????0???????0????0100010011101101????00??0?100010000000?11101220?02???00100100???110100?110101?0???????0-??000?2???????1?????011110?1?1?220?0-???????111?00???0??0?10???????????????1?????????????????00?010-?00?00???0?0?001?0??

Anserimimus_planinychus ????????????????????????????????????????????????????????????????????????????????????????????????????????????????????????????????0????????????????????????????0??????????????????????????????????????????????????????????1?????????????-????????????????????????????????????????????????????????????0?????????????????????????????????????????????0????????111100???10000?12???????2??????????????10000?1010011010?00221??111110012210????10100?100?11?0???????1010000?010????00???00?1000100010??000-???????????????????????????????????????????????????????001?1---?00?010110???0??1???

Apsaravis_ukhaana ???????0?????????????????????????????????????????????????????????????????????????????????????????0?????????????????1???????????????????????????????????????????????????????????????0??001010011100-0-0?????????????????????????????????????2----??????????????110-1?001?12110??1?11?00??0????????????????0???501210?0?210?10-0200-??2?0110????????11????103000-11100110101100?0001101110001010000111110001???1?11?1??????????????????11000-01010--0???1?1?1?000-10????312--1---1-00000-00000-1-22000??11?11112-2001?????1100???????????11????????11?????110?111?00-?000110?11010?001000-

Archaeopteryx_lithographica 1100001010010001010?1?00?00-000100010001???0000010001100111100021?0-0--1100111110-11010000??0?0-000000-010?00?000???0-210??12?010?0?0?????????0?0?01110001???0???????1001011?00??10010000-?0000-00-0-0?00-0?1?0?00-0??001010001002001?212010000?00--10-0??1???0?0-??????10??????0???????0???????????00???0??04????0???20?01??01??1??2?00?0????1?1110??101?2011-0110001111110020??1?00010????000?011?10?00?0?0?1???1?22??0??2??0000000???00-010???1001?1??0????0-01100?2?1????11???0011210000010?2101001??????11?0110?0??0?00??0??????0010?????00?0002000110?11110011?00?00?0?010?0??010-

Avimimus_portentosus ??????????1?1?????????????????????????????????????????????11??1?????????????????????0?000?????0-?01??????0?00?000???11????????????1?1?0?1?10?00?01??120?0?0??0?00-?110-????????0??00????11?????????11??10-0?0?00???????111-??-??????????2??2????-?--??????110?111010012000110?1121101001010001111000000000110601010?0?2?0000???00?????????????????????????2001001100001001100100101000100100000001?111????0???????0??2???????????????0100??1???1000011101010001111?00?0100100011-00011110230010?0000-011011210110100000101000001001100011?11100010112000110111100?11000102-110102010100-

Beipiaosaurus_inexpectus ?????????????????????????????????????????????????????????????????????????????????????????????????????????????????????????????????????????????????????????????0??????????????????0???1?00???1?1??10-101??100?????????????11------0220001100132010001010-0???????????????????????1???0???????0????????????????0????????????????????????????0????????????00??1001001?10000?0100?????????10????????0????00100?????????0?22?????1?00?0000?01011110011??????1???????????????????????0???????????????0???????1??????00???0?0?0??0????0?????010????????10?0??0??????001???11??????????????????0-

Beishanlong_grandis ???????????????????????????????????????????????????????????????????????????????????????????????????????????????????????????????????????????????????????????????????????????????????????????????????????????????????????????????????????????????????????????????????????????????1?11????????????????????????????????????????????????????????????1??????????1111101?01000?0020??000010000000????000????????????????????????????000002?0????????????????????????????????????????????????????1????????????10?0??0002100110?10001010???1100010?0?1010100?1000110???1?001?????00??10????001011

Buitreraptor_gonzalezorum ?????????0000??1?10???0?1???0??????????1???????0???????????00????0??????????????????1?10?010??0-001?0????0???0?10?001?????????????????????????????????????0?????????????????????????1?0?0-?0????01010???????????????????????????0??01-0020???00?00??00-0??110?010-??201210110??12??010???????0??1????0???1?1??01??0???????1??01??10?2?01??????1??2????10??2?1????11??10111001??????????0??1??????1?110??????????????22???????????????0100??????????01?10?0????111?111?1????010????20112122??0112?0?1001???????1??1??????????????????????1????????11?20001????01?0011?10??1-01001?0???1??

Byronosaurus_jaffeei ??000000110000010001?100110-000001110001????00001000??0000???0????110--11101110?0-?0????????????????????????????????????????????000?11?0?1???0??011?100111????010-??11001010111?????0?000-00000-0110-0?010??????????????101?001002201-002000120010--10-0??10-?????????2??0??????210?1???????????1??1?????0???????????????????0???2????????????????????????????????????????????????????????????????????????????????????????????????????????????????????????????????????????????1???????????????????????????????1???????????0?0?01??11???????????0?????????????????????????????????????1??

Carnotaurus_sastrei ??10000000001000---0??00?010000100010001???1?1001110??200000000?000-10101000--?10-11000000001?1101100101000010000??00-???0????1000??2??0?????0??????00?1001??0?0????????????????1?101100??00?01000-100?01000??0?11101000101000??01000000?0?0000000???0-000010101101?10?00??11102?10100?00100?10?10000111?110040?11?0??????00????0?????0000???0??????00--0?0000-?01001010?221?0110?200000-000111000----???????????????????????????????11000-0000??????00?00??00??0?00000100????1000000100?000?0-00000-??000?10000?0111011??011?00?110?0??????????????????????????????????????????????????

Caudipteryx_zhoui 100000??0?111?0?0000????????0?0?0?11??????????00???01?????000?01?0??????1?0101?10-?10?000010??0-00??0???00????000??0????????????1????????????????????????????0??????????????????0?201?001101111100-110?1??0???0?0?????0?10???1101?--??-12??2????01--?0-???????0?0-????1??00????????0?(0 1)0???00????????????????04?1??0???2100??11????0??00?0???1?011?00?110??00????1?001?0?0????1??10?0??0000?0??0001?110?0000?0111110?22??021-100?000000100101001???0?1?10?0?000?????00????????01???2011110??00102?0?0-?100?111111?0?????10??00?????????011???10????01200011?1001??0????01?1?0???1?0??000-

Ceratosaurus_nasicornis ??10100010001100---00?00?0100000001000010001111010101101-1000000001110?11100--0010010?000000010-011101010010100010?00-0?00?0??1000?02??01???000??0???1?1???10?00???0????????????1?????000-???0???0-10?001000?0??????100?100??000?11000?0000??0?0?0??00-0000111?11011000000011??0?00101?00100????10000011011004001?0?1?000?01000?0?01000010??10000?????????00010?01001000???????????????10001100101????0100?100010101110??1??????2????1100??0000????1?0000001001000000000-000101000100100000010-00000-0?11000000000011011000111011110?0?0000?1000???001000011?????????????00?0???????0?0?

Chirostenotes_pergracilis ??1????????1?00??????1101??????????????-??????????????????????????????????????????????????????????????????????????????????????????1?0100??01?1??0???120?011??0?00-1?110??????1????10-0?01111111100-100-10-0?0??00??0????????????1?--????-??2????-??????????????110??211??0?????12???1???????010?1??1?????????5210????0??1?0?????????????0?????????????????20?100???0?10?????????????????????????????????????0???????2200???????10000??1000-100010000??1??010000-000000?12??00?????201111023001121000-01????0?0??????0?0?11??0?0??????00??????????00?????1???0?1??0???00?01?010?0?0??00??

Citipati_osmolskae ??1000000010100101?0?110100-00020111000----0000100101?0010100001000-1101100101?10-11110000100?0-001200-000?000000??010?011011111101?0??01?01010?010112000000-0?001??11111110?0000021-0201101111100-110-10-01010001100-0111------1----------2------------??10-?110-?????210010??1????1?0?1???????1??1000??0?1?52???0???10?????1???0??2?01?1????0??000111111200?????00?110010?1?????1?00?0???000??0?1?10?00???0?????0?22??0????0010000?????0-100????001?1???????0-01000?110??0110???20?11102300102?000-01??????2110010?0?10?????0????????????????0000??000????001??0???00?00?0?0?0?0??000-

Coelophysis_bauri ??000011??0??000---????0?00-0101001?1?10???0?00001001????0000000000-0--11100--00100??0??0-000?0-01?00??0??0?????0???????0?????0000?0???01?????0????1???0?0?1000?????????????????0?001?000-0?0011???10?100-?000??????????1010?010?020000000?????000??00-1??0100?11000?02??0010?0?10000(0 1)0002001000????0??00?0?03?00???100000020?000100?01?0??????00?????????0????0011?00?0?1?0?1000010??0100????000??1?0000001?00?0?0?100??111100?0???001000-0000????100000001101???000??0-00???0001101100????10-1?0?0-??1100?0??0?0000000?00101?0?110???0??0?001????001000000??0???10???0?0?0????1001000-

Compsognathus_longipes ??00?011000????1010?1?00??1101000???0000???000000000??00001000000?0-0--111?0????1?1101??1?000?0-001000-0?0??????0???????????????0???????1???????????????010??0????????????????????0011000-?0?00-00-0-000100-0?000??1210?101000100210110010000000000100-0??0??1010-1?0?0?00010??0?1?0?10?0200????????100010?103????0??0000?0?000?01000010000-1?1000???1????00010?111?00000?0??1???1?0??01???000000????0?0101??0?10?1?22??12??10000011001??0-1000???0???0??0?0???????00?01111???00100001000100010100?0-?1??????000??1????1???00?0??????00?0??????0?0001000?10?001?0011000?00?0?0?02001000-

Conchoraptor_gracilis ???0????0011100?0?00??1?????0?020??????-??????01????1????110??010?0-???????101?10-?10?00001???0-?01??????0?000000??0?????????1??1????????????0?????0?2???1?????00-??110????????0??21-0?01101??1100-110-1??0?0?000??0???111-??-??1?--????-??2????-????????????????????????0??????????1????????????????01??0????11??0???????1??????????????????????0?????1?12?010???0??0100?0?1???????00?0???0????????????????0?????0????0?????00100100????0-100?11?001?1???????0-10000?110??00?1???20???1????010??000-0???????11?001??0?10?????0???????????????????0??????????01??0???00?00?0???0?0??0???

Confuciusornis_sanctus 11-000-01?000000----0?01?0--00110011000????00000?0?01?11-111001-1?0-0???1?0????10-??00?00?10??0-000?0????0?00?000??1?????????0??0???????????????????1?????0?????????????????????0?10??000-?0011000-100?0100?1?000??0???011---?--1----------2----??------??????0????????????????????0????0??0????0??100???1??05????0???20?01??1-?0?????010110??????11111010?0?????1?0?1111110020??1101110001?1000011?11?00?0?0?????1?22??0?????000000?1?000-010????0?1?1???????0-??100?30---??1????0010-1?????1?211011?11?????2-20?1?????0??0??1??????00???????01??1??2110???111100??001?10?01??0?0??000-

Cryolophosaurus_ellioti ???????????????????????????????1??1?1??000??0??0????0?2?????0?00001011??1100--001000??????00000-01?00??10?10??00????10?0?0???????0?????????????????10101?????000??????????0-????0???????????????????????0-0110??0???????????????0(1 2)??0?0000?????????????????????10-1(0 1)????00?????0???0??????00????10010??1?10?0???????00???0????????00?0?0????0?00??????????????????????????????0????????10000???????????????????????????????????????????00?????????????0?0??1??10?????00????????00????????00????10??????01001?010??011?0??0000??????????0??????????001100000?????????????????????????????

Daspletosaurus_torosus ??100020100111110100??00?10-00000101?000???11101??????2??0001100011111011101100011?01?010?000?0-011201010110?1?101001030???01?0000???????????0??????????????????100?10-?0-10?0000???1?000-0000??00-100001011????????211?10100001020000000000000000010100??????????????0???0????0????????????????????????????0???????????????????????????????1?111????????????1???????????????01????????????????????????????????????????????-????????0????????????????????20??????????????????????????????????????????????01?0002???1?0?10?????01?111???2??????????????????????1????????????1????????0?0-

Deinonychus_antirrhopus ??000000200001?111101000?00-000000010001000000010000110??1000001010-0--1???100?110??1???0?10??0-00??0???1001000?0??11031?110100?0??????????1????????00??0????0?0??????????10??????0011?00-?000??00-0-000101?1001???110101011000002100?001000000?000100-0001111?10-00101200011?0101100201000000001001011101100?????0?????00120010010?1001?1????1?12??????1?21110??110?101010?1?0???100010001000?00?11100001110101111122000212?0010000001?00-1??1100001?1??0?0000-111001210??0101???001111222001021010-0101011111111100001000001011111?00101??10?000002000110100110011?10?100011101001010-

Dilong_paradoxus 0-100020000110?101001100?10-00000011100????010210100012011001100000-11011100--00100011001000000-001200-0011?01010??011??0?????00000100101?00000??????1?1??1??0001??0????????????0?0-11000-00000-00-0-0000-11111111101010101000010200000000000100000100-0???????10-1?100000010??0010?0?0101?????????????1????0?????????????0?0??????2?000?0??1?????????????0?????1?1000?011???10001???????????????????????1?1?1?1110022???21????00000??1?0??1??010000??0??20000100?0?0??11??????????????0??????0??0????11?11?0001?00??0?10??0?001?111??010???10???0002000111??01???????0?00?0??0?2???000-

Dilophosaurus_wetherilli ??001020?00??0?0----1?????0-010?0010011(0 1)0(0 1)1??0?0?1001?2010001000010?0--1111???0010?0????0?00010-01?0????111????000?011????????0000?0???01???000??1?1?0?0?0?10000??101?????0???????1?1?1?0-???0???0-10?00101?01??????????1010?000?1000000000??1?10???00-0110110?1101?0011?0011?0??0?011000200?10?10000??1100?03000???0?0000020?000?01000?0???10?0??????????0??100010?00?0?100?10000100?0100?0??010?01?00?0001?00100011000?1?11000?????11000-???0????1?00?0001101????100?0-00???100?00?100????10-????0-??11000001000000000?0000??00110?000?0??100????001000000??0?0?10??0??0000?0?2001000-

Dromaeosaurus_albertensis ????????2?0??1??1?1??100????0???0?010??10100??0????011???100??0101??????1???????0-121?1101???????0??0?????????000??01031???0?0??000100001?00000?0?0?0011?01010000-1110-10-100000000011000-00000-00-0-000100111010111101010?000110?000?00000000000?0100-0???????????0????????????????????????????????????0?????????????????????????????????????????????????????????????????????????????????????????????????????????????????????????????????????????????1?????????????????????????????????????????????????????????????????????????????????????????????????????????????????????????????010-

EK_troodontid ???????????????????????????????????????????????????????????????????????????????????????????????????????????????1????11??????????0???????0????????????????10????1????1??0?????01?????????????????????????100???0????1???????????????????????0???????1???????0-???????????????????????????????????????????????????????????????????????????????????????????????????????????????????????????????????????1000010?01????1?22?0?2???0000?????????????????????????????????????????????0?????????????????????????????????????????????????????????????????????????????????0011?00?1????0???1???10-

Eoraptor_lunensis ??0000000000?000----0?00?01001000000100????0000000001?01-0000000000-0--11100--001001??0??-000?0-00??0??00000000?0???10???0???0000???????????????????????????????????????????????0?101?000-00?0??00-0-0?010?0??0?0???????101000000010000000000???0011?0-0?????0?0?????????0?10?????0?00??0?00????????0??0????01???????0????0???0?00???0??0??????00?????????0?????00??00?0???0?10?0??????????0??0????????10?01???0??0110???0100???0????00000-0??0???????0?00?100?????????0-?????00000?010?????00-1???0-????0????2??0?0???0??????????0--???????00????????????????????0-?????0??????????0???

Epidexipteryx_hui ?-?????????????????????????????????????-??????00???0???????0?001?00-???????????????0???0?0?0??0-001??10????????00???0??????????????????????????????????????????????????????????10?2?0?00???1?10-00-10001??001?00???????010?011100?01??2120?101?0?1??00-0??????0????????????????12??000???100????????00?0????04??0?0?0?2100???020?1??2??100????????10??????2?1?????0?100?101?02?????00010?0?0??001????????0??????????22??0??????00001?010?0-200011??????????0???????0??00??????????0?010102?????110????1????????????????????0????????????0?????????1??0001???111000110????0?0???0????????

Erlikosaurus_andrewsi ??100000100110000?-0?101110-00001101000????0?00????11????1000001010-0--?1100--?110?100000010000-00100??0000010000?0??0???1012100001---010????00?0?1000000000-??00-????????10??000?1010?011?1011010-101000-011?0000-0100011-?----0220001?0013111?001010-???????????????????????????????????????????????????????????????????1??????????????????????????????????????????????10?????????????????????????????????????????????????????????????????????????????????????????????????????????????????????????????????????????????????????????????????????????????????0????????0??0??????????0????

Eustreptospondylus_oxiensis ??0??020100?1100----00000?1100??0001001000?0???????1??????????000?1-11011000--0011011?00?000010-010?0????010?00000?00-????????0??0?????01?00010?01010001?0111?100-??10-00-10000??0??1?110-?0????0101001????????????0?????010000002000?0000000101000100-0110111010-11100101011?00?10001000200000011000001000?03000?0?0?(0 1)00002???00?00????????????????????????????1?00100?0200010001?000????????????????????????????????????????????????10???0??0???01000?00000011000000010000001010000100010?0?01?000-010100100000001000100010000011100000001100000000100011??00??????00000100000?001000-

Falcarius_utahensis ??????????????????????0???????????010??001?0???????????????????????????????????????10?0000?0?00-00??0??????????1?10010???????????????1?0??00?00?1?01000?000??0?011?110-10011010??0??1?00100000??00-1010?????????????????????????0?20001(0 1)00101110001110-0???????1101?212100010?1121111201020011101001010?0010041101000?1000120010011000010???101111???1010?10010?1?101001?1001?0010001111001100?00011001001010001111022000211100100000010111100111010101?00000011010??1110010101010001121023001022000-010?01101011000000100000101111100010?11??010000100011010001001100??001000???001000-

Gallimimus_bullatus ??0???00210000000101?101110-0?000???000-???00000000?11001?110?000?0-0--1???1110112000100?00?0?0-?000?0-001?010112??00-??01?010?10?0?110?1?00001?010?0001001??00011??110?0-100010??10-0??10?1010-00-0-0?01?1?1?1000-0???011------1---??-?-?-2---???---?????10??010-??202210010??12???0?0??????00?1??0000??0?00401??0???100?0??00??0??0?01?0????0??0????????011100??11?000002?0?0???2?0000??0000??1?0??001010?1?????0?2???0??2?00011010?1?010100?100011?0??0????1010000?010??01001-?000100010001010000-110?????00?1001?0????00?00??????00???00?0?0100??0001???00101---100?01?1?010?0001010

Garudimimus_brevipes ??0000002?000001001?0101110-00000111000----0?0000000??01-0110000010-0--111011101120?01001-000?0-00?000-00?00?00?001?113?01?01001000?11?0???0001?01?000010?01100?11?????????0???00???-0001001?10-00-000?00?101?1000-0?1??11------1----------2------------000?-1?????????????0-???2??00001010001001???0000001?04010100?0??0000???00?0??0??0????????????????????????????????????????????????????????????????????????????????????????????010011100010011100?1001001001000101001000101020??????????????????10101100011001000110010001011100000?0100?010002000110?00100011100000?1101020000011

Giganotosaurus_carolinii ???0?00????????0????10????10???1?001000100??01?1?0????2???????????1010??1110--??1?0???????01101111?01??1???????1?0??10(1 2)??????????1???????????0???0???101????????????1?????0-????1?????201-?0?0???10???0???1?11????????????1???000200?0?10?0?0??0?0???110001??1?111110??(0 1)?1?11??0?100?2?0??0?????10010??1?01?????0???0?000?01??????000???0???1000??????????0???????1?10?0?????????????????????????????????????????????????????????????0???10000?110?1??0?0?000?11?????101010??010100?0???????11?1???????01012?100??01??0?????0??10111???0?0??1?00????1???????????????????????????????????

Gigantoraptor_erlianensis ??????????????????????????????????????????????????????????????????????????????????????????????????????????????????????????????????????????????????????????????????????????????????20-0001111011100-110-1??000?00?0-????????????????-???-??-2------????????????????????????????????????????????????????????????????????101012?0000?0??0??????????1???????????????1?0?100??110?110002???00-00000000?????000101000??????????????????????????????????????????????????????????????????????????????????????????111110????10001??????????????????????????????????????1?????????????????????0?0-

Gorgosaurus_libratus ??101020100011?101001100110-000000010000???1110?11101?001100110001111?11110????0??121?0000001?0-00?20101011??10?0?????3?0000100000000??01?00000??????????????000????????????????0?00??000-00000?00-100001011110?0??0????10100001020000000000000000000?????????0???????0?0??????00??0??0??0000?0?????00?1????0??????????????????????????000???????????10?????????1???0???????????????????????????????????????????????32????????????????100101011100????0?12100?100?000?0101?0001???00?10010?0010????????????????????????????00???????????0?0?10???????????????01000110??????1????????0?0-

Guanlong_wucaii ??101000000111110100110011110000?0111000010010110001012011000100000-11011100--0?100010000-00000-001200-00110?1012??0??300?1???00000100001?00000?11010001000??000100010-10010?0000?001?000-00000-010100?00-1111??1??0????101000010200(0 1)1001000?000000100-1??????010-10101000010?1001001001010000001001001100100300010?00100000000?0??2000000?????11?????????00010?1?10000?01001100001001110001000001010000010101011100210002121000????0010010101010000000?02100110010000110110001000000100000000-100010011?01100011001100100000101111100?2000?1?00100011001111001?00110000001010002101000-

Haplocheirus_sollers ??00??0011011111010111001110000100111000?0?0000100001?01-1100000000-0--1000111?112010000?-10000-00??00??010011000??010?00110100000021?001?00010?010?0111??00-00110??????1?110??00?1011110-0000100100-000111010011??02100101000100120110010001101000100-0??????00-?--101?00?10?101110100101??00001??1?????10103000?0?0?(0 1)?00??00100?00?00000??1?01??????????11010011100001010011011011100?00?1100001?1000000110111110022011212101010001010???????11-?01002?000001001??00011010100??01001100000110110?0-01??0110101000000?1110001011110?001011010??000?20???1?10010?????00?00?010002001000-

Harpymimus_okladnikovi ??00000021000?010?0??1011?0-000?0?11100----000000000????????0???????0--1110111?112010?00?-000?0-00?000-00100100?????????????????0???????????????????????????????????????????????0?0010001001011000-000001010??0000000-0011------1------?--11?0??0?--?---?????1010-???0??0000-??02?0?0001?2??0?00????00110?110401?10???1000020000?0000001????0?0110?????????11???1?11100?012?0?00012000010000000011?10000010101011110221002111000111100100101001????11?0??0??00100?00000????010????00???????????????????0101?00???0?????????????????????10????????0001000110?00101---100000?0?000?0001011

Huaxiagnathus_orientalis ??0???000001?0?10100??00??11000???11??0????0000?1000?????110?0000?0-0--?1?0????1???1??????????0-?0??0????0??????????????????????0???????????????????????????????????????????????0?001?000-00?00-00-0-0??????????????????10??0??00?0?1-00100000000?0100-0??????0????????????11??10??0??0?0100????????10?01??10???????????0????00?0?120?1000??1?0000???100??00010?111000000100??????????00????00?00?0100?001??0??10?1?22??12111?000000?01?00-10001010???0??0?0??02??000?111?????????000100000001010000-?1??????0?????????????0????????????0???0????00?2000110?001?0011?0??00?0???020??000-

Incisivosaurus_gauthieri ??1000000101110100000100110-000111110001???0000100001100010000?1000-1001200001000-1010000010000-00100100000000010010102011012101101-00001?0110??11?012001000-?0010?11??1100-??0?0?2011?01111111000-0-0000-000?0?0110??0?101200?00?00??1?2001101?10--10-???????????????????????????????????????????????????????????????????????????????????????????????????????????????????????????????????????????????????????????????????????????????????????????0?????????????????0?????????????????????????????????????????????????????????????????????????????????????????????????????????1?????????

Limusaurus_inextricabilis ??1000101001110101?00?000?11?0?1?01?000?---00000?000??01-?000000000-10012100--00120??0????00000-00?1????0000?0?020?00-?????01?000???????????????????????????????????????????????0?20-?00??01?1110-?0-1000-?0?10?0??0????11------1-----------------------000??0?1101?1?2?0000-??01?0?0???????????????00?1????0?????????10000?000???020000000-0??1101000-1??000100011010000?10011011?00?00000?000000----?0000-00010101(1 2 3)10-00011--02???000000-000110001000?100100100100000100???0?0000?00-??000?0?00?00?1???00?010?0?0?????00011????????????0??10??????1???????001?0???00??0??00???2?00000-

Linheraptor_exquisitus ???000002000?1?1101?1?00?10-00000001000????000?0100???0001000101010-???1110100?10-1?10000?100?0-00?200-00001010?0?0?10????????0000???????????????1?10101????????????????????????0??0??000-000???00-0-0?01011????0??111???01?????02101?0?1000000??00100-???1111010-1??00?0??11?11??1?????????????????00?????10?????????10?0???000??001000?1101???1?111???????????1???1????10?11???01???1?????10???????????????????????????2????????????1?0?????????????????????????????2????010????01??????????????????1???????????????????00?101??111??10???10???0??2000110??011001??10?10?0?10???01010-

Mahakala_omnogovae ???????????????????????????????????????????????????????????????????????????????????10??000???????????????????????????????????????0?????????????????1001??1???????????10?0-10?????????????????????????????????????????????????????????????????????????????????????????????????????????????????????????????????401??0???????1??01??10?2?????????1???????????????????????????????????????1???10??????1110?0??????????????????????????????1000-0???0-??11?1???????0-11100??????????????????????????????????????1?11?111?0?0???0?0????????00??????0???00??0001????01?00??010?10-?1??0?0??010-

Majungasaurus_crenatissimus ??10100010001000--??100000100001000100011100?1011010112010000000010-101?1010--010-110000?0011?11011111011000100010?00-0000001?1000012??01?00000?1??1?????0???0?0?????0-?????????0?10110???0?00?????10?0?1000100001001000101000000110000000000000000100-0000111010-1?10000?011?020001010001001101?00000010110030??1?001100002000000110000000?100100?????????000-00??0?0?002210010012000??????????0???????????????????11???????????????010010000010001000?000100100000010????????????0???0??????-??0???????????000000??00100011101101010000111100010000100010??0??0???0000000?0??0?0000011

Mapusaurus_roseae ?????0?????????0?-??10????10?0???001000100?1?1?1?0??1?2??1???????01010????1????1100???????011?1111?????????????1?01?10????????????????????????????????????????????????????????????????201-?0?????10???0???1?1???0??0101?????????0?00?0?10?0?0??0?0???110??1????11111???001?11??0?100?2????0?????10010001?0????1?????????0?01??????1001??????1???0????10?????????1?1??0???100?0???11?1????????????????????????0??0??????????????0?????0???10000?110????????00??110????10??????????????????00?11?10?00???0?012?1001?011?0????10??10?110000?00?0?00???1?100011??0??????????0?1?10??????????

Masiakasaurus_knopfleri ???????????????0--??100???0-00???0100??10000????????????????????????????????????0-??????????????????????????????????????????????????????????????????????????????????????????????????1?110-01?11100-1000?????????????0-??????????0?00?0?00?000?00010100-0???????1101-10??00?0-1?2110?0???01??00001??00010?01?0???01?0?110?0020????0??0?0???????????????????00010???????????100?1001?????????????????????????????????????????????????????????????????????????????????????110?01000000????????????????????0100100010000100100011101011000000?????????1??1001111001?????000?0???00????001?0-

Megalosaurus_bucklandii ?????000100?1?000---????0?1000???-1100001101????????????????????????????????????????????????????????????????????????????????????????????????????????????????????????????????????????1?000-?00???00-??010100????0????????????????0?0001000000?0000?010100????????0-?????????????????0?(0 1)?1??00?????100?????0???300010100??0??2????0??0?1????????????????????0??0-?0?0??????2011?0201??????00?0?????????????????????????????????????????010?10000110000000?01000?100000100??00000????000100010?1??0??????00000100000001000100?1010101100??0????????????????????????????????001?00?0????????

Mei_long ??00000011011?00?-??0?00?12100000?11000?????00001000??01--11?0?11?0-0--12?01110?0-1100?000??0???001100-0???????10???11??????????0???????????????????110??????0???????????????0??0??????00-0000??0110-0??????100????1????10?0?0?001201-0020???0???0--?0-0??110000-?--101210110??101001?0?11000?00????00?00111040??1??0?1000?0001??2--0-0?0?1?0???12???1111?20110?110011010000010?11100110???????0?????????????1???????2??????????0000001????????0--00??1?0011000-0110000????01001-0101??10???0??20??10?10?01??1110110?0?11?000?????????011?0?100??0112000110?00100011???1?1??10112101010-

Microraptor_gui 100????01000???1??????00??0-0??????????????00000???0?????1?0?0??????????1?0?????????????0??0??0-00??????????????????????????????0???????????????????????????????????????????????????10?0???000??00-0-0????????01???1????10???01?02?0??0?10000???00--10-?????????????????0?111??1????00??01?????????100?0?1?004???1????10??1?001??1--1-01?1??????120011101??0??????1??111110?11???0???110??????000?110000000101?0??0?22??02??1001000000100101?0?111??1?1???????0-011?1?20--?010?1-0011121?2??011011010?1??????11?111????10???0?0??????00??????0?0?0???0001???10110011?10?11?0??01?101010-

Microvenator_celer ??????????????????????????????????????????????????????????????????????????????????????????????????????????????????????????????????????????????????????????????????????????????????(1 2)0????11?1101100-????00-00????????????????????0???????2??2????-?--??????1101?110???????0010?01?1?102??11000?001001000??0?10?0???????????1?11??00??2?????????????????????2001????00?0?0?010110??02?0010000?000?0???????0???0????????????2???0010?00001000-1001101001???1010000-00?0010100?0101???20????????0?????????1??11?10121010000100??000???11?001??001000000120001101??????????????????????????0-

Monolophosaurus_jiangi ??001000100101?10000?00?01110001000?0000??001111000100201100000001111??11100--00100000000-000110011000-0011000001??010????????0000?????01???00?????1010100?100000-??????????????0?0011110-?0000-00-100?01001????0100211?1010000002000?00000000000?010100??1101?10-1?100?01011?0001000101010000011?0000?1001003000?0?????0?02????0?????0??????????????????????????????????????????????????????????????????????????????????????????????0100100000100100?0?0100001000000000-??0101000100100?00110-100?0-???????????????????????????????????????????????????????????????????????????????????

Mononykus_olecranus ????????????????????????????????????????????????????????????????????????????????????????????????????????????????????????????????????????????????????1100?????????????1001011??0??1??????????????????????????????????????????????????1-212?1??0????--10-0???????0-?--1010?11????121101011020010000-?101???1010??2??1?????0121?0?1??????????????????110???001000-01?101000023000010111-0021000111011?10101111101101100221112-?111011?120?0?0-???????????????1?00111??00?30---???1???00?0-0000??-?????0-?10011112-20010010111000011011110011?11???1011??1101101001?0011000102-11000?010100-

Neovenator_salerii ??00?01010000101000010000?1100010011000101000101110?????????????????????????????????????????????????0???????????????????????1???????????????????????????????????????????????????????1?100-?000??00-??00?????????????????102100000?100100000000010?010100001111011111100001011?0001000200000100011011011100100?010???????000100000100010?10??100100????????0001101?101010?????????????????????????????????????????????????????????????0110100??010001000??000001000??11010100101010000100000011000000-00010120001100110010101010111110010001101001????100?????01??????000?01?10002001000-

Nothronychus_graffami ????????????????????????????????????????????????????????????????????????????????????????????????????????????????????????????????????????????????????????????????????????????????????????????????????????????????????????????????????????????????????????????????????212?????????2????20?0?001111???????10???04?10?0?0?2010??10?0??0?000100?????00????101??00?1?00???10000110?1?111101??100?100000?????????0?????????22????????000000001011120011??1???1?0??100???1100?1101000111--2?0121?0?11102?10???0??112111100??00?000?0010110???111??1101?0??0?2000?1??0001010-0000001?0110?0?0??0-

Ornitholestes_hermanni ??00?0000?01?00101011100??10000?01101000000000?0?001110001100000010-10011100--00101?01000?10000-000201000?001000000011(2 3)0????100000???0?0????0?0?01111101?100-????-????????????????0010?10-?0000-00-100?00-011100011010001012011102101-001000000?010100-????????10-1?101200011??1111?11?101??01001??1001010110?0??10?0?10?0020000?10?0??????????1?1??????????????????????010?1100001001?0??0?0?0?0?????0???????????????????????????????1?0101??0100000?0?0010011000000?1????010?01000010?000001?10000-0????????????10?0?10?????????????????00??0?????????????001??????0?100?0?0?0?0010?0-

Ornithomimus_edmontonicus ??0?????210000???11???01????0?020?????????????00???011?0111100000?0-0--????1111112?00100?00???0-?00?????010010101??011??????????0?0?11??1?00????0???000?0?0??0?0???1110?0-10?010??10-0??10?1010-00-00??01?1?1?111??0???011------1?-???-?-??2??????????????10-?010-?1??22100???102???00??0???01??1??0?00??0?00401?00???100?0??0???0??0?01?00-??0??0????????11111???01?000002?0?????2????0??0000??1?0?0001??0?1?????0?22??0??2?00012210???010100?10001100???????1010000?010??010????00010??10001010000-110?????00?1001?0?10?0???0??????00???0??0?0100??0001???001?????100?01?1?0?0?0?01000

Oviraptor_philoceratops ???100??0?11?????1????10????0?0?0??10??-???0??01????1????01000???00-????1?0101?????1???00??0??0-001?0????0??0??00???10???1?1?1??1?1?0?1???????????????????0????00-??????????????0?21-??011?1111100-110-10-0?0?00???0????11-?????1?--??-?-??2????-???????????????????????????????????????1???????????0?????????????0???????????????????????10?????????1111??????????0??1??1??0???????00?0???0????0???1???????0?????0?????0????00?0000?????0-1???????0??1??????????????????????????????????????????????????????????????0?10?????0??????????????????????????????01?????????????????????????

Parvicursor_remotus ?????????????????????????????????????????????????????????????????????????????????????????????????????????????????????????????????????????????????????????????????????????????????????????????????????????????????????????????????????????????????????????????????????????????????????(0 1)1??2?0????0???????010???????1?????0121???10??????????????????????????????????????????????????????????????????????????????????????????????????????0???????0--?????2?01?0?0-?1????30---1---1--0000-0000----1(1 2)00???1?011112-200100101110000101111100011000?01?101211011010010????000102-11001?001100-

Patagonykus_puertai ?????????????????????????????????????????????????????????????????????????????????????????????????????????????????????????????????????????????????????????????0????????????????????1????????????????????????????????????????????????????????????????????????????????????????????????0?00?01?0????10000????1???302011000??012100?002??????????????????????0?1000-0?????001???0??011?111002100011???????00?10?10??????1??1112?????011?11010???????1?0???????00100101????0211011---01001???????????????0-0??????1112000100011100001111111??10111??000010(1 2)001100110??????1????000??????010?0-

Pelecanimimus_polyodon ??00000021000101000111011?0-00010011100-???00000?00000001111?000??0-0--1120111011202?1?01-000?0-00???????????????0?010?00???1??10???????1?00?01???????????1???01????1?????10????0??00?001000?1??00-0-0001010??1?0??0????1030001103201-102110201010--10-0???101010-1?????00010?1???1000?1????????????????????0?????????????????????????00?110??????000???101?????1?11000?0?200?0001?00?0100?0000011??00?1010011?1110?22100112100011210????????????????????????????????????????????????????????????0??????????????????????????????????????????????????????????????????????????????????????

Piatnitzkysaurus_floresi ?????0?????????1??????????1000????110?00100?1??????????????????????????????????????0????????????????0????????????????????????????0?????01???0????10??1?1?0?10?0010?010-?????????00????0?10????????????0?????????????????????????0?1?01?00?0??0?0????0100111110?10-1010?1?0011????10001?1010?????11000??1?00??30?0???0???0?02????0?????????????????????????0??11?0?0?00?0?1?0?10?00??0??100????0??????????????????????????????????????0100??0????????????010000?????????1?00????0000?010?????11-1???0-??110010?0100010?0100??0??10111???0?0????0??????????????????????????01?0???????????

Proceratosaurus_bradleyi ??10??000000011101001100111000?001110000???0??1???010?001100010000?????01?0????01????????????00-?0??????0110?0000???112?0??0??0?000110101??011???????????????0??????????????????0?001?000-00001000-100000-1111100??0???01010000102101-0010000000010100-0????????????????????????????????????????????????????????????????????????????????????????????????????????????????????????????????????????????????????????????????????????????????????????????????????????????????????????????????????????????????????????????????????????????????????????????????????????????????????????????????

Rahonavis_ostromi ??????????0???????????0????????????????????????????????????????????????????????????????????????????????????????????????????????????????????????????????????????????????????????????????????????????????????????????????????????????????????????????????????????????????????????????012011100000?10010010010?041001?000??10120010010020??????1?1112??????????????1100110??????2????????10001010000????????????????????????????00??????01000-21011110011100010000-011100211??00111--0011210000011121011010001112-20110000100000101111110011100100100012000110100110011011110101110?001010-

Rinchenia_mongoliensis ???1?????011100?0?00??1?????????????0??-??????01????1????0100?010?0-10?????101?0????0?00??????0-?0??????00?000?00????????1???1??1??????????0??????????0???????????????????????????21-0?011?11111?0-??0-10-0?0?0?0??0???111-??-??1?--??-?-??2????-?????????????????????????????????????????????????????????????????0??????????????????????1?????????????11??????????0??1?0?0?0??????????0???0????????1????????????????????????00?000??????102???111?01?1??????????????????????????????????????????????????????????????0????????0??????????????????????????????0???????????????????0??????

Sapeornis_chaoyangensis ??0000000?010??1?1??0???????0??????1?00?????00001000??????11-002?00-???12?0????10-11???0??100?0-0000?0-0??????????????????????????????????????????????????????????????????????????10-?001001010-00-0-0-00-0?1?101??0??0010100?100?011-2120?2--------?0-0??????0??????????2??????211???0?????0???????????????05??01??0?210-0??1-0000??????0???????????111??2010-?11001101111012?001?011100?10000001111100010101111?1022??020010010000001000-11010-???1?1??010000-0?100?3110101101-01010-10001011010011010?11112-2?11??00100?0????????????1????????11??1001???11100010??0?10??10?0?0??000-

Saurornithoides_mongoliensis ??1000??1100000??10?01?0????0???0?01???01000???0?????????1??????????????110?????????????0?????0-00??0????????????????????1?01????00?11????????1????????????0-0??0-????????0-?11?0?000?0?100000??011??0?0???????????1?????0?000??02101-0010001000101010-0???????????????????????1???0?00?????0000?00??0?000??0?010?000???001????00????????????????????????????????????????????????????????????????????????????????????????????0?????????????????????????????????????????????01?1???0??111023001020000-0???1111111011??????????????????????????????????????????01??????????1???0???1???1??

Saurornitholestes_langstoni ???????????????????????????????????????1??????0??????????1??????????????????????????1?11?1?????????????????????????????1???????????????????????????????????????0???????????????0????1??00-?0?????????0????0???????????1????1???0???0??0?1000000?000100-???1111010-1?1??200011?11?10?12??00??????1??101110110?411010100????1??0???1??2?01?1????1??1????????20???????????????0?????1??00?0?00?1?000??1?0?00?010??1111022?002???00100000?10?10210110100??1?0010011100100??????????????0???1????0?0??0??????????????????????????01011111???101??1?01100110001100101?0011?10?10?001?0?001?10-

Segnosaurus_galboensis ?????????????????????????????????????????????????????????????????????????????????????????????????????????????????????????????????????????????????????????????1????????????????????0010?????1?1??1??101??0-0?1?000??0???0????????0???001?0?10111?00101???????????????????????????2??????????????????????????????1??0???????????????????????????????????????2???????1???1??11?0?0??110110?????????????????????????????22???????00???????1011110011102010000101000-01000?21010????01000011110?1?102?101??0??????????????0?0??1??0???????11???11?????0??10000????001010-?00000??01102001????

Shenzhousaurus_orientalis ??00000021000001000??1011?0-000?0011000?---0000010001?????1???????0-0--1120111?1????0?00??0?0?????00?0-0???0?01????0????0???1???0????????????????????????????0??????????????????0?001?001000010-00-0-000??1?1?0????0?????1------1---1-----11001001--00-0?????????????????0?????????0?00??200?0??????00?0001?0411??0??????000?0000?0??0???0??0?01???????????????????????????????????????????????????????????????0110?221???1210001011001000-100010101000???1000100?0001010010100?1?010100000000010000-???1????001100??00100??????????????????????????????????????????????????????????????

Shuvuuia_deserti ??000000110000010101?101102100000?11000-???00000000011000-11001-1?100--122011101120200000-10000-001000-0100000000??10-??01?120000002101001--010?011111?00100-0010-??11001011110001100?001000001000-0-0000-0010011110??001????0??03201-212110202?10--10-0??10-??10-10100211110?11210?101?0???0???0??100??01?105020?1?0010012100110100200101????1110110???001000-0110000000230000101?1-002????110?11?10101111-01101--0221112---1101121201000-10020--0111021012001111000?30---1--11-00000-0000----22000-010011112-200100101???0??1???101?001??????101112110110100100011000102-11000?011100-

Sinornithoides_youngi ????????1?000???????????1???0?00??????????????00??????????????01????????????????0-???????????????0??????10?????????0?????????????????????????????????????????0???????????????1????000??00-?000??0110-0???????????????????0?000??0?101???1??0100?10?010-????????10-???0??101?????????1???????????????????????0?????0???10??1??01??1??0??1?0????1?12??????1?20110???00?1??001???????2?0?10????????????10?0010?01??????22??02????0?0000??????????????001?1???????0-1???0?0??????01???00?1210??00?0210?0-?1???????1?011???????00?????????00??????0???0???????????0100011?0?10????011?101010-

Sinornithosaurus_millenii ?000??001?000??1111?1000??0-00001?11??01???0?00010????0000000?010?0-0--1??0100??10111?1000100?0-001201101?0?0??0??0010??????????0?????????????0?????????????????????????????????0?101?000-00000-00-0-0?01010??0?0??1????101010000?10010?1?00000?000100-0???????????????????????11???1???????????????????????04????0???????1??0??????2?????????????00111?1120110???10?101110????????????0????????0????0000???1?????1?22??02?2?00?0000?????0-1???11??01?0???????0-11??0?211?????1???011121????011211010?1??????????1????????????????????????????????0??1001???001?00???10??1?0???1?001?10-

Sinosauropteryx_prima 000000000001?001??0?0?????0-0000????0?0????0000???????????100????00-0--?1100????11?????????0??0-00??????00??00012?0?10??????????0????????????????????0???????0??????????????????0?001?000-00?0??0??0-0?0????????????????10100?1002101-0?1000000?000100-0??????010-1?1?0?10?10??11??0?20??100????????10?01???03????????000?0?000??1120?10000-1?1110????????00010?111010000200?00?00????01?0??00000?1100?0111101?1110?22??121110100010001000-1000???0???0??0?001020?000?111?????1???000100000011?100?0-?1??01?0001??0??0?1????0?01??10?00?0???0??0?00?20001101001?001100??00?0??0020??000-

Sinovenator_changii ??1???0001010??0010?1100111000?11???000??????000???011????????0?????????????????0-???0?000???????01????????00??12??011??????????0?0?00100????00?0??0100?110??0?10-??11001010?001??1?0??0???000??011100?0??0?1?0?0??????0101?00??0?2?1?0?10?010??1?01?0-?????????10???0?210?10??01???00??????00?01??100???0?1?401?00???10??1??01??2??0????????????1????????20110???10?101????????????????????????0???10?001??????????????????????0000???????????111????1???????0-11?00?211??010????0011210??0011210010?10?01?11110110?0?11?00??11?111?0011???1000?10??0001???001??????00?10???1?1?1???1??

Sinraptor_dongi ??001000000000?10000000?010-000110110000?0000001000000200000000100101??11210--?1100110000-010010011000-00010000010?0113000001000010000101?00000?10?00101000100100-??10-?0-0-?0?00?1011000-?0000-010100?0111010000??010101010000002100?00000000000?010100110111010-10100001011?00110011010101010011000001?0100300000?????0002?00?0??1?1001???1??1??10????000?????1010100?0??????0????????????????????????????0??11101210?????1000000100100101001???000?0??100001000000001100??11010000100000011010000-00010?1000110011001000101010111?000000?00?0000001000011000?00110000001010?0100?000-

Stokesosaurus_langhami ???????????????????????????????????????????????????????????????????????????????????????????????????????????????????????????????????????????????????????????????????????????????????????????????????????????????????????????????????????????????????????????????10-1?100?00011?00???0010001000??1?0???00?00??030001000???000????00???????0???1????????????????????????????????????????????????????????????????????????????????????????0100110??1100?0100??21001100?0000011?1000?00001010001000001?0?101????????0????11001010101010110001??0??????????????????????????????????????????????

Struthiomimus_altus ??0???0021000??1?10?110?110-0000011?000----?0000100?11???1110?00010-0--11?01111?12000?0010100?0-?000?0-0010010112?1011??01?0???1??0????01000????0??0000??0???0?010??110?0-100010?000-0001001011000-00??0101?1?1010-0???011--?-??1----------2------------??10-?010-?????2100???002???000?????0???1??0000??0?00401?00???100?0??00??0??1?01?0????0?10????????111110??11?0000020010001200001000000001?0?0001010011111100221001?2?000112100100101???10001100?10????101000000100100001-0000100010001010000-110?01?00011001?0010?0?000???1100010?0?10?0100?200011??00101---100?01011010?0001010

Suchomimus_tenerensis ???101201????0?0?-???1?0?111???0?01?001000?01?????????????????000??0???????0--??100????????000?????0??1?????????????10????????0???????????????????????????????????????????????????????110-????0-???????????1??????????????3???00022000?01?1?2????0???0-1?01??1?10-??0??????11??0?1?0?1?1??10????11000001?01?0???????????0???????0??0???0????0100??????????0??0-?0?0????001010102?1??0??21001??10?????????????????100???0?????0100????0?0?10000?????1??0?01?0????0?0???00?????0?(0 1)(0 1)00?0????00?11?10??0???01011?1001?010?0??0??0??10111???1?1??0?00????110001??????????????????????????????

Syntarsus_rhodesiensis ???0?0211?0??000-??0???0000?0101001?1110??1010?001001?11-0000000000-0--122?0--00120100???-?0000-00?00??0110????00??010??00??1?0000?0???01???100??0?0?0?0?0?10000??0010-???0?????????1?100-??0011?100-?10101000??????????1010?010?010??00000??1??00??00-0110??0?1100?00???001000?0000010002??100010?00??0?00?03010???1?0000020?000?00001?1?0-1??00?????????0??1000?1000?0?1?0?10001100??1???0??0?0??1??000001?001000110?0?11110000???011000-0000????1000?0001101???0001?0-00???0001101100010110010000-0?110000??00000(0 1)010000101?10110?000?10?1010??1001000000?10?0?100?0??000????1001000-

Tanycolagreus_topwilsoni ??1??000?00?00????????????????????????????1?0000???0??????????00000-1??12201?00?????????0?00?110?1?????????????000?010??????????????????????????????????????????????????????????????????????????????????????1000????101??01??0?10??0??000?0????????????011???1??0-??1??????????????0?0?00100????1001100?001?0?0?????????00?0???????20?00????1?????????????00010?1?10000??101110?011?0111000000010??11000010100?110002210?20?100000000??????????????????????????????????111101?1????0??????????????????10101100011000100100000?010111?00100011000100?2000111100100010?0?00010?0002001000-

Tarbosaurus_bataar ??0???00000011010000?100110-000000??0000????110010100120110011000?111101???100001??01000?-0?1010?112?111011001010??01031000010000?000??01?10010?1?000001001??0000-??????0-?????0??0011?00-?0000-00-100001?11110111001010101000010200??0?0000000?00010???????????????????????????????????????????????????????0????????????????????????????????????????????????????0???????????0????????????????????????????0?????????32????????????????????????????????????????????????????????????????????????????????????????????????????????????????????????????????????????1?????1??????1??????000?0-

Tawa_hallae ??0001201000??00----01001?11000?00000?1????0?000?0011?200?001000010-0--12100--?1100??0????000?0-00?0?0-??11010000??0????????????0?020?001???000??????000??1??0??????1?????????????10??000-00000-00-100?00-?0????0????????00??00002000?0000?0000?000100-0???????1100?00??00?1?????0?0????????????1????1???0???????0???????0??00????0?0?00????????????????????????1?10100?0100110000????????????0??1????000??10??00?01110001110?000000000000-0???0--?0?00??00000100?000?0110????????10?????????????????????001??2000000??100????????????????????????0001000000000?010-?00?00???00?1???????

Torvosaurus_tanneri ??001010???????0-?????????1100????00000101??????????1?2??10000???0101??1100???00???????????0010-00?????????????0?1?0?0????????????????????????????????????????????????????????????????0?0-???????0-??????????????????????00??0??0?0001?00?0????1????010000???1?10-1000?1?1?11????10002?10100????11000??1?0??03??????????1?02??0?0??0?1??????11?0??????????0??0-0000??????2?0??0201?00?0200?1?01001?????01111?0?10?01?????0????10????00100110??0????00?0?000000?????000?0-00???0000000100?10?00-1?0?0-???0??1????0?010?0??0??0??00111???0?0????00???011000111?????????????01?0???????????

Troodon_formosus ??0???001?0?0?010?011100?10-0000?1??0??1??????00???0????????00????110--????100??0-?00?00?01??00-?01??0-0???0?0?1???01030??1?11??0?0011000???001?010?1000001???010-1110-10-10?111??1?1??010?100??0110-00????????0????????101?00?00?201?0?1000220?001010-????111?10-111??210110?01?10?100101??????1??1011??0?10501?10?0????011?0?0?20?0??1?????????2??????????????1?00110???0?1?0?10??01?0????????0????000010?0???????220??????001?????????0-???????????????????????????010??0001???0??11??23??1?21000-0?0001?11120110?0?1???0?00110101??1????1?0100012000110?001?????100111?1?011?1??010-

Tsaagan_mangas ??00000020000?011010?100110-000000010001???00000100?1?0001000101010-0--1100?????0-11100001100?0-001200-000010?000?00103????0?000000100101001000?11010101100???0000??10110-1000000?001?000-00000-00-0-0?010011101011111?010101?0002101?0?1000000?000100-??????????????00?0?????110??????????????????????????????????????????????????????????????????????????????????????1????????????????????????????????????????????????????????????????????????????????????????????????????????????????????????????????????????????????????????????????????????????????????????????????????????????????

Tyrannosaurus_rex ??10?0000000110101011100110-00000001000001011101101000201100110001111101110110?1111010000-011?1001120111011001010100103?00001000000100101?10?00?100000010010-0?010?010-?0-100000000011000-00000-00-1000010111?011100101010100001020000000000000000010100000111010-11?00000011?00010001000000000010000001001003100000?0100000100000020000000-100110???101??0001001010000001100010012?00010001001001?0?0000101000----03200021--00000?00010010101110000000?1200011000000101011000101000010000000102100101011012001210010001000100010111?0010?0??0?0100020001101001?0011100001-110002000000-

Unenlagia_comahuensis ???????????????????????????????????????????????????????????????????????????????????????????????????????????????????????????????????????????????????????????????????????????????????????????????????????????????????????????????????????????????????????????????????????????????????0?20?1100????10011011011??4000?01?0??????????????????????10??????????????????1?00110?010011021????????????????????????????????????????????????????01000-2100101001110001000101011101110101011-010?1110210011????110100010010?1110000100?????????????1????????????????????????????????????????????????

Velociraptor_mongoliensis ??00000020000?0111101100110-000000110001???000001000110000?00101010-0--1120100?10-11101100100?0-000?0???00?10?000?0010?1?1?0100?00??0010?001?00?1?01001?000??0?00-??11110-100000010011000-00000-00-0-000100?1?0?0111101010?110000?00110010000000000100-0??111?010-???012000????10??01???1?00??0?1001011?01?00501010?0010001??010?1????01?110??1?120011001020110??100?111010?1?0??01?0010??1100??0?1?10000???010???0?220002?2?0010000001000-110011100101?00?1000-0110002110?01011-0001111022001021010-0111??1?11?11?000010000010?1???100???0????0000?20001???101?0011110010001111?001010-

Zanabazar_junior ??10000011000001010101001110000001110001????00001000??1???1????????????????111?????00?00?0100?0-00120100?0000?0???????????????????0011000????01?0??110001010-0010-??10-???0-?1110???0?001000000-0110-000???????????1100?1010001102101-0010001000101010-0?????????????????????????????????????????????????????401010?00???0??00???2-?0?????????1?12?????????????????????????????????????????????????????????????????????????????????????????????????????????????????????????????????????1??????0?????????????????????????????????????????????????????20001?????1???????????-?1???????????

Zuolong_salleei ??00??00?00?010101001?0?0?0-0000001110000000??????00???????0??010?0-0--1?10111011????00?0?00000-0?1100-00?100000001011300??0????0???????????????????????????????????????????????????????????????????????????????????????10100001020?0000100???????010100???????10-0?101100011??0?10??00?01???????????????????40001?000??000000??0?00????????????????????????????1?1?1???010001???010???????00?000???????????????????????????????0???00100??????????00?0???0???100?0000011?0010112?00???????????????????1?011000100001001000001010111?0???011??000????????????010????100010101000????0???

Santanaraptor_placidus ????????????????????????????????????????????????????????????????????????????????????????????????????????????????????????????????????????????????????????????????????????????????????????????????????????????????????????????????????????????????????????????????????????????????????????????????????????????????????????????0????0100????????????2???????????????????????????????????????????????????????????????????????????????????????????????????????????????????????????????????100000?0?01100100?110110001100110010000000111110001?????????00120001101?0100???1????01??00??0??0???

Timimus_hermani ????????????????????????????????????????????????????????????????????????????????????????????????????????????????????????????????????????????????????????????????????????????????????????????????????????????????????????????????????????????????????????????????????????????????????????????????????????????????????????????????????????????????????????????????????????????????????????????????????????????????????????????????????????????????????????????????????????????????????????????????????????101200121001100100??????????????????????????????????????????????????????????????

Bicentenaria_argentina ????????????????????????????????????????????????????00????10?00001???????????????????????????????????????00??0?1?110113???????????00111?1??????????????????????????????????????????????????????????????0?01???00???????0?0???0110?????0??0????????????????????????????????????????????????????????????????????010???????001?0???0????????????????????????????????????00?????????????00?0???????????????????????????????????????????????????????????00?????????100???????????????????????????????????????101100010000100100?00????????????????????????0??????????????????????????????0???

Aratasaurus_museunacionali ?????????????????????????????????????????????????????????????????????????????????????????????????????????????????????????????????????????????????????????????????????????????????????????????????????????????????????????????????????????????????????????????????????????????????????????????????????????????????????????????????????????????????????????????????????????????????????????????????????????????????????????????????????????????????????????????????????????????????????????????????????????????????????0?10?0001010111?0??????????????????????00?000?1?00?1??01?0??000000-

;

END;

BEGIN ASSUMPTIONS;

TYPESET * UNTITLED = unord: 1 - 73 75 - 81 83 - 99 101 - 118 120 - 124 126 - 131 133 - 149 151 - 178 180 - 182 184 - 218 220 - 225 228 - 232 234 - 235 238 - 262 265 - 271 274 - 277 279 - 281 283 - 301 304 - 310 312 - 318 320 - 321 323 - 326\3 327 - 337 339 - 346 348 - 361 363 - 365 367 369 - 370 372 - 375 377 - 404 407 - 409 411 413 - 416 418 420 422 - 441 443 445 - 454 456 - 478 480 482 - 493 495 497 - 532 534 - 553 555 - 560 562 - 568, ord: 74 82 100 119 125 132 150 179 183 219 226 - 227 233 - 236\3 237 263 - 264 272 - 273 278 282 302 - 303 311 319 - 322\3 324 - 325 338 347 362 366 368 - 371\3 376 405 - 406 410 412 417 419 421 442 444 455 479 481 494 496 533 554 561;

EXSET * UNTITLED = ;

WTSET * UNTITLED = 1: 1 - 568 ;

END;

BEGIN NOTES;

TEXT TAXON = 29 CHARACTER = 7 TEXT = 'Angle appears greater than 75 degrees, but body is longer in front of naris';

TEXT TAXON = 29 CHARACTER = 9 TEXT = Difficult_to_determine_from_article;

TEXT TAXON = 35 CHARACTER = 14 TEXT = 'This area is damaged on the right side, but the left side doesn''t reveal any evidence of a snf';

TEXT TAXON = 43 CHARACTER = 14 TEXT = 'Benson, 2009 considers it absent, but it is present on the paratype';

TEXT TAXON = 48 CHARACTER = 14 TEXT = 'I tentatively score this character as present, as there is a small foramen on the maxillary posterior process of the premaxilla.';

TEXT TAXON = 60 CHARACTER = 14 TEXT = 'Benson, 2009 considers it absent, but I saw it on the holotype';

TEXT TAXON = 36 CHARACTER = 16 TEXT = It_appears_that_a_maxillary_fenestra_is_present_on_the_right_maxilla_of_the_mounted_specimen.;

TEXT TAXON = 93 CHARACTER = 18 TEXT = 'Here, I''ve scored it as posterior because on the medial surface there is a large promaxillary recess rostral to the opening of the maxillary fenestra^n^n';

TEXT TAXON = 16 CHARACTER = 20 TEXT = This_is_labeled_ifc_on_the_Makovicky_et_al_2003_paper._Should_check_it_at_the_AMNH;

TEXT TAXON = 76 CHARACTER = 20 TEXT = 'This scoring very tentative - an embayment is described in this region by Kobayashi et al, but no illustration is presented. The specimen is not adequately prepared to score this character.';

TEXT TAXON = 79 CHARACTER = 20 TEXT = 'Athough Xu and Wu reconstruct the skull without this foramen, the specimen appears to be damaged in this region, so is scored as uncertain';

TEXT TAXON = 53 CHARACTER = 21 TEXT = 'Carrano, Sampson and Forster only mention the possibility of the homologous condition to a true promaxillary fenestra.';

TEXT TAXON = 97 CHARACTER = 21 TEXT = 'While Clark does not consider the depression to be a fenestra promaxillaris, I do. Talk to Jim to get his opinion';

TEXT TAXON = 44 CHARACTER = 27 TEXT = 'Must check on this state for Shuvuuia, as the maxilla does have an offset, although it is dorsally and posteriorly displaced';

TEXT TAXON = 23 CHARACTER = 30 TEXT = 'Benson, 2009 incorrectly codes this as pointed';

TEXT TAXON = 93 CHARACTER = 34 TEXT = Must_measure_this;

TEXT TAXON = 37 CHARACTER = 35 TEXT = 'Zanno et al, 2009 has the maxilla labeled backwards, so that the medial labels appear on the lateral picture and vice versa';

TEXT TAXON = 67 CHARACTER = 37 TEXT = 'An incipient ridge is present, but it is very weakly developed';

TEXT TAXON = 43 CHARACTER = 45 TEXT = 'Benson, 2009 codes this character as unfused for Guanlong, but the dorsal surface is fused in the larger skull, with a ventral suture visible. A similar situation is present in Eotyrannus, so I''m keeping it coded as fused.';

TEXT TAXON = 82 CHARACTER = 56 TEXT = 'Benson, 2009 notes that there is a ventral process under the antorbital fossa, but I haven''t seen this so I''ve kept it as state 0';

TEXT TAXON = 11 CHARACTER = 64 TEXT = A_small_posterior_process_is_present;

TEXT TAXON = 69 CHARACTER = 66 TEXT = 'The articular facet on the jugal is only slightly anterior to the infratemporal fenestra, but the anterior tip of the quadratojugal lies posterior to posterior margin';

TEXT TAXON = 44 CHARACTER = 69 TEXT = Check_this_on_the_CT_data;

TEXT TAXON = 77 CHARACTER = 69 TEXT = 'There does seem to be a slit-like foramen opening beneath the lateral lacrimal fold, but this is full of matrix in IGM 100/0977 and is not preserved adequately in IGM 100/1001';

TEXT TAXON = 3 CHARACTER = 70 TEXT = 'Although three foramena exist in this region (Eddy, 2008), only the largest appears to be a true lacrimal pneumatic recess.';

TEXT TAXON = 26 CHARACTER = 70 TEXT = 'Smith et al 2007 don''t consider this a lacrimal foramen, but it is developed in the same place as that of eustrptospondylus.';

TEXT TAXON = 17 CHARACTER = 76 TEXT = 'Although there is a posterodorsal process, witmer et al 2007 identify this as a fused prefrontal, a likely scenario given the rugose skull';

TEXT TAXON = 77 CHARACTER = 76 TEXT = It_is_listed_as_present_in_Chiappe_et_al_2002;

TEXT TAXON = 6 CHARACTER = 82 TEXT = 'Here the prefrontal only contributes slightly to the orbital margin so I''ve scored it a 1, double check this at the USNM';

TEXT TAXON = 21 CHARACTER = 86 TEXT = 'The postorbital forms the majority of the orbital margin, unlike most other theropods, but there is a clear groove on the lateral surface of the frontal that articulates with the frontal process of the postorbital';

TEXT TAXON = 23 CHARACTER = 86 TEXT = 'Scored from Makovicky, personal communication in reference to Makovicky and Turner 2008';

TEXT TAXON = 62 CHARACTER = 86 TEXT = Suggestion_of_a_groove_is_present_on_both_sides_of_the_skull_but_scoring_is_provisional_due_to_damage;

TEXT TAXON = 23 CHARACTER = 100 TEXT = scored_from_Peyer_2006;

TEXT TAXON = 91 CHARACTER = 112 TEXT = 'MOR 116 from the Judith river is an isolated broken right quadrate and the ventral portion reveals a completely hollow quadrate. Also, a pneumatic foramen exists in the medial side of the quadrate.';

TEXT TAXON = 91 CHARACTER = 118 TEXT = MOR_116_and_170_clearly_show_a_quadrate_foramen_opening_between_the_quadrate_and_quadratojugal.;

TEXT TAXON = 43 CHARACTER = 120 TEXT = 'There is a hole in the dorsal surface of the ectopterygoid, but I interpret this as a preservational artifact.';

TEXT TAXON = 77 CHARACTER = 134 TEXT = 'The dorsal recess on the basipterygoid makes them hollow, but this differs from the inflated condition present in derived troodontids, Chirostenotes and ornithomimosaurs';

TEXT TAXON = 82 CHARACTER = 142 TEXT = Another_instance_where_the_basisphenoid_is_oriented_almost_at_a_45_degree_angle_to_the_horizontal_plane.;

TEXT TAXON = 86 CHARACTER = 142 TEXT = 'Interestingly, the basisphenoid of syntarsus is mor vertically oreiented than in many coelurosaurs. Might have to change the states to account for this.';

TEXT TAXON = 43 CHARACTER = 145 TEXT = 'This really isn''t a basisphenoid depression at all, as it''s contained within the subcondylar recess which is clearly formed by the union of the basioccipital and the exoccipital/opisthotic';

TEXT TAXON = 19 CHARACTER = 150 TEXT = 'Benson codes them as straight, but Madsen and Welles show them as ventrolaterally projecting';

TEXT TAXON = 2 CHARACTER = 154 TEXT = Tentative_scoring;

TEXT TAXON = 67 CHARACTER = 159 TEXT = 'Although the basal tubera are clearly visible, they are not fused and I can''t sort this character out.';

TEXT TAXON = 6 CHARACTER = 160 TEXT = 'Benson, 2009 codes them as narrowly separated, but they are clearly outside the lateral condylar margins in Madsen, 1979';

TEXT TAXON = 82 CHARACTER = 160 TEXT = 'Benson codes them as narrowly separated, but they are clearly lateral to the occipital condyle';

TEXT TAXON = 68 CHARACTER = 166 TEXT = 'Rauhut 2004 figures a dorsal depression, but it isn''t developed as a pneumatic recess as in the braincase of troodontids';

TEXT TAXON = 67 CHARACTER = 179 TEXT = The_mandibular_foramen_looks_large_in_the_sense_that_it_stretches_from_the_posterior_end_of_the_surangular_below_the_postorbital_anteriorly_to_a_point_just_ventral_to_the_lacrimal.;

TEXT TAXON = 91 CHARACTER = 181 TEXT = 'The interdental plates seem tightly fused to the dentary and there are individual foramina between the plates, but there does seem to be some plate development in MOR 563 and 553, particularly at the anterior end of the dentary.';

TEXT TAXON = 8 CHARACTER = 184 TEXT = 'HERE THERIZINOSAUROIDS ONLY HAVE THE CONVEXITY BECAUSE OF THE SYMPHYSEAL DOWNTURN, THAT IS THERE''S NO DORSAL EMINENCE SO THEY GET A 0';

TEXT TAXON = 67 CHARACTER = 188 TEXT = 'The lower jaw of the holotype is close against the maxilla and there is a slight downward deflection of the anterior end so that there is a gap between the premaxilla and the dentary that isn''t present between the maxilla and the dentary';

TEXT TAXON = 43 CHARACTER = 194 TEXT = 'Though there is a small groove on the labial surface of the left dentary in the smaller specimen, it is very shallow.';

TEXT TAXON = 51 CHARACTER = 201 TEXT = 'A foramen is present,but it is in a very anterior position, which we tentatively homologize with the more posteriorly positioned surangular foramina of other theropods';

TEXT TAXON = 67 CHARACTER = 203 TEXT = 'The presence of this ridge is confirmed on the left side of the skull by a poorly developed lateral flange just dorsal to the surangular foramen and a corresponding lateral flange on the anterior end of the surangular near the dentary contact. In between, the ridge is covered by the jugal laterally.';

TEXT TAXON = 43 CHARACTER = 207 TEXT = retroarticular_process_is_broken_in_the_smaller_specimen;

TEXT TAXON = 6 CHARACTER = 214 TEXT = 'Benson, 2009 codes it as open, but it appears that the element may be damaged and the foramen is fully enclosed';

TEXT TAXON = 27 CHARACTER = 215 TEXT = 'I''m questioning what exactly constitutes "forked"';

TEXT TAXON = 67 CHARACTER = 219 TEXT = There_are_six;

TEXT TAXON = 97 CHARACTER = 221 TEXT = 'Based on the size of the root, all premaxillary teeth are of equal size';

TEXT TAXON = 43 CHARACTER = 229 TEXT = 'The anterior two maxillary teeth have both anterior and posterior carinae, but the more posterior teeth are missing the mesial (anterior) carina, as in Proceratosaurus';

TEXT TAXON = 67 CHARACTER = 231 TEXT = 'Amazingly, the teeth resemble those of Falcarius closely, but they don''t have serrations';

TEXT TAXON = 53 CHARACTER = 238 TEXT = 'This character diagnostic of Masiakasaurus - procumbent anterior maxillary teeth with carinae that have been rotated 90 degrees out of their usual plane';

TEXT TAXON = 53 CHARACTER = 239 TEXT = 'Anterior teeth are spatulate, posterior teeth are mediolaterally flattened and recurved';

TEXT TAXON = 22 CHARACTER = 248 TEXT = 'These are figured in Colbert, 1989, Fig 46B p71';

TEXT TAXON = 69 CHARACTER = 248 TEXT = 'Although striations are present on some maxillary teeth (Rauhut et al, 2009), they do not resemble those of spinosaurids, being rounded and larger';

TEXT TAXON = 6 CHARACTER = 251 TEXT = 'Rauhut and Kirkland et al disagree here - Rauhut considers the neural spine of the axis to be cylindrical and mediolaterally compressed. Based on Madsen 1976, I agree with Kirkland et al that the neural spine is transversely flared.';

TEXT TAXON = 57 CHARACTER = 251 TEXT = 'Again, Rauhut and Kirkland et al disagree. Further study needed';

TEXT TAXON = 7 CHARACTER = 256 TEXT = 'Here I disagree with Novas over whether Alvarezsaurus has a cervical centrum with pleurocoels - the cervical centrum i found in the collection doesn''t have them, but isn''t very similar to what Novas 1996 figures.';

TEXT TAXON = 45 CHARACTER = 263 TEXT = 'The cervical vertebrae have been greatly compressed mediolaterally during preservation, but they are very long, and probably in the area of 3-5 times as long as wide.';

TEXT TAXON = 83 CHARACTER = 264 TEXT = Crushing_in_the_specimen_and_mediolateral_deformation_preclude_the_assessment_of_this_character;

TEXT TAXON = 23 CHARACTER = 266 TEXT = 'Peyer 2006 says that compsognathus has opisthocoelus cervical centra, but this really isn''t true, especially compared to the markedly opisthocoelus centra of the alvarezsaurids';

TEXT TAXON = 29 CHARACTER = 266 TEXT = 'Make sure to check this character state, as Peyer considered it synapomorphic of the Alvarezsaurs';

TEXT TAXON = 97 CHARACTER = 266 TEXT = 'Here the anterior articular surface is flat or very slightly rounded anteriorly, but the posterior articulation is deeply excavated.';

TEXT TAXON = 45 CHARACTER = 271 TEXT = 'Cervicals only presented in lateral view, discussion is limited and they are badly crushed';

TEXT TAXON = 67 CHARACTER = 271 TEXT = Inspection_of_the_right_side_of_the_skull_block_reveals_that_an_extensive_connection_between_the_postzygapophyses_was_likely_present;

TEXT TAXON = 76 CHARACTER = 271 TEXT = Does_not_have_cervicals;

TEXT TAXON = 7 CHARACTER = 277 TEXT = 'A hypapophysis does appear to be present, if weakly developed, on the isolated centrum. Not sure of its position in the cervical series, but it appears to be anterior.';

TEXT TAXON = 66 CHARACTER = 278 TEXT = Chiappe_et_al_2003_say_that_the_dorsal_pleurocoels_are_absent_in_all_alvarezsaurids;

TEXT TAXON = 6 CHARACTER = 280 TEXT = 'Benson, 2009 codes this as weak or absent but I disagree';

TEXT TAXON = 94 CHARACTER = 284 TEXT = 'The most posterior neural spine is ever so slightly anteriorly oriented, but not the derived condition for sure';

TEXT TAXON = 43 CHARACTER = 292 TEXT = 'The anterior dorsals have separated hyposphenes, while the posterior dorsals have hyposphenes that meet to form a lamina.';

TEXT TAXON = 94 CHARACTER = 293 TEXT = This_expansion_is_slight_but_present;

TEXT TAXON = 43 CHARACTER = 295 TEXT = 'On the posterior surface of the neural spine, they terminate close to the apex, and on the anterior surface, well below the apex.';

TEXT TAXON = 62 CHARACTER = 295 TEXT = 'Or so say Carpenter, Miles, Ostrom and Cloward, 2005';

TEXT TAXON = 44 CHARACTER = 299 TEXT = 'here, the posteriormost parapophyses are just a small bit more ventrally located than the diapophyseal facets';

TEXT TAXON = 44 CHARACTER = 300 TEXT = 'very tentative scoring - I don''t know this character very well';

TEXT TAXON = 7 CHARACTER = 302 TEXT = 'This is speculative, but based on the divergence of the postacetabular ala and the position of the other sacral vertebrae, Alvarezsaurus definitely had at least four, likely five, and probably not six.';

TEXT TAXON = 23 CHARACTER = 303 TEXT = 'Peyer 2006: there may be a small pleurocoel close to sutural boundary with neural arch';

TEXT TAXON = 7 CHARACTER = 304 TEXT = 'There is a keel on the last preserved vertebrae, but it isn''t as well-developed as in Shuvuuia. The middle sacrals are ventrally grooved and the anterior sacral is convex ventrally.';

TEXT TAXON = 7 CHARACTER = 307 TEXT = 'It''s possible here that Bonaparte got the pelvis backwards on the initial description - double check this. Should really be marked as a question mark.';

TEXT TAXON = 7 CHARACTER = 315 TEXT = 'This coding taken from Chiappe, but the holotype MUCPv 54 no longer includes this material. There are two partially articulated vertebrae that seem to match the description, though, and they are ventrally keeled, although they don''t have the ridiculous condition that is present in Shuvuuia. ';

TEXT TAXON = 77 CHARACTER = 330 TEXT = 'I''m pretty sure they''re present on IGM 100/0977';

TEXT TAXON = 66 CHARACTER = 350 TEXT = 'There is no coracoid tuber in Patagonykus, but like Haplocheirus, the posteroventral blade features a triangular low ridge on the lateral surface that extends for the length of the blade and terminates just posteroventral to the coracoid foramen. This is not well-developed in Alvarezsaurus, but little of the coracoid is preserved in that taxon. Makovicky calls this the subglenoid fossa, and it is well-developed in ornithomimosaurs and also in Nqwebasaurus.';

TEXT TAXON = 23 CHARACTER = 355 TEXT = 'Here, Rauhut and Kirkland et al disagree. Further research necessary';

TEXT TAXON = 87 CHARACTER = 355 TEXT = 'Benson, 2009 codes this as a 0 but he is wrong';

TEXT TAXON = 66 CHARACTER = 360 TEXT = 'Turner has this one wrong, the coracoid is clearly inflected medially in Novas 1997, figure 145';

TEXT TAXON = 86 CHARACTER = 368 TEXT = 'A small notch separates the internal tuberosity, which is located well distal to the humeral head.';

TEXT TAXON = 6 CHARACTER = 373 TEXT = 'the entepicondyle of Allosaurus is very weakly developed, contrary to Benson, 2009';

TEXT TAXON = 90 CHARACTER = 380 TEXT = 'Benson, 2009 codes this as absent, but from the Galton and Jensen, 1979 publication it is clearly present';

TEXT TAXON = 45 CHARACTER = 388 TEXT = The_distal_carpal_bears_a_shallow_trochlea_proximally;

TEXT TAXON = 66 CHARACTER = 390 TEXT = There_is_no_evidence_for_fusion_of_the_distal_carpals_into_the_carpometacarpus.;

TEXT TAXON = 2 CHARACTER = 392 TEXT = 'Although the first metacarpal is still much shorter than the second metacarpal, the ratio of length I to II is more than 0.5, scored this as 0 to homologize the condition with Harpymimus';

TEXT TAXON = 18 CHARACTER = 393 TEXT = Here_Rauhut_and_Kirkland_et_al_disagree.__Do_further_research;

TEXT TAXON = 66 CHARACTER = 394 TEXT = There_is_a_clear_surface_on_the_proximal_half_of_the_lateral_surface_of_McI_for_articulation_with_the_second_metacarpal._The_lateral_surface_of_the_distal_condyle_also_bears_a_small_flattened_area_that_suggests_it_contacted_the_distal_portion_of_McII.;

TEXT TAXON = 44 CHARACTER = 407 TEXT = 'Haplocheirus has a single ventral proximal tubercle that is similar to the condition present in Falcarius, where the tubercle is offset.';

TEXT TAXON = 66 CHARACTER = 408 TEXT = 'The ventral surface does bear an axial furrow, but the median portion of this furrow has a small, X-shaped area where two ridges cross each other and form a raised area with two lateral foramina. This is best developed in PVPH 102';

TEXT TAXON = 66 CHARACTER = 417 TEXT = Coded_from_PVPH_102;

TEXT TAXON = 94 CHARACTER = 433 TEXT = 'The anterior end of the fossa has a well-developed lateral overhang of the preacetabular hook, but the posterior end has no overhang and is laterally directed';

TEXT TAXON = 83 CHARACTER = 435 TEXT = Crushing_in_the_specimen_and_mediolateral_deformation_preclude_the_assessment_of_this_character;

TEXT TAXON = 6 CHARACTER = 436 TEXT = Here_Rauhut_and_Kirkland_et_al_disagree.__Further_research;

TEXT TAXON = 7 CHARACTER = 436 TEXT = 'Looking at the holotype MUCPv 54 clearly shows that the brevis fossa is mediolaterally broad. This character should be revised, it''s unclear what is meant by lateral overhang.';

TEXT TAXON = 43 CHARACTER = 436 TEXT = 'The brevis shelf is unusually wide at the distal end and funnel shaped, though there isn''t any lateral expansion as in ornithomimosauria';

TEXT TAXON = 62 CHARACTER = 436 TEXT = Here_Rauhut_and_Kirkland_disagree;

TEXT TAXON = 1 CHARACTER = 437 TEXT = 'It''s uncertain whether herrarasaurus has a brevis fossa, however, a distinct muscle attachment "furrow" is visible in the same position, therefore I regard it as homologous';

TEXT TAXON = 58 CHARACTER = 445 TEXT = 'Benson codes this as facing anteriorly, but I disagree';

TEXT TAXON = 74 CHARACTER = 448 TEXT = 'A very small ridge-like crest extends from the posteroventral base of the pubic peduncle along the rim of the acetabulum, ending approximately 2/3 of the way to the ischial peduncle.';

TEXT TAXON = 94 CHARACTER = 448 TEXT = State_0_edited_by_JNC;

TEXT TAXON = 83 CHARACTER = 450 TEXT = Crushing_in_the_specimen_and_mediolateral_deformation_preclude_the_assessment_of_this_character;

TEXT TAXON = 74 CHARACTER = 451 TEXT = 'The tuber is present, but isn''t as pronounced as in adasaurus mongoliensis. Tuber is directly above ischial peduncle.';

TEXT TAXON = 94 CHARACTER = 452 TEXT = 'This posterior extension of the brevis shelf is a very subtle feature, but it is present';

TEXT TAXON = 94 CHARACTER = 458 TEXT = 'It''s hard to tell because the distal end of the pubic boot still has matrix adhering to its surface and this the length of the boot may be exaggerated.';

TEXT TAXON = 66 CHARACTER = 459 TEXT = 'Novas got the identification wrong, it''s actually the left pubis.';

TEXT TAXON = 83 CHARACTER = 465 TEXT = 'Probably a foramen, there is broken bone surrounding the rim and the pubic apron is poorly developed that high up so it can''t be a major contribution of the pubic apron.';

TEXT TAXON = 66 CHARACTER = 468 TEXT = 'Not to be confused with the more proximally located pubic tubercle, which is the attachment for m. ambiens and m. rectus abdominis.';

TEXT TAXON = 94 CHARACTER = 469 TEXT = 'This really should be a question mark - the distal end of the ischium is broken.';

TEXT TAXON = 83 CHARACTER = 474 TEXT = The_distal_ends_of_both_ischia_abandon_the_straight_trajectory_of_the_shaft_and_curve_ventrally.;

TEXT TAXON = 23 CHARACTER = 477 TEXT = Benson_codes_this_as_present_but_Peyer_2006_figures_it_as_absent;

TEXT TAXON = 30 CHARACTER = 477 TEXT = 'Notch is present figured by Welles, 1984 not absent as in Benson, 2009';

TEXT TAXON = 44 CHARACTER = 477 TEXT = 'Unfortunately, this notch is very different in form from the notch in Allosaurus and it''s probably not homologous. Refine the states';

TEXT TAXON = 18 CHARACTER = 478 TEXT = Rauhut_and_Kirkland_et_al_disagree_here.__Further_research_required;

TEXT TAXON = 62 CHARACTER = 480 TEXT = 'Here Rauhut and Kirkland et al disagree. More research needed. (Looking at the AMNH #619 specimen shows almost no distal expansion of the ischium, hence state 0)^n^n';

TEXT TAXON = 83 CHARACTER = 486 TEXT = 'The semicircular scar is a rugose patch in tyrannosaurus, since it gets a ''present'' I give the mildly rugose patch in Stokesosaurus a 1';

TEXT TAXON = 53 CHARACTER = 488 TEXT = Take_a_look_at_the_proximal__sulcus_with_Jim.__It_appears_that_this_sulcus_is_a_fovea_capitalis.;

TEXT TAXON = 94 CHARACTER = 489 TEXT = A_groove_in_this_postion_actually_appears_to_be_insect_damage_postmortem;

TEXT TAXON = 18 CHARACTER = 495 TEXT = 'Here, Rauhut and Kirkland disagree again on the shape of the lesser trochanter. Further research necessary';

TEXT TAXON = 57 CHARACTER = 495 TEXT = Kirkland_and_Rauhut_disagree_again;

TEXT TAXON = 94 CHARACTER = 496 TEXT = 'No way to tell this on PVPH 78, the proximal end of the lesser trochanter is broken';

TEXT TAXON = 43 CHARACTER = 497 TEXT = 'The lesser trochanter has a novel shape to it, consisting of two bulges, one located more proximally and one more distally';

TEXT TAXON = 50 CHARACTER = 497 TEXT = 'Turner et al, 2007 label this as lr? in their Figure 2';

TEXT TAXON = 53 CHARACTER = 497 TEXT = 'Is this the same as the trochanteric shelf of Hutchinson, 2001?';

TEXT TAXON = 7 CHARACTER = 498 TEXT = There_is_a_distinct_low_swelling_on_both_proximal_femora_of_the_holotype_MUCPv_54;

TEXT TAXON = 94 CHARACTER = 498 TEXT = 'A distinct ridge is present here, and adjacent is a large, hollowed out area but this appears to be damage to the specimen';

TEXT TAXON = 7 CHARACTER = 499 TEXT = 'The slightest suggestion of a low ridge is present in this position on the right femur of the holotype, and it is even weaker on the left femur of the holotype.';

TEXT TAXON = 87 CHARACTER = 499 TEXT = 'Benson codes it as absent, it''s clearly present';

TEXT TAXON = 83 CHARACTER = 500 TEXT = There_is_at_least_a_suggestion_of_the_presence_of_this_groove_on_both_sides.;

TEXT TAXON = 66 CHARACTER = 510 TEXT = 'It appears from the photographs that the medial cnemial crest of the tibia in parvicursorines may actually pertain to the cnemial crest, and that the lateral of the two pertains to the laterally-developed cnemial crest because in Patagonykus the lateral cnemial crest is very proximal and looks like an incipient version of parvicursorines.';

TEXT TAXON = 66 CHARACTER = 511 TEXT = Patagonykus_has_an_incipient_lateral_cnemial_crest_developed_as_a_triangular_tuber_immediately_anterior_to_the_fibular_condyle.;

TEXT TAXON = 65 CHARACTER = 512 TEXT = 'The incisurua tibialis is very shallow, as in Eustreptospondylus';

TEXT TAXON = 82 CHARACTER = 512 TEXT = 'Benson, 2009 has this coded wrong';

TEXT TAXON = 3 CHARACTER = 513 TEXT = 'this character is the same as Benson, 2009 #201';

TEXT TAXON = 60 CHARACTER = 513 TEXT = 'Benson codes this as bulbous, but it''s arcuate in my opinion';

TEXT TAXON = 53 CHARACTER = 514 TEXT = 'Fibular condyle of Rauhut = lateral condyle of other others';

TEXT TAXON = 7 CHARACTER = 520 TEXT = 'Proximal to the flattened surface for articulation with the ascending process, there is what appears to be a medially positioned, short grooved extending proximally. Breakage of the specimen in this region, however, makes it hard to determine whether this feature is real. It would be an interesting homology with Farragochela. ';

TEXT TAXON = 74 CHARACTER = 525 TEXT = 'Clearly defined tubercle that Rauhut illustrates well in Figure 48 (2003)^n^n';

TEXT TAXON = 7 CHARACTER = 530 TEXT = 'The groove is obliquely oriented, but is present';

TEXT TAXON = 74 CHARACTER = 532 TEXT = The_fibular_facet_is_a_small_deep_pocket_that_faces_proximately_and_laterally_and_posteriorly.__Here_i_code_it_as_reduced.;

TEXT TAXON = 66 CHARACTER = 539 TEXT = 'A slight suggestion of a groove is present on the right astragalar medial condyle, but I''ve elected to score it as absent because the contralateral side does not preserve any groove.';

TEXT TAXON = 74 CHARACTER = 540 TEXT = 'It appears to me that the calcaneum and astragalus are fused. If this is true, the calcaneum merely forms the lateral edge of the fibular facet, without being excavated or emarginated, so it doesn''t have a true facet.^n^n';

TEXT TAXON = 18 CHARACTER = 554 TEXT = 'Here, rauhut and Kirkland et al disagree. Research further';

TEXT TAXON = 7 CHARACTER = 562 TEXT = 'This is impossible to tell - Bonaparte''s reconstruction infers a lot about phalanges that really can''t be figured out without a complete preparation of the specimen.';

TEXT TAXON = 2 CHARACTER = 563 TEXT = Need_to_get_Billy_to_confirm_this_scoring;

END;

BEGIN MESQUITECHARMODELS;

ProbModelSet * UNTITLED = 'Mk1 (est.)': 1 - 568;

END;

Begin MESQUITE;

MESQUITESCRIPTVERSION 2;

TITLE AUTO;

tell ProjectCoordinator;

timeSaved 1588198224951;

getEmployee #mesquite.minimal.ManageTaxa.ManageTaxa;

tell It;

setID 0 6496623979605601020;

tell It;

setDefaultOrder 0 1 2 3 4 5 6 7 8 9 10 11 12 13 14 15 16 17 18 19 20 21 22 23 24 25 26 27 28 29 30 31 32 33 34 35 36 37 38 39 40 41 42 43 44 45 46 47 48 49 50 51 52 53 54 55 56 57 58 59 60 61 62 63 64 65 66 67 68 69 70 71 72 73 74 75 76 77 78 79 80 81 82 83 84 85 86 87 88 89 90 91 92 93 94 95 96 98 101 99 97;

attachments ;

endTell;

endTell;

getEmployee #mesquite.charMatrices.ManageCharacters.ManageCharacters;

tell It;

setID 0 7487933091202431150;

mqVersion 340;

checksumv 0 3 2391357291 null getNumChars 568 numChars 568 getNumTaxa 101 numTaxa 101 short true bits 127 states 127 sumSquaresStatesOnly 82203.0 sumSquares 82203.0 longCompressibleToShort false usingShortMatrix true NumFiles 1 NumMatrices 1;

mqVersion;

endTell;

getWindow;

tell It;

suppress;

setResourcesState false false 100;

setPopoutState 400;

setExplanationSize 0;

setAnnotationSize 0;

setFontIncAnnot 0;

setFontIncExp 0;

setSize 1362 629;

setLocation -8 0;

setFont SanSerif;

setFontSize 10;

getToolPalette;

tell It;

endTell;

desuppress;

endTell;

getEmployee #mesquite.minimal.ManageTaxa.ManageTaxa;

tell It;

showTaxa #6496623979605601020 #mesquite.lists.TaxonList.TaxonList;

tell It;

setTaxa #6496623979605601020;

getWindow;

tell It;

useTargetValue off;

setTargetValue ;

newAssistant #mesquite.lists.TaxonListCurrPartition.TaxonListCurrPartition;

setExplanationSize 30;

setAnnotationSize 20;

setFontIncAnnot 0;

setFontIncExp 0;

setSize 1262 557;

setLocation -8 0;

setFont SanSerif;

setFontSize 10;

getToolPalette;

tell It;

setTool mesquite.lists.TaxonList.TaxonListWindow.arrow;

endTell;

endTell;

showWindow;

getEmployee #mesquite.lists.ColorTaxon.ColorTaxon;

tell It;

setColor Red;

removeColor off;

endTell;

getEmployee #mesquite.lists.TaxonListAnnotPanel.TaxonListAnnotPanel;

tell It;

togglePanel off;

endTell;

endTell;

endTell;

getEmployee #mesquite.charMatrices.BasicDataWindowCoord.BasicDataWindowCoord;

tell It;

showDataWindow #7487933091202431150 #mesquite.charMatrices.BasicDataWindowMaker.BasicDataWindowMaker;

tell It;

getWindow;

tell It;

setExplanationSize 30;

setAnnotationSize 20;

setFontIncAnnot 0;

setFontIncExp 0;

setSize 1262 557;

setLocation -8 0;

setFont SanSerif;

setFontSize 10;

getToolPalette;

tell It;

setTool mesquite.charMatrices.BasicDataWindowMaker.BasicDataWindow.ibeam;

endTell;

setActive;

setTool mesquite.charMatrices.BasicDataWindowMaker.BasicDataWindow.ibeam;

colorCells #mesquite.charMatrices.ColorCells.ColorCells;

tell It;

setColor Yellow;

removeColor off;

endTell;

colorRowNames #mesquite.charMatrices.TaxonGroupColor.TaxonGroupColor;

colorColumnNames #mesquite.charMatrices.CharGroupColor.CharGroupColor;

colorText #mesquite.charMatrices.NoColor.NoColor;

setBackground White;

toggleShowNames off;

toggleShowTaxonNames on;

toggleTight off;

toggleThinRows off;

toggleShowChanges on;

toggleSeparateLines off;

toggleShowStates on;

toggleAutoWCharNames on;

toggleAutoTaxonNames off;

toggleShowDefaultCharNames off;

toggleConstrainCW on;

toggleBirdsEye off;

toggleShowPaleGrid off;

toggleShowPaleCellColors off;

toggleShowPaleExcluded off;

togglePaleInapplicable on;

toggleShowBoldCellText off;

toggleAllowAutosize on;

toggleColorsPanel off;

toggleDiagonal on;

setDiagonalHeight 80;

toggleLinkedScrolling on;

toggleScrollLinkedTables off;

endTell;

showWindow;

getWindow;

tell It;

forceAutosize;

endTell;

getEmployee #mesquite.charMatrices.AlterData.AlterData;

tell It;

toggleBySubmenus off;

endTell;

getEmployee #mesquite.charMatrices.ColorByState.ColorByState;

tell It;

setStateLimit 9;

toggleUniformMaximum on;

endTell;

getEmployee #mesquite.categ.StateNamesStrip.StateNamesStrip;

tell It;

showStrip off;

endTell;

getEmployee #mesquite.charMatrices.AnnotPanel.AnnotPanel;

tell It;

togglePanel off;

endTell;

getEmployee #mesquite.charMatrices.CharReferenceStrip.CharReferenceStrip;

tell It;

showStrip off;

endTell;

getEmployee #mesquite.charMatrices.QuickKeySelector.QuickKeySelector;

tell It;

autotabOff;

endTell;

getEmployee #mesquite.charMatrices.SelSummaryStrip.SelSummaryStrip;

tell It;

showStrip off;

endTell;

getEmployee #mesquite.categ.SmallStateNamesEditor.SmallStateNamesEditor;

tell It;

panelOpen true;

endTell;

endTell;

endTell;

getEmployee #mesquite.charMatrices.ManageCharacters.ManageCharacters;

tell It;

showCharacters #7487933091202431150 #mesquite.lists.CharacterList.CharacterList;

tell It;

setData 0;

getWindow;

tell It;

useTargetValue off;

setTargetValue ;

newAssistant #mesquite.lists.DefaultCharOrder.DefaultCharOrder;

newAssistant #mesquite.lists.CharListInclusion.CharListInclusion;

newAssistant #mesquite.lists.CharListPartition.CharListPartition;

newAssistant #mesquite.stochchar.CharListProbModels.CharListProbModels;

newAssistant #mesquite.parsimony.CharListParsModels.CharListParsModels;

getTable;

tell It;

rowNamesWidth 817;

endTell;

setExplanationSize 30;

setAnnotationSize 20;

setFontIncAnnot 0;

setFontIncExp 0;

setSize 1262 557;

setLocation -8 0;

setFont SanSerif;

setFontSize 10;

getToolPalette;

tell It;

endTell;

endTell;

showWindow;

getEmployee #mesquite.lists.CharListAnnotPanel.CharListAnnotPanel;

tell It;

togglePanel off;

endTell;

endTell;

endTell;

endTell;

end;
