# Supplementary material for: The first theropod dinosaur (Coelurosauria, Theropoda) from the base of the Romualdo Formation (Albian), Araripe Basin, Northeast Brazil
Source: Sci Rep. 2020 Jul 10;10:10892. doi: 10.1038/s41598-020-67822-9 (PMC7351750; doi:10.1038/s41598-020-67822-9)
Supplement: Supplementary file 3 — Supplementary material 3: The coding matrix for nexus and TNT used in the present study. [file 41598_2020_67822_MOESM3_ESM.docx]

xread

568 101

Herrerasaurus_ischigualastensis ??000000?00??100----???0010-00000000000????0?00000001?2010000000000-0--01100--0010000???0-?00?0-00?00??00?1????01???11??000?0?0000?0????0???000???01?1?0?0?0-000????0???????????0???1?000-0?0011???????010?000??????????1010?000?010000000???0?000??00-0000100?0????0010?001000?0000000000000000100?0??1??0?00000???200000000?000?0000?????????00?????????0??0-00?1010?00110?1000010??0100?0?00001?0??010000?10000011000?11100000???0000?0-0??0????00-000001001????00??1010???00000?0101????00-1?1?0-??010000?2000000000000000?0000----0?0??100????001000000??000?0-0?00?0000?000?01000-

Nqwebasaurus_thwazi ????????2??????1000??1011?0-00??00?1???----0??????????????????????0-10?1?20111011002010011??0????010?0-0???????0?00?10??????????????????????????010?00?101???0000-??????????????????????????????????????????????????????????????03?11-21211???????--00-0???????10-1??01200?0-?11111?10010?????????????????????????????????????????????0???????0???????????1011101?10000??100?100?010?10??0?00000010?000000110101010122101111001010110??????????????????????????????????1???0?0????00??????????????????1??0??1?????000001000001011111?0010?0010?0?0002001110?001?0011000000001000?0101010

Acrocanthosaurus_atokensis ???010100?0??101?0??10?0?10-?0?1?0110001001000?0?0?0112011??0?00001010??1110--?11?0??0??0?01101111?00??10110??01001?1120?0??1?0??0?????01?0?0????1??0101?0?10000????10-?????????0?????200-?0000-?10???0?10111???0??0?0??101??000021001?00?0?0??1?0???1000?1??1?111110??001?11??0?10??2?1??01????10?1?00??01?0?1?????????0?02??????10?1?0????1?010?????????0???1?101?10?0?100000??10???02??00?101??0?0??01111?0?10100220002??100?0???00???10??????????????10??????????101010??0?0[0 1]0??0????00?11?10?00???01012?1001?011?0??00100?101?????0?0?????0??0??100????001?000-00??0?1010??2?01000-

Afrovenator_abakensis ?????0???????1?101001??0??11010100110?00000?1???????0?201100??0?001010011100--00??????????00010-01??0????010000001?010????????0?0???????????????????????????????????????????????????????????????????????????????????????????????02000100000???????0100-0110110?10-1?0001?1?11???0100?[0 1]?1010?????1?00?????0???????????0???0??0?0?0?100???????11?00??????????????????????????0??0?01??????00?????????1???011?1?0?????12100?2???010000000100100??0????00?00010000100?000000-0?0?01???000100010011010000-?0?0?1100010001000100????0?0??????001????00?0000100?111??1?0?110????01?????2???0???

Albertosaurus_sarcophagus ??1????0000011??0????10?1???0??0??0100000101110?11??002011001100011111011201??000-120000010111100112010101?001010100103000001000000000001?00010?1??1000?00110000100?10-?00???0000?001??00-?0?00-00-1000010111?011100???010??00?10?00??0?0000000?0001010???011?0???????0000011?00????0?0?0???????1??0000?00?00410?00???10??0??00??0??0?00?0????0??0000??01000010???00?000011?001???????00??0?00??01000000010?0???????320?0??-?0000????????10101?100000000?20???1000000?010??0001???00010000000102000101?1101100110000100100000001?1110011001?1000100?2000110?001?0011000001111000?0?1000-

Allosaurus_fragilis ??0010[0 1]01001010?0000?0000?1000010?110?0100100001?1?011?1-000000000101???[0 1]100--?1110100000-01000-010?0???01?0100010101120?00010??000100101?00?00?10?00101001100100-1?10-?0-0-?000000010000-?0000-00-1000011111?000100101010200000021001000000000000010100000111010-10000001011000010011010001000010000001001003000000000000020000001001000???1?0100???100??0001101010000001000000001000000000000001010000111100010101210002111000000000100101000100010000010000100000000101000?1010000100000011010000-00010110001100110010001000101110000?00010000000110001110?1?00110000001010?02001000-

Alvarezsaurus_calvoi ???????????????????????????????????????????????????????????????????????????????????????????????????????????????????????????????????????????????????????????????????????????????????????????????????????????????????????????????????????????????????????????????0---?00?0?0?0-001210000?1?????????????????????3020????????12100?0?1002???????????1??????????000-01?00?000?????????????????????????????????????????????????????1?011?1101000-10020--0110021002001101?00??????????????????????????????????00?11?1??001????????????????????10????????1011??11111001??????00?00?01110??10000-

Alxasaurus_elesitaiensis ???????????????????????????????????????0????????????????????????????????????????????????????????????????????????????????????????????????????????????????????????????????????????????1?00110101??10-101?0????????????????????????????00110?102110001010?0?????????????????0?????????0?00?010010011??00001001004?1??0??????????01??00?2?01??????0000?????????0???????0????0100010011101101????00??0?100010000000?11101220?02???00100100???110100?110101?0???????0-??000?2???????1?????011110?1?1?220?0-???????111?00???0??0?10???????????????1?????????????????00?010-?00?00???0?0?001?0??

Anserimimus_planinychus ????????????????????????????????????????????????????????????????????????????????????????????????????????????????????????????????0????????????????????????????0??????????????????????????????????????????????????????????1?????????????-????????????????????????????????????????????????????????????0?????????????????????????????????????????????0????????111100???10000?12???????2??????????????10000?1010011010?00221??111110012210????10100?100?11?0???????1010000?010????00???00?1000100010??000-???????????????????????????????????????????????????????001?1---?00?010110???0??1???

Apsaravis_ukhaana ???????0?????????????????????????????????????????????????????????????????????????????????????????0?????????????????1???????????????????????????????????????????????????????????????0??001010011100-0-0?????????????????????????????????????2----??????????????110-1?001?12110??1?11?00??0????????????????0???501210?0?210?10-0200-??2?0110????????11????103000-11100110101100?0001101110001010000111110001???1?11?1??????????????????11000-01010--0???1?1?1?000-10????312--1---1-00000-00000-1-22000??11?11112-2001?????1100???????????11????????11?????110?111?00-?000110?11010?001000-

Archaeopteryx_lithographica 1100001010010001010?1?00?00-000100010001???0000010001100111100021?0-0--1100111110-11010000??0?0-000000-010?00?000???0-210??12?010?0?0?????????0?0?01110001???0???????1001011?00??10010000-?0000-00-0-0?00-0?1?0?00-0??001010001002001?212010000?00--10-0??1???0?0-??????10??????0???????0???????????00???0??04????0???20?01??01??1??2?00?0????1?1110??101?2011-0110001111110020??1?00010????000?011?10?00?0?0?1???1?22??0??2??0000000???00-010???1001?1??0????0-01100?2?1????11???0011210000010?2101001??????11?0110?0??0?00??0??????0010?????00?0002000110?11110011?00?00?0?010?0??010-

Avimimus_portentosus ??????????1?1?????????????????????????????????????????????11??1?????????????????????0?000?????0-?01??????0?00?000???11????????????1?1?0?1?10?00?01??120?0?0??0?00-?110-????????0??00????11?????????11??10-0?0?00???????111-??-??????????2??2????-?--??????110?111010012000110?1121101001010001111000000000110601010?0?2?0000???00?????????????????????????2001001100001001100100101000100100000001?111????0???????0??2???????????????0100??1???1000011101010001111?00?0100100011-00011110230010?0000-011011210110100000101000001001100011?11100010112000110111100?11000102-110102010100-

Beipiaosaurus_inexpectus ?????????????????????????????????????????????????????????????????????????????????????????????????????????????????????????????????????????????????????????????0??????????????????0???1?00???1?1??10-101??100?????????????11------0220001100132010001010-0???????????????????????1???0???????0????????????????0????????????????????????????0????????????00??1001001?10000?0100?????????10????????0????00100?????????0?22?????1?00?0000?01011110011??????1???????????????????????0???????????????0???????1??????00???0?0?0??0????0?????010????????10?0??0??????001???11??????????????????0-

Beishanlong_grandis ???????????????????????????????????????????????????????????????????????????????????????????????????????????????????????????????????????????????????????????????????????????????????????????????????????????????????????????????????????????????????????????????????????????????1?11????????????????????????????????????????????????????????????1??????????1111101?01000?0020??000010000000????000????????????????????????????000002?0????????????????????????????????????????????????????1????????????10?0??0002100110?10001010???1100010?0?1010100?1000110???1?001?????00??10????001011

Buitreraptor_gonzalezorum ?????????0000??1?10???0?1???0??????????1???????0???????????00????0??????????????????1?10?010??0-001?0????0???0?10?001?????????????????????????????????????0?????????????????????????1?0?0-?0????01010???????????????????????????0??01-0020???00?00??00-0??110?010-??201210110??12??010???????0??1????0???1?1??01??0???????1??01??10?2?01??????1??2????10??2?1????11??10111001??????????0??1??????1?110??????????????22???????????????0100??????????01?10?0????111?111?1????010????20112122??0112?0?1001???????1??1??????????????????????1????????11?20001????01?0011?10??1-01001?0???1??

Byronosaurus_jaffeei ??000000110000010001?100110-000001110001????00001000??0000???0????110--11101110?0-?0????????????????????????????????????????????000?11?0?1???0??011?100111????010-??11001010111?????0?000-00000-0110-0?010??????????????101?001002201-002000120010--10-0??10-?????????2??0??????210?1???????????1??1?????0???????????????????0???2????????????????????????????????????????????????????????????????????????????????????????????????????????????????????????????????????????????1???????????????????????????????1???????????0?0?01??11???????????0?????????????????????????????????????1??

Carnotaurus_sastrei ??10000000001000---0??00?010000100010001???1?1001110??200000000?000-10101000--?10-11000000001?1101100101000010000??00-???0????1000??2??0?????0??????00?1001??0?0????????????????1?101100??00?01000-100?01000??0?11101000101000??01000000?0?0000000???0-000010101101?10?00??11102?10100?00100?10?10000111?110040?11?0??????00????0?????0000???0??????00--0?0000-?01001010?221?0110?200000-000111000----???????????????????????????????11000-0000??????00?00??00??0?00000100????1000000100?000?0-00000-??000?10000?0111011??011?00?110?0??????????????????????????????????????????????????

Caudipteryx_zhoui 100000??0?111?0?0000????????0?0?0?11??????????00???01?????000?01?0??????1?0101?10-?10?000010??0-00??0???00????000??0????????????1????????????????????????????0??????????????????0?201?001101111100-110?1??0???0?0?????0?10???1101?--??-12??2????01--?0-???????0?0-????1??00????????0?[0 1]0???00????????????????04?1??0???2100??11????0??00?0???1?011?00?110??00????1?001?0?0????1??10?0??0000?0??0001?110?0000?0111110?22??021-100?000000100101001???0?1?10?0?000?????00????????01???2011110??00102?0?0-?100?111111?0?????10??00?????????011???10????01200011?1001??0????01?1?0???1?0??000-

Ceratosaurus_nasicornis ??10100010001100---00?00?0100000001000010001111010101101-1000000001110?11100--0010010?000000010-011101010010100010?00-0?00?0??1000?02??01???000??0???1?1???10?00???0????????????1?????000-???0???0-10?001000?0??????100?100??000?11000?0000??0?0?0??00-0000111?11011000000011??0?00101?00100????10000011011004001?0?1?000?01000?0?01000010??10000?????????00010?01001000???????????????10001100101????0100?100010101110??1??????2????1100??0000????1?0000001001000000000-000101000100100000010-00000-0?11000000000011011000111011110?0?0000?1000???001000011?????????????00?0???????0?0?

Chirostenotes_pergracilis ??1????????1?00??????1101??????????????-??????????????????????????????????????????????????????????????????????????????????????????1?0100??01?1??0???120?011??0?00-1?110??????1????10-0?01111111100-100-10-0?0??00??0????????????1?--????-??2????-??????????????110??211??0?????12???1???????010?1??1?????????5210????0??1?0?????????????0?????????????????20?100???0?10?????????????????????????????????????0???????2200???????10000??1000-100010000??1??010000-000000?12??00?????201111023001121000-01????0?0??????0?0?11??0?0??????00??????????00?????1???0?1??0???00?01?010?0?0??00??

Citipati_osmolskae ??1000000010100101?0?110100-00020111000----0000100101?0010100001000-1101100101?10-11110000100?0-001200-000?000000??010?011011111101?0??01?01010?010112000000-0?001??11111110?0000021-0201101111100-110-10-01010001100-0111------1----------2------------??10-?110-?????210010??1????1?0?1???????1??1000??0?1?52???0???10?????1???0??2?01?1????0??000111111200?????00?110010?1?????1?00?0???000??0?1?10?00???0?????0?22??0????0010000?????0-100????001?1???????0-01000?110??0110???20?11102300102?000-01??????2110010?0?10?????0????????????????0000??000????001??0???00?00?0?0?0?0??000-

Coelophysis_bauri ??000011??0??000---????0?00-0101001?1?10???0?00001001????0000000000-0--11100--00100??0??0-000?0-01?00??0??0?????0???????0?????0000?0???01?????0????1???0?0?1000?????????????????0?001?000-0?0011???10?100-?000??????????1010?010?020000000?????000??00-1??0100?11000?02??0010?0?10000[0 1]0002001000????0??00?0?03?00???100000020?000100?01?0??????00?????????0????0011?00?0?1?0?1000010??0100????000??1?0000001?00?0?0?100??111100?0???001000-0000????100000001101???000??0-00???0001101100????10-1?0?0-??1100?0??0?0000000?00101?0?110???0??0?001????001000000??0???10???0?0?0????1001000-

Compsognathus_longipes ??00?011000????1010?1?00??1101000???0000???000000000??00001000000?0-0--111?0????1?1101??1?000?0-001000-0?0??????0???????????????0???????1???????????????010??0????????????????????0011000-?0?00-00-0-000100-0?000??1210?101000100210110010000000000100-0??0??1010-1?0?0?00010??0?1?0?10?0200????????100010?103????0??0000?0?000?01000010000-1?1000???1????00010?111?00000?0??1???1?0??01???000000????0?0101??0?10?1?22??12??10000011001??0-1000???0???0??0?0???????00?01111???00100001000100010100?0-?1??????000??1????1???00?0??????00?0??????0?0001000?10?001?0011000?00?0?0?02001000-

Conchoraptor_gracilis ???0????0011100?0?00??1?????0?020??????-??????01????1????110??010?0-???????101?10-?10?00001???0-?01??????0?000000??0?????????1??1????????????0?????0?2???1?????00-??110????????0??21-0?01101??1100-110-1??0?0?000??0???111-??-??1?--????-??2????-????????????????????????0??????????1????????????????01??0????11??0???????1??????????????????????0?????1?12?010???0??0100?0?1???????00?0???0????????????????0?????0????0?????00100100????0-100?11?001?1???????0-10000?110??00?1???20???1????010??000-0???????11?001??0?10?????0???????????????????0??????????01??0???00?00?0???0?0??0???

Confuciusornis_sanctus 11-000-01?000000----0?01?0--00110011000????00000?0?01?11-111001-1?0-0???1?0????10-??00?00?10??0-000?0????0?00?000??1?????????0??0???????????????????1?????0?????????????????????0?10??000-?0011000-100?0100?1?000??0???011---?--1----------2----??------??????0????????????????????0????0??0????0??100???1??05????0???20?01??1-?0?????010110??????11111010?0?????1?0?1111110020??1101110001?1000011?11?00?0?0?????1?22??0?????000000?1?000-010????0?1?1???????0-??100?30---??1????0010-1?????1?211011?11?????2-20?1?????0??0??1??????00???????01??1??2110???111100??001?10?01??0?0??000-

Cryolophosaurus_ellioti ???????????????????????????????1??1?1??000??0??0????0?2?????0?00001011??1100--001000??????00000-01?00??10?10??00????10?0?0???????0?????????????????10101?????000??????????0-????0???????????????????????0-0110??0???????????????0[1 2]??0?0000?????????????????????10-1[0 1]????00?????0???0??????00????10010??1?10?0???????00???0????????00?0?0????0?00??????????????????????????????0????????10000???????????????????????????????????????????00?????????????0?0??1??10?????00????????00????????00????10??????01001?010??011?0??0000??????????0??????????001100000?????????????????????????????

Daspletosaurus_torosus ??100020100111110100??00?10-00000101?000???11101??????2??0001100011111011101100011?01?010?000?0-011201010110?1?101001030???01?0000???????????0??????????????????100?10-?0-10?0000???1?000-0000??00-100001011????????211?10100001020000000000000000010100??????????????0???0????0????????????????????????????0???????????????????????????????1?111????????????1???????????????01????????????????????????????????????????????-????????0????????????????????20??????????????????????????????????????????????01?0002???1?0?10?????01?111???2??????????????????????1????????????1????????0?0-

Deinonychus_antirrhopus ??000000200001?111101000?00-000000010001000000010000110??1000001010-0--1???100?110??1???0?10??0-00??0???1001000?0??11031?110100?0??????????1????????00??0????0?0??????????10??????0011?00-?000??00-0-000101?1001???110101011000002100?001000000?000100-0001111?10-00101200011?0101100201000000001001011101100?????0?????00120010010?1001?1????1?12??????1?21110??110?101010?1?0???100010001000?00?11100001110101111122000212?0010000001?00-1??1100001?1??0?0000-111001210??0101???001111222001021010-0101011111111100001000001011111?00101??10?000002000110100110011?10?100011101001010-

Dilong_paradoxus 0-100020000110?101001100?10-00000011100????010210100012011001100000-11011100--00100011001000000-001200-0011?01010??011??0?????00000100101?00000??????1?1??1??0001??0????????????0?0-11000-00000-00-0-0000-11111111101010101000010200000000000100000100-0???????10-1?100000010??0010?0?0101?????????????1????0?????????????0?0??????2?000?0??1?????????????0?????1?1000?011???10001???????????????????????1?1?1?1110022???21????00000??1?0??1??010000??0??20000100?0?0??11??????????????0??????0??0????11?11?0001?00??0?10??0?001?111??010???10???0002000111??01???????0?00?0??0?2???000-

Dilophosaurus_wetherilli ??001020?00??0?0----1?????0-010?0010011[0 1]0[0 1]1??0?0?1001?2010001000010?0--1111???0010?0????0?00010-01?0????111????000?011????????0000?0???01???000??1?1?0?0?0?10000??101?????0???????1?1?1?0-???0???0-10?00101?01??????????1010?000?1000000000??1?10???00-0110110?1101?0011?0011?0??0?011000200?10?10000??1100?03000???0?0000020?000?01000?0???10?0??????????0??100010?00?0?100?10000100?0100?0??010?01?00?0001?00100011000?1?11000?????11000-???0????1?00?0001101????100?0-00???100?00?100????10-????0-??11000001000000000?0000??00110?000?0??100????001000000??0?0?10??0??0000?0?2001000-

Dromaeosaurus_albertensis ????????2?0??1??1?1??100????0???0?010??10100??0????011???100??0101??????1???????0-121?1101???????0??0?????????000??01031???0?0??000100001?00000?0?0?0011?01010000-1110-10-100000000011000-00000-00-0-000100111010111101010?000110?000?00000000000?0100-0???????????0????????????????????????????????????0?????????????????????????????????????????????????????????????????????????????????????????????????????????????????????????????????????????????1?????????????????????????????????????????????????????????????????????????????????????????????????????????????????????????????010-

EK_troodontid ???????????????????????????????????????????????????????????????????????????????????????????????????????????????1????11??????????0???????0????????????????10????1????1??0?????01?????????????????????????100???0????1???????????????????????0???????1???????0-???????????????????????????????????????????????????????????????????????????????????????????????????????????????????????????????????????1000010?01????1?22?0?2???0000?????????????????????????????????????????????0?????????????????????????????????????????????????????????????????????????????????0011?00?1????0???1???10-

Eoraptor_lunensis ??0000000000?000----0?00?01001000000100????0000000001?01-0000000000-0--11100--001001??0??-000?0-00??0??00000000?0???10???0???0000???????????????????????????????????????????????0?101?000-00?0??00-0-0?010?0??0?0???????101000000010000000000???0011?0-0?????0?0?????????0?10?????0?00??0?00????????0??0????01???????0????0???0?00???0??0??????00?????????0?????00??00?0???0?10?0??????????0??0????????10?01???0??0110???0100???0????00000-0??0???????0?00?100?????????0-?????00000?010?????00-1???0-????0????2??0?0???0??????????0--???????00????????????????????0-?????0??????????0???

Epidexipteryx_hui ?-?????????????????????????????????????-??????00???0???????0?001?00-???????????????0???0?0?0??0-001??10????????00???0??????????????????????????????????????????????????????????10?2?0?00???1?10-00-10001??001?00???????010?011100?01??2120?101?0?1??00-0??????0????????????????12??000???100????????00?0????04??0?0?0?2100???020?1??2??100????????10??????2?1?????0?100?101?02?????00010?0?0??001????????0??????????22??0??????00001?010?0-200011??????????0???????0??00??????????0?010102?????110????1????????????????????0????????????0?????????1??0001???111000110????0?0???0????????

Erlikosaurus_andrewsi ??100000100110000?-0?101110-00001101000????0?00????11????1000001010-0--?1100--?110?100000010000-00100??0000010000?0??0???1012100001---010????00?0?1000000000-??00-????????10??000?1010?011?1011010-101000-011?0000-0100011-?----0220001?0013111?001010-???????????????????????????????????????????????????????????????????1??????????????????????????????????????????????10?????????????????????????????????????????????????????????????????????????????????????????????????????????????????????????????????????????????????????????????????????????????????0????????0??0??????????0????

Eustreptospondylus_oxiensis ??0??020100?1100----00000?1100??0001001000?0???????1??????????000?1-11011000--0011011?00?000010-010?0????010?00000?00-????????0??0?????01?00010?01010001?0111?100-??10-00-10000??0??1?110-?0????0101001????????????0?????010000002000?0000000101000100-0110111010-11100101011?00?10001000200000011000001000?03000?0?0?[0 1]00002???00?00????????????????????????????1?00100?0200010001?000????????????????????????????????????????????????10???0??0???01000?00000011000000010000001010000100010?0?01?000-010100100000001000100010000011100000001100000000100011??00??????00000100000?001000-

Falcarius_utahensis ??????????????????????0???????????010??001?0???????????????????????????????????????10?0000?0?00-00??0??????????1?10010???????????????1?0??00?00?1?01000?000??0?011?110-10011010??0??1?00100000??00-1010?????????????????????????0?20001[0 1]00101110001110-0???????1101?212100010?1121111201020011101001010?0010041101000?1000120010011000010???101111???1010?10010?1?101001?1001?0010001111001100?00011001001010001111022000211100100000010111100111010101?00000011010??1110010101010001121023001022000-010?01101011000000100000101111100010?11??010000100011010001001100??001000???001000-

Gallimimus_bullatus ??0???00210000000101?101110-0?000???000-???00000000?11001?110?000?0-0--1???1110112000100?00?0?0-?000?0-001?010112??00-??01?010?10?0?110?1?00001?010?0001001??00011??110?0-100010??10-0??10?1010-00-0-0?01?1?1?1000-0???011------1---??-?-?-2---???---?????10??010-??202210010??12???0?0??????00?1??0000??0?00401??0???100?0??00??0??0?01?0????0??0????????011100??11?000002?0?0???2?0000??0000??1?0??001010?1?????0?2???0??2?00011010?1?010100?100011?0??0????1010000?010??01001-?000100010001010000-110?????00?1001?0????00?00??????00???00?0?0100??0001???00101---100?01?1?010?0001010

Garudimimus_brevipes ??0000002?000001001?0101110-00000111000----0?0000000??01-0110000010-0--111011101120?01001-000?0-00?000-00?00?00?001?113?01?01001000?11?0???0001?01?000010?01100?11?????????0???00???-0001001?10-00-000?00?101?1000-0?1??11------1----------2------------000?-1?????????????0-???2??00001010001001???0000001?04010100?0??0000???00?0??0??0????????????????????????????????????????????????????????????????????????????????????????????010011100010011100?1001001001000101001000101020??????????????????10101100011001000110010001011100000?0100?010002000110?00100011100000?1101020000011

Giganotosaurus_carolinii ???0?00????????0????10????10???1?001000100??01?1?0????2???????????1010??1110--??1?0???????01101111?01??1???????1?0??10[1 2]??????????1???????????0???0???101????????????1?????0-????1?????201-?0?0???10???0???1?11????????????1???000200?0?10?0?0??0?0???110001??1?111110??[0 1]?1?11??0?100?2?0??0?????10010??1?01?????0???0?000?01??????000???0???1000??????????0???????1?10?0?????????????????????????????????????????????????????????????0???10000?110?1??0?0?000?11?????101010??010100?0???????11?1???????01012?100??01??0?????0??10111???0?0??1?00????1???????????????????????????????????

Gigantoraptor_erlianensis ??????????????????????????????????????????????????????????????????????????????????????????????????????????????????????????????????????????????????????????????????????????????????20-0001111011100-110-1??000?00?0-????????????????-???-??-2------????????????????????????????????????????????????????????????????????101012?0000?0??0??????????1???????????????1?0?100??110?110002???00-00000000?????000101000??????????????????????????????????????????????????????????????????????????????????????????111110????10001??????????????????????????????????????1?????????????????????0?0-

Gorgosaurus_libratus ??101020100011?101001100110-000000010000???1110?11101?001100110001111?11110????0??121?0000001?0-00?20101011??10?0?????3?0000100000000??01?00000??????????????000????????????????0?00??000-00000?00-100001011110?0??0????10100001020000000000000000000?????????0???????0?0??????00??0??0??0000?0?????00?1????0??????????????????????????000???????????10?????????1???0???????????????????????????????????????????????32????????????????100101011100????0?12100?100?000?0101?0001???00?10010?0010????????????????????????????00???????????0?0?10???????????????01000110??????1????????0?0-

Guanlong_wucaii ??101000000111110100110011110000?0111000010010110001012011000100000-11011100--0?100010000-00000-001200-00110?1012??0??300?1???00000100001?00000?11010001000??000100010-10010?0000?001?000-00000-010100?00-1111??1??0????101000010200[0 1]1001000?000000100-1??????010-10101000010?1001001001010000001001001100100300010?00100000000?0??2000000?????11?????????00010?1?10000?01001100001001110001000001010000010101011100210002121000????0010010101010000000?02100110010000110110001000000100000000-100010011?01100011001100100000101111100?2000?1?00100011001111001?00110000001010002101000-

Haplocheirus_sollers ??00??0011011111010111001110000100111000?0?0000100001?01-1100000000-0--1000111?112010000?-10000-00??00??010011000??010?00110100000021?001?00010?010?0111??00-00110??????1?110??00?1011110-0000100100-000111010011??02100101000100120110010001101000100-0??????00-?--101?00?10?101110100101??00001??1?????10103000?0?0?[0 1]?00??00100?00?00000??1?01??????????11010011100001010011011011100?00?1100001?1000000110111110022011212101010001010???????11-?01002?000001001??00011010100??01001100000110110?0-01??0110101000000?1110001011110?001011010??000?20???1?10010?????00?00?010002001000-

Harpymimus_okladnikovi ??00000021000?010?0??1011?0-000?0?11100----000000000????????0???????0--1110111?112010?00?-000?0-00?000-00100100?????????????????0???????????????????????????????????????????????0?0010001001011000-000001010??0000000-0011------1------?--11?0??0?--?---?????1010-???0??0000-??02?0?0001?2??0?00????00110?110401?10???1000020000?0000001????0?0110?????????11???1?11100?012?0?00012000010000000011?10000010101011110221002111000111100100101001????11?0??0??00100?00000????010????00???????????????????0101?00???0?????????????????????10????????0001000110?00101---100000?0?000?0001011

Huaxiagnathus_orientalis ??0???000001?0?10100??00??11000???11??0????0000?1000?????110?0000?0-0--?1?0????1???1??????????0-?0??0????0??????????????????????0???????????????????????????????????????????????0?001?000-00?00-00-0-0??????????????????10??0??00?0?1-00100000000?0100-0??????0????????????11??10??0??0?0100????????10?01??10???????????0????00?0?120?1000??1?0000???100??00010?111000000100??????????00????00?00?0100?001??0??10?1?22??12111?000000?01?00-10001010???0??0?0??02??000?111?????????000100000001010000-?1??????0?????????????0????????????0???0????00?2000110?001?0011?0??00?0???020??000-

Incisivosaurus_gauthieri ??1000000101110100000100110-000111110001???0000100001100010000?1000-1001200001000-1010000010000-00100100000000010010102011012101101-00001?0110??11?012001000-?0010?11??1100-??0?0?2011?01111111000-0-0000-000?0?0110??0?101200?00?00??1?2001101?10--10-???????????????????????????????????????????????????????????????????????????????????????????????????????????????????????????????????????????????????????????????????????????????????????????0?????????????????0?????????????????????????????????????????????????????????????????????????????????????????????????????????1?????????

Limusaurus_inextricabilis ??1000101001110101?00?000?11?0?1?01?000?---00000?000??01-?000000000-10012100--00120??0????00000-00?1????0000?0?020?00-?????01?000???????????????????????????????????????????????0?20-?00??01?1110-?0-1000-?0?10?0??0????11------1-----------------------000??0?1101?1?2?0000-??01?0?0???????????????00?1????0?????????10000?000???020000000-0??1101000-1??000100011010000?10011011?00?00000?000000----?0000-00010101[1 2 3]10-00011--02???000000-000110001000?100100100100000100???0?0000?00-??000?0?00?00?1???00?010?0?0?????00011????????????0??10??????1???????001?0???00??0??00???2?00000-

Linheraptor_exquisitus ???000002000?1?1101?1?00?10-00000001000????000?0100???0001000101010-???1110100?10-1?10000?100?0-00?200-00001010?0?0?10????????0000???????????????1?10101????????????????????????0??0??000-000???00-0-0?01011????0??111???01?????02101?0?1000000??00100-???1111010-1??00?0??11?11??1?????????????????00?????10?????????10?0???000??001000?1101???1?111???????????1???1????10?11???01???1?????10???????????????????????????2????????????1?0?????????????????????????????2????010????01??????????????????1???????????????????00?101??111??10???10???0??2000110??011001??10?10?0?10???01010-

Mahakala_omnogovae ???????????????????????????????????????????????????????????????????????????????????10??000???????????????????????????????????????0?????????????????1001??1???????????10?0-10?????????????????????????????????????????????????????????????????????????????????????????????????????????????????????????????????401??0???????1??01??10?2?????????1???????????????????????????????????????1???10??????1110?0??????????????????????????????1000-0???0-??11?1???????0-11100??????????????????????????????????????1?11?111?0?0???0?0????????00??????0???00??0001????01?00??010?10-?1??0?0??010-

Majungasaurus_crenatissimus ??10100010001000--??100000100001000100011100?1011010112010000000010-101?1010--010-110000?0011?11011111011000100010?00-0000001?1000012??01?00000?1??1?????0???0?0?????0-?????????0?10110???0?00?????10?0?1000100001001000101000000110000000000000000100-0000111010-1?10000?011?020001010001001101?00000010110030??1?001100002000000110000000?100100?????????000-00??0?0?002210010012000??????????0???????????????????11???????????????010010000010001000?000100100000010????????????0???0??????-??0???????????000000??00100011101101010000111100010000100010??0??0???0000000?0??0?0000011

Mapusaurus_roseae ?????0?????????0?-??10????10?0???001000100?1?1?1?0??1?2??1???????01010????1????1100???????011?1111?????????????1?01?10????????????????????????????????????????????????????????????????201-?0?????10???0???1?1???0??0101?????????0?00?0?10?0?0??0?0???110??1????11111???001?11??0?100?2????0?????10010001?0????1?????????0?01??????1001??????1???0????10?????????1?1??0???100?0???11?1????????????????????????0??0??????????????0?????0???10000?110????????00??110????10??????????????????00?11?10?00???0?012?1001?011?0????10??10?110000?00?0?00???1?100011??0??????????0?1?10??????????

Masiakasaurus_knopfleri ???????????????0--??100???0-00???0100??10000????????????????????????????????????0-??????????????????????????????????????????????????????????????????????????????????????????????????1?110-01?11100-1000?????????????0-??????????0?00?0?00?000?00010100-0???????1101-10??00?0-1?2110?0???01??00001??00010?01?0???01?0?110?0020????0??0?0???????????????????00010???????????100?1001?????????????????????????????????????????????????????????????????????????????????????110?01000000????????????????????0100100010000100100011101011000000?????????1??1001111001?????000?0???00????001?0-

Megalosaurus_bucklandii ?????000100?1?000---????0?1000???-1100001101????????????????????????????????????????????????????????????????????????????????????????????????????????????????????????????????????????1?000-?00???00-??010100????0????????????????0?0001000000?0000?010100????????0-?????????????????0?[0 1]?1??00?????100?????0???300010100??0??2????0??0?1????????????????????0??0-?0?0??????2011?0201??????00?0?????????????????????????????????????????010?10000110000000?01000?100000100??00000????000100010?1??0??????00000100000001000100?1010101100??0????????????????????????????????001?00?0????????

Mei_long ??00000011011?00?-??0?00?12100000?11000?????00001000??01--11?0?11?0-0--12?01110?0-1100?000??0???001100-0???????10???11??????????0???????????????????110??????0???????????????0??0??????00-0000??0110-0??????100????1????10?0?0?001201-0020???0???0--?0-0??110000-?--101210110??101001?0?11000?00????00?00111040??1??0?1000?0001??2--0-0?0?1?0???12???1111?20110?110011010000010?11100110???????0?????????????1???????2??????????0000001????????0--00??1?0011000-0110000????01001-0101??10???0??20??10?10?01??1110110?0?11?000?????????011?0?100??0112000110?00100011???1?1??10112101010-

Microraptor_gui 100????01000???1??????00??0-0??????????????00000???0?????1?0?0??????????1?0?????????????0??0??0-00??????????????????????????????0???????????????????????????????????????????????????10?0???000??00-0-0????????01???1????10???01?02?0??0?10000???00--10-?????????????????0?111??1????00??01?????????100?0?1?004???1????10??1?001??1--1-01?1??????120011101??0??????1??111110?11???0???110??????000?110000000101?0??0?22??02??1001000000100101?0?111??1?1???????0-011?1?20--?010?1-0011121?2??011011010?1??????11?111????10???0?0??????00??????0?0?0???0001???10110011?10?11?0??01?101010-

Microvenator_celer ??????????????????????????????????????????????????????????????????????????????????????????????????????????????????????????????????????????????????????????????????????????????????[1 2]0????11?1101100-????00-00????????????????????0???????2??2????-?--??????1101?110???????0010?01?1?102??11000?001001000??0?10?0???????????1?11??00??2?????????????????????2001????00?0?0?010110??02?0010000?000?0???????0???0????????????2???0010?00001000-1001101001???1010000-00?0010100?0101???20????????0?????????1??11?10121010000100??000???11?001??001000000120001101??????????????????????????0-

Monolophosaurus_jiangi ??001000100101?10000?00?01110001000?0000??001111000100201100000001111??11100--00100000000-000110011000-0011000001??010????????0000?????01???00?????1010100?100000-??????????????0?0011110-?0000-00-100?01001????0100211?1010000002000?00000000000?010100??1101?10-1?100?01011?0001000101010000011?0000?1001003000?0?????0?02????0?????0??????????????????????????????????????????????????????????????????????????????????????????????0100100000100100?0?0100001000000000-??0101000100100?00110-100?0-???????????????????????????????????????????????????????????????????????????????????

Mononykus_olecranus ????????????????????????????????????????????????????????????????????????????????????????????????????????????????????????????????????????????????????1100?????????????1001011??0??1??????????????????????????????????????????????????1-212?1??0????--10-0???????0-?--1010?11????121101011020010000-?101???1010??2??1?????0121?0?1??????????????????110???001000-01?101000023000010111-0021000111011?10101111101101100221112-?111011?120?0?0-???????????????1?00111??00?30---???1???00?0-0000??-?????0-?10011112-20010010111000011011110011?11???1011??1101101001?0011000102-11000?010100-

Neovenator_salerii ??00?01010000101000010000?1100010011000101000101110?????????????????????????????????????????????????0???????????????????????1???????????????????????????????????????????????????????1?100-?000??00-??00?????????????????102100000?100100000000010?010100001111011111100001011?0001000200000100011011011100100?010???????000100000100010?10??100100????????0001101?101010?????????????????????????????????????????????????????????????0110100??010001000??000001000??11010100101010000100000011000000-00010120001100110010101010111110010001101001????100?????01??????000?01?10002001000-

Nothronychus_graffami ????????????????????????????????????????????????????????????????????????????????????????????????????????????????????????????????????????????????????????????????????????????????????????????????????????????????????????????????????????????????????????????????????212?????????2????20?0?001111???????10???04?10?0?0?2010??10?0??0?000100?????00????101??00?1?00???10000110?1?111101??100?100000?????????0?????????22????????000000001011120011??1???1?0??100???1100?1101000111--2?0121?0?11102?10???0??112111100??00?000?0010110???111??1101?0??0?2000?1??0001010-0000001?0110?0?0??0-

Ornitholestes_hermanni ??00?0000?01?00101011100??10000?01101000000000?0?001110001100000010-10011100--00101?01000?10000-000201000?001000000011[2 3]0????100000???0?0????0?0?01111101?100-????-????????????????0010?10-?0000-00-100?00-011100011010001012011102101-001000000?010100-????????10-1?101200011??1111?11?101??01001??1001010110?0??10?0?10?0020000?10?0??????????1?1??????????????????????010?1100001001?0??0?0?0?0?????0???????????????????????????????1?0101??0100000?0?0010011000000?1????010?01000010?000001?10000-0????????????10?0?10?????????????????00??0?????????????001??????0?100?0?0?0?0010?0-

Ornithomimus_edmontonicus ??0?????210000???11???01????0?020?????????????00???011?0111100000?0-0--????1111112?00100?00???0-?00?????010010101??011??????????0?0?11??1?00????0???000?0?0??0?0???1110?0-10?010??10-0??10?1010-00-00??01?1?1?111??0???011------1?-???-?-??2??????????????10-?010-?1??22100???102???00??0???01??1??0?00??0?00401?00???100?0??0???0??0?01?00-??0??0????????11111???01?000002?0?????2????0??0000??1?0?0001??0?1?????0?22??0??2?00012210???010100?10001100???????1010000?010??010????00010??10001010000-110?????00?1001?0?10?0???0??????00???0??0?0100??0001???001?????100?01?1?0?0?0?01000

Oviraptor_philoceratops ???100??0?11?????1????10????0?0?0??10??-???0??01????1????01000???00-????1?0101?????1???00??0??0-001?0????0??0??00???10???1?1?1??1?1?0?1???????????????????0????00-??????????????0?21-??011?1111100-110-10-0?0?00???0????11-?????1?--??-?-??2????-???????????????????????????????????????1???????????0?????????????0???????????????????????10?????????1111??????????0??1??1??0???????00?0???0????0???1???????0?????0?????0????00?0000?????0-1???????0??1??????????????????????????????????????????????????????????????0?10?????0??????????????????????????????01?????????????????????????

Parvicursor_remotus ?????????????????????????????????????????????????????????????????????????????????????????????????????????????????????????????????????????????????????????????????????????????????????????????????????????????????????????????????????????????????????????????????????????????????????[0 1]1??2?0????0???????010???????1?????0121???10??????????????????????????????????????????????????????????????????????????????????????????????????????0???????0--?????2?01?0?0-?1????30---1---1--0000-0000----1[1 2]00???1?011112-200100101110000101111100011000?01?101211011010010????000102-11001?001100-

Patagonykus_puertai ?????????????????????????????????????????????????????????????????????????????????????????????????????????????????????????????????????????????????????????????0????????????????????1????????????????????????????????????????????????????????????????????????????????????????????????0?00?01?0????10000????1???302011000??012100?002??????????????????????0?1000-0?????001???0??011?111002100011???????00?10?10??????1??1112?????011?11010???????1?0???????00100101????0211011---01001???????????????0-0??????1112000100011100001111111??10111??000010[1 2]001100110??????1????000??????010?0-

Pelecanimimus_polyodon ??00000021000101000111011?0-00010011100-???00000?00000001111?000??0-0--1120111011202?1?01-000?0-00???????????????0?010?00???1??10???????1?00?01???????????1???01????1?????10????0??00?001000?1??00-0-0001010??1?0??0????1030001103201-102110201010--10-0???101010-1?????00010?1???1000?1????????????????????0?????????????????????????00?110??????000???101?????1?11000?0?200?0001?00?0100?0000011??00?1010011?1110?22100112100011210????????????????????????????????????????????????????????????0??????????????????????????????????????????????????????????????????????????????????????

Piatnitzkysaurus_floresi ?????0?????????1??????????1000????110?00100?1??????????????????????????????????????0????????????????0????????????????????????????0?????01???0????10??1?1?0?10?0010?010-?????????00????0?10????????????0?????????????????????????0?1?01?00?0??0?0????0100111110?10-1010?1?0011????10001?1010?????11000??1?00??30?0???0???0?02????0?????????????????????????0??11?0?0?00?0?1?0?10?00??0??100????0??????????????????????????????????????0100??0????????????010000?????????1?00????0000?010?????11-1???0-??110010?0100010?0100??0??10111???0?0????0??????????????????????????01?0???????????

Proceratosaurus_bradleyi ??10??000000011101001100111000?001110000???0??1???010?001100010000?????01?0????01????????????00-?0??????0110?0000???112?0??0??0?000110101??011???????????????0??????????????????0?001?000-00001000-100000-1111100??0???01010000102101-0010000000010100-0????????????????????????????????????????????????????????????????????????????????????????????????????????????????????????????????????????????????????????????????????????????????????????????????????????????????????????????????????????????????????????????????????????????????????????????????????????????????????????????????

Rahonavis_ostromi ??????????0???????????0????????????????????????????????????????????????????????????????????????????????????????????????????????????????????????????????????????????????????????????????????????????????????????????????????????????????????????????????????????????????????????????012011100000?10010010010?041001?000??10120010010020??????1?1112??????????????1100110??????2????????10001010000????????????????????????????00??????01000-21011110011100010000-011100211??00111--0011210000011121011010001112-20110000100000101111110011100100100012000110100110011011110101110?001010-

Rinchenia_mongoliensis ???1?????011100?0?00??1?????????????0??-??????01????1????0100?010?0-10?????101?0????0?00??????0-?0??????00?000?00????????1???1??1??????????0??????????0???????????????????????????21-0?011?11111?0-??0-10-0?0?0?0??0???111-??-??1?--??-?-??2????-?????????????????????????????????????????????????????????????????0??????????????????????1?????????????11??????????0??1?0?0?0??????????0???0????????1????????????????????????00?000??????102???111?01?1??????????????????????????????????????????????????????????????0????????0??????????????????????????????0???????????????????0??????

Sapeornis_chaoyangensis ??0000000?010??1?1??0???????0??????1?00?????00001000??????11-002?00-???12?0????10-11???0??100?0-0000?0-0??????????????????????????????????????????????????????????????????????????10-?001001010-00-0-0-00-0?1?101??0??0010100?100?011-2120?2--------?0-0??????0??????????2??????211???0?????0???????????????05??01??0?210-0??1-0000??????0???????????111??2010-?11001101111012?001?011100?10000001111100010101111?1022??020010010000001000-11010-???1?1??010000-0?100?3110101101-01010-10001011010011010?11112-2?11??00100?0????????????1????????11??1001???11100010??0?10??10?0?0??000-

Saurornithoides_mongoliensis ??1000??1100000??10?01?0????0???0?01???01000???0?????????1??????????????110?????????????0?????0-00??0????????????????????1?01????00?11????????1????????????0-0??0-????????0-?11?0?000?0?100000??011??0?0???????????1?????0?000??02101-0010001000101010-0???????????????????????1???0?00?????0000?00??0?000??0?010?000???001????00????????????????????????????????????????????????????????????????????????????????????????????0?????????????????????????????????????????????01?1???0??111023001020000-0???1111111011??????????????????????????????????????????01??????????1???0???1???1??

Saurornitholestes_langstoni ???????????????????????????????????????1??????0??????????1??????????????????????????1?11?1?????????????????????????????1???????????????????????????????????????0???????????????0????1??00-?0?????????0????0???????????1????1???0???0??0?1000000?000100-???1111010-1?1??200011?11?10?12??00??????1??101110110?411010100????1??0???1??2?01?1????1??1????????20???????????????0?????1??00?0?00?1?000??1?0?00?010??1111022?002???00100000?10?10210110100??1?0010011100100??????????????0???1????0?0??0??????????????????????????01011111???101??1?01100110001100101?0011?10?10?001?0?001?10-

Segnosaurus_galboensis ?????????????????????????????????????????????????????????????????????????????????????????????????????????????????????????????????????????????????????????????1????????????????????0010?????1?1??1??101??0-0?1?000??0???0????????0???001?0?10111?00101???????????????????????????2??????????????????????????????1??0???????????????????????????????????????2???????1???1??11?0?0??110110?????????????????????????????22???????00???????1011110011102010000101000-01000?21010????01000011110?1?102?101??0??????????????0?0??1??0???????11???11?????0??10000????001010-?00000??01102001????

Shenzhousaurus_orientalis ??00000021000001000??1011?0-000?0011000?---0000010001?????1???????0-0--1120111?1????0?00??0?0?????00?0-0???0?01????0????0???1???0????????????????????????????0??????????????????0?001?001000010-00-0-000??1?1?0????0?????1------1---1-----11001001--00-0?????????????????0?????????0?00??200?0??????00?0001?0411??0??????000?0000?0??0???0??0?01???????????????????????????????????????????????????????????????0110?221???1210001011001000-100010101000???1000100?0001010010100?1?010100000000010000-???1????001100??00100??????????????????????????????????????????????????????????????

Shuvuuia_deserti ??000000110000010101?101102100000?11000-???00000000011000-11001-1?100--122011101120200000-10000-001000-0100000000??10-??01?120000002101001--010?011111?00100-0010-??11001011110001100?001000001000-0-0000-0010011110??001????0??03201-212110202?10--10-0??10-??10-10100211110?11210?101?0???0???0??100??01?105020?1?0010012100110100200101????1110110???001000-0110000000230000101?1-002????110?11?10101111-01101--0221112---1101121201000-10020--0111021012001111000?30---1--11-00000-0000----22000-010011112-200100101???0??1???101?001??????101112110110100100011000102-11000?011100-

Sinornithoides_youngi ????????1?000???????????1???0?00??????????????00??????????????01????????????????0-???????????????0??????10?????????0?????????????????????????????????????????0???????????????1????000??00-?000??0110-0???????????????????0?000??0?101???1??0100?10?010-????????10-???0??101?????????1???????????????????????0?????0???10??1??01??1??0??1?0????1?12??????1?20110???00?1??001???????2?0?10????????????10?0010?01??????22??02????0?0000??????????????001?1???????0-1???0?0??????01???00?1210??00?0210?0-?1???????1?011???????00?????????00??????0???0???????????0100011?0?10????011?101010-

Sinornithosaurus_millenii ?000??001?000??1111?1000??0-00001?11??01???0?00010????0000000?010?0-0--1??0100??10111?1000100?0-001201101?0?0??0??0010??????????0?????????????0?????????????????????????????????0?101?000-00000-00-0-0?01010??0?0??1????101010000?10010?1?00000?000100-0???????????????????????11???1???????????????????????04????0???????1??0??????2?????????????00111?1120110???10?101110????????????0????????0????0000???1?????1?22??02?2?00?0000?????0-1???11??01?0???????0-11??0?211?????1???011121????011211010?1??????????1????????????????????????????????0??1001???001?00???10??1?0???1?001?10-

Sinosauropteryx_prima 000000000001?001??0?0?????0-0000????0?0????0000???????????100????00-0--?1100????11?????????0??0-00??????00??00012?0?10??????????0????????????????????0???????0??????????????????0?001?000-00?0??0??0-0?0????????????????10100?1002101-0?1000000?000100-0??????010-1?1?0?10?10??11??0?20??100????????10?01???03????????000?0?000??1120?10000-1?1110????????00010?111010000200?00?00????01?0??00000?1100?0111101?1110?22??121110100010001000-1000???0???0??0?001020?000?111?????1???000100000011?100?0-?1??01?0001??0??0?1????0?01??10?00?0???0??0?00?20001101001?001100??00?0??0020??000-

Sinovenator_changii ??1???0001010??0010?1100111000?11???000??????000???011????????0?????????????????0-???0?000???????01????????00??12??011??????????0?0?00100????00?0??0100?110??0?10-??11001010?001??1?0??0???000??011100?0??0?1?0?0??????0101?00??0?2?1?0?10?010??1?01?0-?????????10???0?210?10??01???00??????00?01??100???0?1?401?00???10??1??01??2??0????????????1????????20110???10?101????????????????????????0???10?001??????????????????????0000???????????111????1???????0-11?00?211??010????0011210??0011210010?10?01?11110110?0?11?00??11?111?0011???1000?10??0001???001??????00?10???1?1?1???1??

Sinraptor_dongi ??001000000000?10000000?010-000110110000?0000001000000200000000100101??11210--?1100110000-010010011000-00010000010?0113000001000010000101?00000?10?00101000100100-??10-?0-0-?0?00?1011000-?0000-010100?0111010000??010101010000002100?00000000000?010100110111010-10100001011?00110011010101010011000001?0100300000?????0002?00?0??1?1001???1??1??10????000?????1010100?0??????0????????????????????????????0??11101210?????1000000100100101001???000?0??100001000000001100??11010000100000011010000-00010?1000110011001000101010111?000000?00?0000001000011000?00110000001010?0100?000-

Stokesosaurus_langhami ???????????????????????????????????????????????????????????????????????????????????????????????????????????????????????????????????????????????????????????????????????????????????????????????????????????????????????????????????????????????????????????????10-1?100?00011?00???0010001000??1?0???00?00??030001000???000????00???????0???1????????????????????????????????????????????????????????????????????????????????????????0100110??1100?0100??21001100?0000011?1000?00001010001000001?0?101????????0????11001010101010110001??0??????????????????????????????????????????????

Struthiomimus_altus ??0???0021000??1?10?110?110-0000011?000----?0000100?11???1110?00010-0--11?01111?12000?0010100?0-?000?0-0010010112?1011??01?0???1??0????01000????0??0000??0???0?010??110?0-100010?000-0001001011000-00??0101?1?1010-0???011--?-??1----------2------------??10-?010-?????2100???002???000?????0???1??0000??0?00401?00???100?0??00??0??1?01?0????0?10????????111110??11?0000020010001200001000000001?0?0001010011111100221001?2?000112100100101???10001100?10????101000000100100001-0000100010001010000-110?01?00011001?0010?0?000???1100010?0?10?0100?200011??00101---100?01011010?0001010

Suchomimus_tenerensis ???101201????0?0?-???1?0?111???0?01?001000?01?????????????????000??0???????0--??100????????000?????0??1?????????????10????????0???????????????????????????????????????????????????????110-????0-???????????1??????????????3???00022000?01?1?2????0???0-1?01??1?10-??0??????11??0?1?0?1?1??10????11000001?01?0???????????0???????0??0???0????0100??????????0??0-?0?0????001010102?1??0??21001??10?????????????????100???0?????0100????0?0?10000?????1??0?01?0????0?0???00?????0?[0 1][0 1]00?0????00?11?10??0???01011?1001?010?0??0??0??10111???1?1??0?00????110001??????????????????????????????

Syntarsus_rhodesiensis ???0?0211?0??000-??0???0000?0101001?1110??1010?001001?11-0000000000-0--122?0--00120100???-?0000-00?00??0110????00??010??00??1?0000?0???01???100??0?0?0?0?0?10000??0010-???0?????????1?100-??0011?100-?10101000??????????1010?010?010??00000??1??00??00-0110??0?1100?00???001000?0000010002??100010?00??0?00?03010???1?0000020?000?00001?1?0-1??00?????????0??1000?1000?0?1?0?10001100??1???0??0?0??1??000001?001000110?0?11110000???011000-0000????1000?0001101???0001?0-00???0001101100010110010000-0?110000??00000[0 1]010000101?10110?000?10?1010??1001000000?10?0?100?0??000????1001000-

Tanycolagreus_topwilsoni ??1??000?00?00????????????????????????????1?0000???0??????????00000-1??12201?00?????????0?00?110?1?????????????000?010??????????????????????????????????????????????????????????????????????????????????????1000????101??01??0?10??0??000?0????????????011???1??0-??1??????????????0?0?00100????1001100?001?0?0?????????00?0???????20?00????1?????????????00010?1?10000??101110?011?0111000000010??11000010100?110002210?20?100000000??????????????????????????????????111101?1????0??????????????????10101100011000100100000?010111?00100011000100?2000111100100010?0?00010?0002001000-

Tarbosaurus_bataar ??0???00000011010000?100110-000000??0000????110010100120110011000?111101???100001??01000?-0?1010?112?111011001010??01031000010000?000??01?10010?1?000001001??0000-??????0-?????0??0011?00-?0000-00-100001?11110111001010101000010200??0?0000000?00010???????????????????????????????????????????????????????0????????????????????????????????????????????????????0???????????0????????????????????????????0?????????32????????????????????????????????????????????????????????????????????????????????????????????????????????????????????????????????????????1?????1??????1??????000?0-

Tawa_hallae ??0001201000??00----01001?11000?00000?1????0?000?0011?200?001000010-0--12100--?1100??0????000?0-00?0?0-??11010000??0????????????0?020?001???000??????000??1??0??????1?????????????10??000-00000-00-100?00-?0????0????????00??00002000?0000?0000?000100-0???????1100?00??00?1?????0?0????????????1????1???0???????0???????0??00????0?0?00????????????????????????1?10100?0100110000????????????0??1????000??10??00?01110001110?000000000000-0???0--?0?00??00000100?000?0110????????10?????????????????????001??2000000??100????????????????????????0001000000000?010-?00?00???00?1???????

Torvosaurus_tanneri ??001010???????0-?????????1100????00000101??????????1?2??10000???0101??1100???00???????????0010-00?????????????0?1?0?0????????????????????????????????????????????????????????????????0?0-???????0-??????????????????????00??0??0?0001?00?0????1????010000???1?10-1000?1?1?11????10002?10100????11000??1?0??03??????????1?02??0?0??0?1??????11?0??????????0??0-0000??????2?0??0201?00?0200?1?01001?????01111?0?10?01?????0????10????00100110??0????00?0?000000?????000?0-00???0000000100?10?00-1?0?0-???0??1????0?010?0??0??0??00111???0?0????00???011000111?????????????01?0???????????

Troodon_formosus ??0???001?0?0?010?011100?10-0000?1??0??1??????00???0????????00????110--????100??0-?00?00?01??00-?01??0-0???0?0?1???01030??1?11??0?0011000???001?010?1000001???010-1110-10-10?111??1?1??010?100??0110-00????????0????????101?00?00?201?0?1000220?001010-????111?10-111??210110?01?10?100101??????1??1011??0?10501?10?0????011?0?0?20?0??1?????????2??????????????1?00110???0?1?0?10??01?0????????0????000010?0???????220??????001?????????0-???????????????????????????010??0001???0??11??23??1?21000-0?0001?11120110?0?1???0?00110101??1????1?0100012000110?001?????100111?1?011?1??010-

Tsaagan_mangas ??00000020000?011010?100110-000000010001???00000100?1?0001000101010-0--1100?????0-11100001100?0-001200-000010?000?00103????0?000000100101001000?11010101100???0000??10110-1000000?001?000-00000-00-0-0?010011101011111?010101?0002101?0?1000000?000100-??????????????00?0?????110??????????????????????????????????????????????????????????????????????????????????????1????????????????????????????????????????????????????????????????????????????????????????????????????????????????????????????????????????????????????????????????????????????????????????????????????????????????

Tyrannosaurus_rex ??10?0000000110101011100110-00000001000001011101101000201100110001111101110110?1111010000-011?1001120111011001010100103?00001000000100101?10?00?100000010010-0?010?010-?0-100000000011000-00000-00-1000010111?011100101010100001020000000000000000010100000111010-11?00000011?00010001000000000010000001001003100000?0100000100000020000000-100110???101??0001001010000001100010012?00010001001001?0?0000101000----03200021--00000?00010010101110000000?1200011000000101011000101000010000000102100101011012001210010001000100010111?0010?0??0?0100020001101001?0011100001-110002000000-

Unenlagia_comahuensis ???????????????????????????????????????????????????????????????????????????????????????????????????????????????????????????????????????????????????????????????????????????????????????????????????????????????????????????????????????????????????????????????????????????????????0?20?1100????10011011011??4000?01?0??????????????????????10??????????????????1?00110?010011021????????????????????????????????????????????????????01000-2100101001110001000101011101110101011-010?1110210011????110100010010?1110000100?????????????1????????????????????????????????????????????????

Velociraptor_mongoliensis ??00000020000?0111101100110-000000110001???000001000110000?00101010-0--1120100?10-11101100100?0-000?0???00?10?000?0010?1?1?0100?00??0010?001?00?1?01001?000??0?00-??11110-100000010011000-00000-00-0-000100?1?0?0111101010?110000?00110010000000000100-0??111?010-???012000????10??01???1?00??0?1001011?01?00501010?0010001??010?1????01?110??1?120011001020110??100?111010?1?0??01?0010??1100??0?1?10000???010???0?220002?2?0010000001000-110011100101?00?1000-0110002110?01011-0001111022001021010-0111??1?11?11?000010000010?1???100???0????0000?20001???101?0011110010001111?001010-

Zanabazar_junior ??10000011000001010101001110000001110001????00001000??1???1????????????????111?????00?00?0100?0-00120100?0000?0???????????????????0011000????01?0??110001010-0010-??10-???0-?1110???0?001000000-0110-000???????????1100?1010001102101-0010001000101010-0?????????????????????????????????????????????????????401010?00???0??00???2-?0?????????1?12?????????????????????????????????????????????????????????????????????????????????????????????????????????????????????????????????????1??????0?????????????????????????????????????????????????????20001?????1???????????-?1???????????

Zuolong_salleei ??00??00?00?010101001?0?0?0-0000001110000000??????00???????0??010?0-0--1?10111011????00?0?00000-0?1100-00?100000001011300??0????0???????????????????????????????????????????????????????????????????????????????????????10100001020?0000100???????010100???????10-0?101100011??0?10??00?01???????????????????40001?000??000000??0?00????????????????????????????1?1?1???010001???010???????00?000???????????????????????????????0???00100??????????00?0???0???100?0000011?0010112?00???????????????????1?011000100001001000001010111?0???011??000????????????010????100010101000????0???

Santanaraptor_placidus ????????????????????????????????????????????????????????????????????????????????????????????????????????????????????????????????????????????????????????????????????????????????????????????????????????????????????????????????????????????????????????????????????????????????????????????????????????????????????????????0????0100????????????2???????????????????????????????????????????????????????????????????????????????????????????????????????????????????????????????????100000?0?01100100?110110001100110010000000111110001?????????00120001101?0100???1????01??00??0??0???

Timimus_hermani ????????????????????????????????????????????????????????????????????????????????????????????????????????????????????????????????????????????????????????????????????????????????????????????????????????????????????????????????????????????????????????????????????????????????????????????????????????????????????????????????????????????????????????????????????????????????????????????????????????????????????????????????????????????????????????????????????????????????????????????????????????101200121001100100??????????????????????????????????????????????????????????????

Bicentenaria_argentina ????????????????????????????????????????????????????00????10?00001???????????????????????????????????????00??0?1?110113???????????00111?1??????????????????????????????????????????????????????????????0?01???00???????0?0???0110?????0??0????????????????????????????????????????????????????????????????????010???????001?0???0????????????????????????????????????00?????????????00?0???????????????????????????????????????????????????????????00?????????100???????????????????????????????????????101100010000100100?00????????????????????????0??????????????????????????????0???

Aratasaurus_museunacionali ?????????????????????????????????????????????????????????????????????????????????????????????????????????????????????????????????????????????????????????????????????????????????????????????????????????????????????????????????????????????????????????????????????????????????????????????????????????????????????????????????????????????????????????????????????????????????????????????????????????????????????????????????????????????????????????????????????????????????????????????????????????????????????0?10?0001010111?0??????????????????????00?000?1?00?1??01?0??000000-

;

cnames

{0 Contour_feathers absent present;

{1 Vaned_feathers_on_forelimb symmetric asymmetric;

{2 Shape_of_premaxillary_body_(portion_in_front_of_the_external_naris) wider_than_high,_or_approx._as_wide_as_high significantly_higher_than_wide;

{3 Premaxillae unfused fused;

{4 Premaxillary-nasal_suture_dorsal_view v-shaped w-shaped;

{5 Premaxillary-maxillary_suture scarf_or_butt_joint interlocking_joint;

{6 Premaxillary_body_in_front_of_external_nares rostrocaudally_shorter_than_body_below_nares_and_angle_between_anterior_margin_and_alveolar_margin_more_than_75_degrees rostrocaudally_longer_than_body_below_the_nares_and_angle_less_than_70_degrees,_naris_overlaps_premaxillary_tooth_row much_longer_than_body_below_naris,_naris_located_posterior_to_premaxillary_tooth_row;

{7 Ventral_process_at_the_posterior_end_of_premaxillary_body_(gives_the_posterior_process_a_forked_appearance_in_lateral_view) absent present;

{8 Maxillary_process_of_premaxilla contacts_nasal_to_form_posterior_border_of_nares reduced_so_that_maxilla_participates_broadly_in_external_naris extends_posteriorly_to_separate_maxilla_from_nasal__posterior_to_nares;

{9 Internarial_bar_ dorsoventrally_rounded dorsoventrally_flat;

{10 Crenulate_margin_on_buccal_edge_of_premaxilla_ absent present;

{11 Caudal_margin_of_naris farther_rostral_than_the_rostral_border_of_the_antorbital_fossa nearly_reaching_or_overlapping_the_rostral_border_of_the_antorbital_fossa;

{12 Premaxillary_symphysis_ acute,_V-shaped rounded,_U-shaped;

{13 Subnarial_foramen absent present;

{14 Groove_on_lateral_surface_of_premaxilla,_extending_ventrally_from_the_narial_fossa absent present;

{15 Maxillary_fenestra absent present;

{16 Maxillary fenestra recessed within a shallow, caudally or '^ncaudodorsally' open fossa, which is itself located within the maxillary antorbital fossa absent present;

{17 Longitudinal_position_of_maxillary_fenestra situated_at_rostral_border_of_antorbital_fossa situated_posterior_to_rostral_border_of_antorbital_fossa;

{18 Latitudinal_position_of_maxillary_fenestra situated_approximately_mid-height_of_the_antorbital_fossa displaced_dorsally_in_antorbital_fossa;

{19 Foramen_on_caudal_edge_interfenestral_bar_between_the_maxillary_and_antorbital_fenestrae absent present,_pierces_ventral_portion_of_bar;

{20 Promaxillary_fenestra_(fenestra_promaxillaris) absent present;

{21 Palate_formed_by premaxilla_only premaxilla,_maxilla_and_vomer;

{22 Palatal_shelf_of_maxilla_ flat with_midline_ventral_"tooth-like"_projection;

{23 Ventrolateral_margin_of_the_maxilla_posterior_to_ascending_process flat_or_rounded_as_it_grades_onto_tooth_row developed_as_a_sharp,_ventrolaterally-projecting_ridge;

{24 Anteroposterior_length_of_palatal_shelf_of_maxilla short long,_with_extensive_palatal_shelves;

{25 Orientation_of_the_maxillae_towards_each_other_as_seen_in_dorsal_view acutely_angled subparallel;

{26 Ascending_process_of_the_maxilla confluent_with_anterior_rim_of_maxillary_body_and_gently_sloping_posterodorsally offset_from_anterior_rim_of_maxillary_body;

{27 Form_of_anterior_projection_of_maxilla offset_from_anterior_rim_of_maxillary_body,_with_anterior_projection_of_maxillary_body_shorter_than_high offset_from_anterior_rim_of_maxillary_body,_with_anterior_projection_of_maxillary_body_as_long_as_high_or_longer;

{28 Ascending_process_of_maxilla prominent,_exposed_laterally_and_medially weakly_developed,_lacking_lateral_exposure_and_only_slight_medial_exposure;

{29 Anterior_margin_of_maxillary_antorbital_fossa rounded_or_pointed square;

{30 Dorsal_border_of_the_internal_antorbital_fenestra_lateral_view formed by lacrimal 'and^n' maxilla formed_by_nasal_and_lacrimal;

{31 Dorsal_border_of_the_antorbital_fossa_lateral_view formed by lacrimal 'and^n' maxilla formed_by_nasal_and_lacrimal formed_by_maxilla,_premaxilla_and_lacrimal;

{32 Lateral_exposure_of_lamina_of_the_ventral_ramus_of_nasal_process_of_maxilla present, large '^nbroad' exposure present,_reduced_to_small_triangular_exposure;

{33 Maxillary_antorbital_fossa_in_front_of_the_internal_antorbital_fenestra 40%_or_less_of_the_length_of_the_external_antorbital_fenestra more_than_40%_of_the_length_of_the_external_antorbital_fenestra;

{34 Extent_of_antorbital_fossa_on_jugal_ramus_of_maxilla less_than_half_the_dorsoventral_height_of_jugal_ramus more_than_half_dorsoventral_height_of_jugal_ramus;

{35 Maxilla,_pneumatic_region_on_medial_side_of_maxilla_posteroventral_to_maxillary_fenestra absent present;

{36 Horizontal_ridge_on_the_lateral_surface_of_maxilla_at_the_ventral_border_of_the_antorbital_fossa absent present;

{37 Medial_constriction_between_articulated_premaxillae_and_maxillae_in_dorsal_or_ventral_view absent present;

{38 Subnarial_gap_between_maxilla_and_premaxilla_at_the_alveolar_margin absent present;

{39 Maxillary_paradental_plates unfused fused;

{40 Medial_surface_of_maxillary_paradental_(interdental)_plates smooth_or_finely_pitted dorsoventrally_striated;

{41 Maxillary_paradental_(interdental)_plates,_ventral_extent to_the_same_ventral_level_as_lateral_maxillary_wall dorsal_to_ventral_level_of_maxillary_wall;

{42 Maxillary_paradental_plates,_dorsal_margin_of_anterior_end horizontal inclined_anteroventrally;

{43 Ventral_edge_of_maxillary_body_and_ventral_ramus ventrally_flat ventrally_convex;

{44 Nasals unfused fused;

{45 Dorsal_surface_of_the_nasals smooth rugose;

{46 Nasal_crest absent present,_single_median_crest present,_bilateral_crests_along_lateral_nasal_margins;

{47 Pneumatic_foramen_in_ventrolateral_margins_of__the_nasals absent present;

{48 Shape_of_nasals expanding_posteriorly of_subequal_width_throughout_their_length;

{49 Pronounced_lateral_rims_of_the_nasals,_sometimes_bearing_lateral_cranial_crests absent present;

{50 External_nares facing_laterally facing_anterolaterally;

{51 Length_of_nares less_than_20_percent_skull_length greater_than_20_percent_skull_length;

{52 Jugal_pneumatic_recess_in_posteroventral_corner_of_antorbital_fossa_ present absent;

{53 Medial_jugal_foramen present_on_medial_surface_ventral_to_postorbital_bar absent;

{54 Sublacrimal_part_of_jugal tapering bluntly_squared_anteriorly expanded;

{55 Anterior_end_of_jugal reaches_internal_antorbital_fenestra excluded_from_the_internal_antorbital_fenestra;

{56 Form_of_anterior_end_of_jugal without_anterior_process_underneath_antorbital_fenestra expressed_at_the_rim_of_the_internal_antorbital_fenestra_and_with_a_distinct_process_that_extends_anteriorly_underneath_it;

{57 Jugal_antorbital_fossa absent_or_developed_as_a_slight_depression large,_crescentic_depression_on_the_anterior_end_of_the_jugal;

{58 Jugal broad,_plate-like very_slender,_rod-like;

{59 Jugal_contribution_to_postorbital_bar contribute_equally_to_postorbital_bar ascending_process_of_jugal_reduced;

{60 Anteroposterior_width_of_postorbital_bar subequal_to_preorbital_bar expanded,_greater_than_twice_width_of_preorbital_bar;

{61 Rugosity_on_ventrolateral_surface_of_jugal_below_orbit absent present;

{62 Jugal_and_quadratojugal_ separate fused_and_not_distinguishable_from_one_another;

{63 Quadratojugal hook-shaped,_with_a_dorsoventrally_tall,_mediolaterally_short_process_that_wraps_around_the_lateral_margin_of_the_quadrate_and_is_visible_in_posterior_view with_a_dorsoventrally_short,_anteroposteriorly_long_process_only_visible_in_lateral_view;

{64 Quadratojugal_and_quadrate sutural_connection_present sutural_connection_absent;

{65 Anteriormost_level_of_jugal_process_of_quadratojugal_relative_to_infratemporal_fenestra ventral_to anterior_to;

{66 Supraorbital_crests_on_lacrimal_in_adult_individuals absent present;

{67 Form_of_supraorbital_crests dorsal_crest_above_orbit lateral_expansion_anterior_and_dorsal_to_orbit;

{68 Enlarged_foramen_or_foramina_opening_laterally_at_the_angle_of_the_lacrimal absent present;

{69 Lacrimal_foramen_number single paired;

{70 Lacrimal_foramina exposed_laterally_ developed_within_a_pocket_formed_by_a_lateral_lacrimal_sheet_of_bone_and_a_rostrally_open_pocket_in_the_lacrimal_angle;

{71 Height_of_the_lacrimal significantly_less_than_height_of_the_orbit,_and_usually_fails_to_reach_the_ventral_margin_of_the_orbit as_high_as_the_orbit,_and_contacts_jugal_at_the_level_of_the_ventral_margin_of_orbit;

{72 Orientation_of_jugal_ramus_of_lacrimal strongly_sloping_anteroventrally subvertical sloping_posteroventrally;

{73 Dorsoventral_thickness_of_maxillary_ramus_of_lacrimal very_slender,_much_less_than_anteroposterior_thickness_of_jugal_ramus moderate,_less_than_or_subequal_to_anteroposterior_thickness_of_jugal_ramus greater_than_anteroposterior_thickness_of_jugal_ramus;

{74 Suborbital_spur_on_posterior_edge_of_ventral_ramus_of_lacrimal absent present;

{75 Lacrimal_posterodorsal_process absent present;

{76 Length_of_lacrimal_posterodorsal_process subequal_in_length_to_maxillary_ramus much_shorter_than_maxillary_ramus;

{77 Direction_of_lacrimal_posterodorsal_process projects_horizontally projects_posterodorsally_or_completely_dorsally;

{78 Passage_of_the_nasolacrimal_duct leading_through_the_body_of_the_ventral_process_of_the_lacrimal ventral_process_of_lacrimal_not_pierced,_lateral_side_depressed_below_the_level_of_the_surrounding_bones,_and_nasolacrimal_duct_passes_lateral_to_the_process;

{79 Jugal_ramus_of_lacrimal broadly_triangular,_articular_end_nearly_twice_as_wide_anteroposteriorly_as_lacrimal_body_at_lacrimal_angle strut-like,_roughly_same_width_anteroposteriorly_throughout_ventral_ramus;

{80 Prefrontal absent present;

{81 Size_of_prefrontal small,_forms_anterolateral_rim_of_orbit_with_descending_process_proceeding_along_medial_surface_of_the_descending_process_of_the_lacrimal small,_forms_small_portion_of_skull_roof_and_not_expressed_at_orbital_margin,_no_descending_process hypertrophied,_forms_portion_of_orbital_rim_and_skull_roof,_with_descending_process;

{82 Configuration_of_lacrimal_and_frontal lacrimal_separated_from_frontal_by_prefrontal lacrimal_contacts_frontal;

{83 Frontals narrow_anteriorly_as_a_wedge_between_nasals end_abruptly_anteriorly,_suture_with_nasal_transversely_oriented nasals_extend_further_medially_than_laterally,_invading_anteromedial_contact_between_frontals;

{84 Frontal_supratemporal_fossa limited_extension_of_supratemporal_fossa_onto_frontal supratemporal_fossa_coovers_most_of_postorbital_process_of_the_frontal_and_extends_anteriorly_onto_the_dorsal_surface_of_the_frontal;

{85 Groove_on_orbital_rim_of_frontal,_possibly_for_reception_of_frontal_process_of_postorbital absent present;

{86 Anterior_emargination_of_supratemporal_fossa_on_frontal_ straight_or_slightly_curved strongly_sinusoidal_and_reaching_onto_postorbital_process;

{87 Frontal_postorbital_process_(dorsal_view):_ smooth_transition_from_orbital_margin sharply_demarcated_from_orbital_margin;

{88 Orbital_margin_of_frontal without_groove with_groove_for_articulation_with_frontal_process_of_the_postorbital;

{89 Frontal_edge smooth_in_region_of_lacrimal_suture edge_notched;

{90 Postorbital_in_lateral_view_ with_straight_anterior_(frontal)_process frontal_process_curves_anterodorsally_and_dorsal_border_of_temporal_bar_is_dorsally_concave;

{91 Lateral_surface_of_anterior_process_of_postorbital thin_and_unornamented dorsoventrally_thickened_into_a_laterally_projecting_and_rugose_platform;

{92 Contact_between_lacrimal_and_postorbital absent present;

{93 Cross-section_of_the_ventral_process_of_the_postorbital triangular U-shaped;

{94 Jugal_process_of_the_postorbital ventrally_directed_and_tapering with_suborbital_anterior_spur;

{95 Postorbital_jugal_process_anterior_suborbital_spur small large_curving_flange;

{96 Supraorbital_shelf_formed_mostly_by_an_additional_ossification_(palpebral) absent present;

{97 Orbit circular_in_lateral_or_dorsolateral_view dorsoventrally_long;

{98 Parietals separate fused;

{99 Parietal_supratemporal_fenestra separated_by_a_horizontal_plate_formed_by_the_parietals contact_each_other_posteriorly,_but_separated_anteriorly_by_an_anteriorly_widening_triangular_plate_formed_by_the_parietals nearly_confluent_over_parietals_and_only_separated_by_a_thin_line_of_bone_along_the_sagittal_suture;

{100 Anteromedial_corner_of_supratemporal_fossa open_dorsally roofed_by_shelf_of_frontoparietal;

{101 Sagittal_crest dorsal_surface_of_parietals_smooth_with_no_sagittal_crest sagittal_crest_present;

{102 Form_of_sagittal_crest parietals_dorsally_convex_with_very_low_sagittal_crest_along_midline dorsally_convex_with_well-developed_sagittal_crest;

{103 Posteriorly_placed,_knob-like_dorsal_projection_of_the_parietals absent present;

{104 Connections_of_quadratojugal_process_of_squamosal contacts_quadratojugal does_not_contact_quadratojugal;

{105 Infratemporal_fenestra_shape rectangular,_postorbital_bar_parallels_quadratojugal_and_squamosal_articular_area lower_temporal_fenestra_constricted_mesially_by_squamosal_and_quadratojugal_approaching_postorbital_bar;

{106 Shape_of_quadratojugal_process_of_the_squamosal tapering broad,_and_usually_somewhat_expanded;

{107 Posterolateral_shelf_on_squamosal_overhanging_quadrate_head_ absent present;

{108 Quadrate_head covered_by_squamosal_in_lateral_view quadrate_cotyle_of_squamosal_open_laterally_exposing_quadrate_head;

{109 Descending_process_of_squamosal_ parallels_quadrate_shaft nearly_perpendicular_to_quadrate_shaft;

{110 Supratemporal_fenestra bounded_laterally_and_posteriorly_by_the_squamosal supratemporal_fenestra_extended_as_a_fossa_on_to_the_dorsal_surface_of_the_squamosal;

{111 Quadrate solid hollow;

{112 Mandibular_joint approximately_straight_below_quadrate_head significantly_posterior_to_quadrate_head significantly_anterior_to_quadrate_head;

{113 Quadrate_medial_pneumatic_recess_(depression_and_foramen_in_the_area_of_the_mandibular_condyle_on_medial_surface) absent fossa_adjacent_to_mandibular_condyle,_foramen_at_base_of_pterygoid_ramus;

{114 Quadrate_posterior_pneumatic_recess absent present_as_a_lens-shaped_fossa_extending_dorsally_or_dorsomedially_from_the_quadrate_foramen;

{115 Dorsal_end_of_the_quadrate with_a_single_head_that_fits_into_a_slot_on_the_ventral_side_of_the_squamosal double-headed,_medial_head_contacts_the_braincase;

{116 Quadrate_foramen absent present;

{117 Quadrate_foramen developed_as_a_distinct_opening_between_the_quadrate_and_quadratojugal almost_entirely_closed_in_the_quadrate;

{118 Ectopterygoid slender,_without_ventral_fossa expanded,_with_a__ventral_depression_medially expanded,_with_a_deep_groove_leading_into_the_ectopterygoid_body_medially deeply_excavated_and_medial_opening_constricted_into_a_foramen;

{119 Dorsal_recess_on_ectopterygoid_ absent present;

{120 Ectopterygoid posterior_to_palatine lateral_to_palatine;

{121 Palatine_and_ectopterygoid separated_by_pterygoid contact;

{122 Contact_between_pterygoid_and_palatine continuous discontinuous_in_the_mid-region,_resulting_in_a_subsidiary_palatal_fenestra;

{123 Flange_of_pterygoid_ well_developed reduced_in_size_or_absent;

{124 Shape_of_palatine_in_ventral_view plate-like_trapezoidal_or_subrectangular tetraradiate jugal_process_strongly_reduced_or_absent;

{125 Suborbital_fenestra_ similar_in_length_to_orbit reduced_in_size_or_absent;

{126 Infratemporal_fenestra smaller_than_or_subequal_in_size_to_orbit strongly_enlarged,_more_than_1.5_times_the_size_of_the_orbit;

{127 Postorbital_part_of_the_skull_roof as_high_as_orbital_region deflected_ventrally_in_adult_individuals;

{128 Preorbital_region_of_the_skull_in_post-hatchling_individuals elongate,_nasals_considerably_longer_than_frontals,_maxilla_at_least_twice_the_length_of_the_premaxilla shortened,_nasals_subequal_in_length_to_frontals_or_shorter,_maxillary_length_less_than_twice_the_length_of_the_premaxillary_body;

{129 Occipital_region_of_the_skull_faces posteriorly posteroventrally;

{130 Basipterygoid_processes well-developed,_extending_as_a_distinct_process_from_the_base_of_the_basisphenoid abbreviated_or_absent;

{131 Basipterygoid_processes_well_developed_and anteroposteriorly_short_and_finger-like_(approximately_as_long_as_wide) longer_than_wide significantly_elongated_and_tapering;

{132 Basipterygoid_processes ventral_or_anteroventrally_projecting lateroventrally_projecting caudally_projecting;

{133 Basipterygoid_processes solid hollow;

{134 Basipterygoid_recesses_on_dorsolateral_surfaces_of_basipterygoid_processes_ absent present;

{135 Basisphenoid_bulla absent present;

{136 Basisphenoid_recess absent_or_poorly_developed deep_and_well-developed;

{137 Passage_of_internal_carotids_between_posterior_end_of_skull_and_pituitary_fossa no_bony_tubes_present enclosed_by_bony_tubes_extending_along_ventral_surface_of_basisphenoid;

{138 Basisphenoid_recess_position between_basisphenoid_and_basioccipital entirely_within_basisphenoid;

{139 Posterior_opening_of_basisphenoid_recess_ single divided_into_two_small,_circular_foramina_by_a_thin_bar_of_bone;

{140 Basisphenoid_between_basal_tubera_and_basipterygoid_processes approximately_as_wide_as_long,_or_wider significantly_elongated,_at_least_1.5_times_longer_than_wide;

{141 Basisphenoid_in_lateral_view oriented_subhorizontally anterior_portion_located_much_more_ventrally_than_posterior_portion,_recess_visible_in_posterior_view;

{142 Base_of_cultriform_process_ not_highly_pneumatised expanded_and_pneumatic_(parasphenoid_bulba);

{143 Vestibular_and_Cochlear_branches_of_CN_VIII;

{144 Exits_of_CN_X-XII flush_with_surface_of_exoccipital located_together_in_a_bowl-like_basisphenoid_depression;

{145 Exits_of_CN_X_and_XI laterally_through_the_jugular_foramen posteriorly_through_a_foramen_(metotic_foramen)_lateral_to_the_exit_of_cranial_nerve_XII_and_the_occipital_condyle;

{146 Exoccipital_lateral_to_occipital_condyle forms_roof_over_exits_for_CN_X_and_XII unexpanded_and_does_not_form_roof;

{147 Supraoccipital_sagittal_crest with_pronounced_sagittal_crest sagittal_crest_reduced_or_absent;

{148 Paroccipital_process_shape_ elongate_and_slender short,_deep_;

{149 Paroccipital_process_direction straight,_projects_laterally_or_posterolaterally project_ventrolaterally pendant;

{150 Paroccipital_process_dorsal_edge with_straight_dorsal_edge distal_end_twists_rostrally,_distal_ends_of_the_processes_oriented_transversely_rather_than_vertically;

{151 Ventral_rim_of_the_basis_of_the_paroccipital_processes above_or_level_with_the_dorsal_border_of_the_occipital_condyle situated_at_mid-height_of_occipital_condyle_or_lower;

{152 Foramen_magnum subcircular,_slightly_wider_than_tall oval,_taller_than_wide;

{153 Foramen_magnum_size smaller_than_or_subequal_in_width_to_occipital_condyle larger_in_width_than_occipital_condyle;

{154 Occipital_condyle without_constricted_neck subspherical_with_constricted_neck;

{155 Infracondylar_fossa_of_occipital_condyle absent present;

{156 Form_of_infracondylar_fossa_of_occipital_condyle narrow_and_groove-like broad_depression_approximately_two-thirds_the_width_of_the_occipital_condyle;

{157 Basal_tubera present absent;

{158 Basal_tubera_composition equally_formed_by_basioccipital_and_basisphenoid_and_not_subdivided subdivided_by_a_lateral_longitudinal_groove_into_a_medial_part_entriely_formed_by_the_basioccipital,_and_a_lateral_part,_entirely_formed_by_the_basisphenoid;

{159 Basal_tubera_spacing set_far_apart,_level_with_or_beyond_lateral_edge_of_occipital_condyle_and/or_foramen_magnum tubera_small,_directly_below_condyle_and_foramen_magnum,_and_separated_by_a_narrow_notch;

{160 Subcondylar_recess absent present_in_basioccipital/exoccipital_lateral_and_ventral_to_occipital_condyle;

{161 Subcondylar_recess_form isolated_from_nervous_foramina_CNX-CNXII subcondylar_recess_and_cranial_nerves_exit_together_in_a_deep_depression_encompassing_multiple_pneumatic_fossae_and_enclosed_by_a_well_developed_rim;

{162 Exit_of_mid-cerebral_vein included_in_trigeminal_foramen vein_exits_braincase_through_a_separate_foramen_anterodorsal_to_the_trigeminal_foramen;

{163 Brain_proportions forebrain_small_and_narrow forebrain_significantly_enlarged_and_triangular;

{164 Anterior_tympanic_recess_in_the_braincase absent present;

{165 Prootic_pneumatic_recess absent present;

{166 Form_of_pneumatic_prootic_recess dorsally_open_fossa_on_prootic/opisthotic deep,_posterolaterally_directed_concavity;

{167 Crista_interfenestralis_ confluent_with_lateral_surface_of_prootic_and_opisthotic distinctly_depressed_within_middle_ear_opening;

{168 Accessory_dorsal_tympanic_recess_(dorsal_to_crista_interfenestralis)_ absent present;

{169 Form_of_dorsal_tympanic_recess small_pocket_present extensive_with_indirect_pneumatisation;

{170 Caudal_(posterior)_tympanic_recess_ absent present;

{171 Form_of_caudal_tympanic_recess present_as_opening_on_anterior_surface_of_paroccipital_process extends_into_opisthotic_posterodorsal_to_fenestra_ovalis,_confluent_with_this_fenestra;

{172 Exoccipitals_ventral_to_posterior_pneumatic_recess no_lip form_anteriorly_projecting,_posterodorsally_curling,_dorsally_concave,_tablike_process;

{173 Otosphenoidal_crest_ vertical_on_basisphenoid_and_prootic,_and_does_not_border_an_enlarged_pneumatic_recess well-developed,_crescent-shaped,_thin_crest_forms_anterior_edge_of_enlarged_pneumatic_recess;

{174 Subotic_recess_(pneumatic_fossa_ventral_to_fenestra_ovalis)_ absent present;

{175 Depression_(possibly_pneumatic)_on_ventral_surface_of_postorbital_process_of_laterosphenoid absent present;

{176 Interorbital_region_in_adults unossified ossified;

{177 Prominent_endocranial_expansion_of_vertical_semicircular_canal absent present;

{178 Mandibular_foramen absent_or_reduced large hypertrophied,_greater_than_50%_dentary_length;

{179 Shape_of_mandibular_foramen oval subdivided_by_a_spinous_rostral_process_of_the_surangular;

{180 Paradental_plates_of_dentary lack_paradental_plates with_paradental_plates_on_the_medial_surface_of_the_tooth_row;

{181 Internal_mandibular_fenestra small_and_slit-like large_and_rounded;

{182 Shape_of_anterior_end_of_dentary blunt_and_unexpanded dorsoventrally_expanded,_rounded_and_slightly_upturned with_anteroventral_process_giving_a_"squared_off"_appearance_in_lateral_view;

{183 Dorsal_edge_of_anterior_end_of_dentary_in_lateral_view dorsally_flat with_dorsally_expanded,_arcuate_eminence;

{184 Symphyseal_region_of_dentary Broad_and_straight,_paralleling_lateral_margin medially_recurved;

{185 Degree_of_medial_recurvature_of_dentary_symphysis medially_recurved_slightly strongly_recurved_medially;

{186 Dentary_symphyseal_fusion absent present;

{187 Dentary_anterior_end_in_lateral_view in_line_with_main_part_of_buccal_edge anterior_end_deflected_ventrally;

{188 Width_of_dentary_symphyseal_region no_broader_than_transverse_width_of_post-symphyseal_region broader_than_post-symphyseal_region;

{189 Orientation_of_dentary_symphysis_in_lateral_view vertical_to_subvertical projects_strongly_cranially,_oblique_with_respect_to_dentary_ventral_margin;

{190 Posterior_end_of_dentary without_posterodorsal_process_dorsal_to_mandibular_fenestra with_dorsal_process;

{191 Form_of_dentary_posterodorsal_process developed_only_above_anterior_end_of_mandibular_fenestra with_elongate_dorsal_process_extending_over_most_of_fenestra;

{192 Labial_face_of_dentary_ flat with_lateral_ridge_and_inset_tooth_row;

{193 Nutrient_foramina_on_external_surface_of_dentary_ superficial descend_strongly_posteriorly_within_a_deep_groove;

{194 Form_of_nutrient_foraminal_groove thin_groove_of_constant_height_as_it_extends_posteriorly posterior_end_of_groove_is__dorsoventrally_expanded;

{195 Dentary_shape_in_lateral_view with_subparallel_dorsal_and_ventral_edges subtriangular_in_lateral_view;

{196 Form_of_triangular_dentary low_triangular high_triangular;

{197 Ventral_edge_of_dentary_in_lateral_view straight_or_nearly_straight descends_strongly_posteriorly;

{198 Dentary_paradental_groove_separating_interdental_plates_from_medial_wall_of_dentary absent present;

{199 Pronounced_coronoid_eminence_on_the_surangular absent present;

{200 Foramen_in_lateral_surface_of_surangular_rostral_to_mandibular_articulation absent present;

{201 Number_of_surangular_foramina one two;

{202 Laterally_inclined_flange_along_dorsal_edge_of_surangular_for_articulation_with_lateral_process_of_lateral_quadrate_condyle absent present;

{203 Anterior_portion_of_the_surangular less_than_half_the_height_of_the_mandible_above_the_mandibular_fenestra more_than_half_the_height_of_the_mandible_at_the_level_of_the_mandibular_fenestra;

{204 Retroarticular_process_of_the_mandible narrow,_rod-like broadened,_with_groove_posteriorly_for_the_attachment_of_the_m._depressor_mandibulae;

{205 Attachment_of_the_m._depressor_mandibulae_on_retroarcticular_process_of_mandible facing_dorsally facing_posterodorsally;

{206 Retroarticular_process points_posteriorly curves_gently_posterodorsally;

{207 Articular_ without_elongate,_slender_medial,_posteromedial,_or_mediodorsal_process_from_retroarticular_process with_process;

{208 Angular exposed_almost_to_end_of_mandible_in_lateral_view,_reaches_or_almost_reaches_articular excluded_from_posterior_end_angular_suture_turns_ventrally_and_meets_ventral_border_of_mandible_rostral_to_glenoid;

{209 Coronoid_ossification absent present;

{210 Form_of_coronoid_ossification large thin_splint;

{211 Splenial not_widely_exposed_on_lateral_surface_of_mandible exposed_as_a_broad_triangle_between_dentary_and_angular_on_lateral_surface_of_mandible;

{212 Foramen_in_the_ventral_part_of_the_splenial_(mylohyal_foramen) absent present;

{213 Form_of_mylohyal_foramen completely_enclosed_in_the_splenial opened_anteroventrally;

{214 Posterior_end_of_splenial straight forked;

{215 Articular_glenoid_fossa as_long_as_distal_end_of_quadrate twice_or_more_as_long_as_quadrate_surface,_allowing_anteroposterior_movement_of_mandible;

{216 Palatal_teeth present absent;

{217 Premaxillary_teeth present absent;

{218 Number_of_premaxillary_teeth three four five more_than_five;

{219 First_premaxillary_tooth_size slightly_smaller_or_the_same_size_as_2_and_3 much_smaller_than_2_and_3 much_larger_than_2_and_3;

{220 Second_premaxillary_tooth approximately_equivalent_in_size_to_other_premaxillary_teeth markedly_larger_than_third_and_fourth_premaxillary_teeth;

{221 Premaxillary_tooth_direction decumbent_or_ventrally_projecting procumbent;

{222 Serrations_on_premaxillary_teeth present absent;

{223 In_cross_section,_premaxillary_tooth_crowns sub-oval_to_sub-circular D-shaped_with_flat_lingual_surface;

{224 Maxillary_teeth present absent;

{225 Length_of_maxillary_tooth_row extends_posteriorly_to_approximately_half_the_length_of_the_orbit ends_at_the_anterior_rim_of_the_orbit completely_antorbital,_tooth_row_ends_anterior_to_the_vertical_strut_of_the_lacrimal ends_below_the_junction_between_the_maxillary_body_and_the_ascending_process;

{226 Number_of_maxillary_teeth 10-14 15-19 20_or_more;

{227 Maxillary_tooth_direction ventrally_or_posteriorly_inclined procumbent;

{228 Maxillary_and_dentary_teeth,_mesial_(anterior)_carina present absent;

{229 Mesial_(anterior)_carina_of_maxillary_and_dentary_teeth_present_and extends_to_base_of_crown terminates_ventrally_at_approximately_mid-crown_level_or_more_dorsally;

{230 Shape_of_maxillary_teeth mediolaterally_flattened,_dorsoventrally_taller_than_anteropostiorly_wide lanceolate_and_subsymmetrical_(as_in_therizinosaurs) simple,_conical,_incisive_crowns_(as_in_Alvarezsaurs)_;

{231 Degree_of_curvature_of_maxillary_tooth_crowns crowns_curve_posteriorly_as_they_extend_distally very_little_curvature_or_crowns_straight;

{232 Serrations_on_maxillary_and_dentary_teeth_ present some_without_serrations_anteriorly absent;

{233 Maxillary_tooth_implantation separate_alveoli set_in_an_open_groove;

{234 Roots_of_maxillary_and_dentary_teeth mediolaterally_compressed circular_in_cross-section;

{235 Dentary_tooth_row fully_toothed only_teeth_rostrally edentulous fully_toothed_with_short_edentulous_anterior_portion;

{236 Number_of_dentary_teeth large,_fewer_than_25_in_dentary moderate_number_of_small_teeth_(25-30_in_dentary) relatively_small_and_numerous_(more_than_30_in_dentary);

{237 Dentary_teeth_distribution homodont increasing_in_size_anteriorly,_becoming_more_conical_in_shape Decreasing_in_size_anteriorly,_becoming_more_densely_packed;

{238 Shape_of_dentary_teeth mediolaterally_flattened,_dorsoventrally_taller_than_anteroposteriorly_wide lanceolate_and_subsymmetrical_(as_in_therizinosauroids) simple,_conical,_incisive_crowns_(as_in_Alvarezsaurs)_;

{239 Third_dentary_alveolus subequal_in_size_to_other_alveoli circular_and_enlarged_relative_to_other_alveoli;

{240 Dentary_tooth_implantation separate_alveoli set_in_an_open_groove;

{241 Dentary_tooth_direction dorsally_or_posteriorly_inclined procumbent_(anteriorly_inclined);

{242 Serrations_on_maxillary_and_dentary_teeth simple,_denticles_convex distal_and_often_mesial_edges_of_teeth_with_large,_hooked_denticles_that_point_toward_the_tip_of_the_crown;

{243 Serration_size large small;

{244 Constriction_between_tooth_crown_and_root absent present;

{245 Enamel_of_tooth_crowns smooth horizontally_wrinkled,_especially_flanking_the_serrations;

{246 Form_of_enamel_wrinkles bands_extending_across_labial_and_lingual_tooth_surfaces adjacent_to_carinae_but_do_not_extend_across_labial_and_lingual_tooth_surfaces;

{247 Vertical_striations_of_enamel_of_tooth_crowns absent present;

{248 Axial_diapophyses moderate reduced_or_absent;

{249 Axial_parapophyses prominent_or_moderate reduced_or_absent;

{250 Axial_neural_spine flared_transversely_and_sheet-like compressed_mediolaterally,_anteroposteriorly_reduced,_and_rodlike;

{251 Epipophyses_on_axis absent present;

{252 Form_of_axial_epipophyses present_as_small_ridges strongly_pronounced_(overhanging_the_zygapophyses);

{253 Pleurocoel_in_axis absent present;

{254 Number_of_cervical_vertebrae 10 More_than_10;

{255 Pleurocoels_in_cervical_vertebrae absent present;

{256 Number of pleurocoels in 'cervicals^n^n''' one two;

{257 Arrangement_of_two_foramina_in_cortical_surface_of_cervical_centra one_in_anterior_half_of_lateral_surface,_one_in_posterior_half both_foramina_in_anterior_half;

{258 Pleurocoels_developed_as deep_depressions foramina;

{259 Interior_pneumatic_spaces_in_cervicals Structure_camerate_(few_chambers) Structure_camellate_(many_chambers_separated_by_delicate_lamellae);

{260 Ventral_surface_of_anterior_cervicals keeled smooth ventral_depression;

{261 Posterolateral_crests_on_lateral_surfaces_of_cervical_centra absent present;

{262 Anterior_cervical_centra_length less_than_twice_transverse_centrum_width between_two_and_three_time_transverse_width three_to_five_times_transverse_width;

{263 Anterior_articular_facet_of_anterior_cervical_vertebrae approximately_as_high_as_wide_or_higher significantly_wider_than_high wider_than_high_and_higher_laterally_than_medially_(kidney-shaped),_with_neural_canal_emarginating_dorsal_aspect;

{264 Anterior_cervical_centra_relative_length_ level_with_or_shorter_than_posterior_extent_of_neural_arch centra_extending_beyond_posterior_limit_of_neural_arch;

{265 Articulation_surfaces_of_cervical_centra amphi-_to_platycoelus opisthocoelus heterocoelus;

{266 Carotid_process_on_posterior_cervical_centra absent present;

{267 Epipophyses_in_anterior_cervical_vertebrae absent_or_poorly_developed well-developed;

{268 Form_of_well-developed_cervical_epipophyses proximal_to_postzygapophyseal_facets strongly_overhanging_postzygapophyseal_facets;

{269 Prezygapophyseal-epipophyseal_lamina_on_dorsal_surface_of_neural_arch absent_or_poorly_developed extending_anteriorly_from_epipophysis_as_a_mediolaterally_thin_ridge_that_separates_dorsal_surface_of_diapophysis_from_rest_of_dorsal_neural_arch;

{270 Postzygapophyses_of_cervical_vertebrae_2-4 well-separated,_or_connected_only_at_the_base medially_connected_along_their_entire_length_by_a_intrazygapophyseal_lamina_that_is_dorsally_concave_for_attachment_of_the_interspinous_ligaments;

{271 Cervical_neural_spines_ anteroposteriorly_long anteroposteriorly_short_and_centered_on_neural_arch,_giving_arch_an_"X"_shape_in_dorsal_view extremely_short_anteroposteriorly,_less_than_1/3_length_of_neural_arch;

{272 Cervical_neural_spine_height dorsoventrally_tall,_subequal_to_or_exceeding_height_of_neural_arch_from_centrum_to_base_of_neural_spine moderate,_less_than_neural_arch_height strongly_reduced,_less_than_half_height_of_neural_arch_(not_including_spine_itself);

{273 Prezygapophyses_in_anterior_cervicals transverse_distance_between_prezygapophyses_less_than_width_of_neural_canal prezygapophyses_situated_lateral_to_the_neural_canal;

{274 Prezygapophyses_in_anterior_postaxial_cervicals straight anteroposteriorly_convex,_flexed_ventrally_anteriorly;

{275 Pneumaticity_of_dorsal_neural_arches absent_to_moderate extreme;

{276 Hypapophyses_in_anterior_dorsals absent_or_poorly_developed pronounced;

{277 Pleurocoels_in_dorsal_vertebrae absent present_in_anterior_dorsals_('pectorals') present_in_all_dorsals;

{278 Dorsal_centra_articular_surfaces amphiplatyan opisthocoelus;

{279 Ventral_keel_in_anterior_dorsals absent_or_very_poorly_developed pronounced;

{280 Shape_of_dorsal_centra_in_anterior_view subcircular_or_oval significantly_wider_than_high triangular;

{281 Posterior_dorsal_vertebrae strongly_shortened,_centra_much_shorter_than_high relatively_short,_centra_approximately_as_high_as_long,_or_only_slightly_longer significantly_elongated,_much_longer_than_high;

{282 Posterior_dorsal_vertebrae,_basal_webbing_of_neural_spines absent present;

{283 Posterior_dorsal_vertebrae,_orientation_of_neural_spines vertically_or_posteriorly anteriorly;

{284 Anterior_dorsal_vertebrae_height_of_prezygadiapophyseal_lamina less_than_or_subequal_to_height_of_centrum hypaxially_inflated,_height_significantly_greater_than_centrum_height;

{285 Anterior_dorsal_vertebrae,_anterior_and_posterior_infrazygapophyseal_fossae single with_one_or_more_accessory_centrodiapophyseal_laminae_dividing_fossa_into_multiple_chambers;

{286 Transverse_processes_of_anterior_dorsal_vertebrae subhorizontal_to_vertically_inclined pendant;

{287 Parapophyseal_facets_of_anterior_dorsal_vertebrae moderate_in_size_(less_than_half_height_of_centrum) hypertrophied_(greater_than_two_thirds_centrum_height);

{288 Hyposphene-hypantrum_articulation_in_dorsal_vertebrae absent present;

{289 Step-like_ridge_lateral_to_hyposphene_running_posterodorsally_from_the_dorsal_border_of_the_neural_canal_to_the_posterior_edge_of_the_postzgyapophyses_of_dorsal_vertebrae_(visible_in_lateral_view) absent present;

{290 Postzygapophyses_of_the_dorsal_vertebrae_in_posterior_view without_lateral_flanges with_lateral,_small,_flange-like_lateral_extensions_of_postzygapophyseal_facets;

{291 Postzygapophyses_of_dorsal_vertebrae abutting_one_another_above_neural_canal,_opposite_hyposphenes_meet_to_form_lamina zygapophyses_placed_lateral_to_neural_canal_and_separated_by_groove_for_interspinous_ligaments,_hyposphenes_separated;

{292 Neural_spines_on_posterior 'rectangular_or_square' / anteroposteriorly_expanded_distally,_fan-shaped;

{293 Neural_spines_of_dorsal_vertebrae_in_dorsal_view not_expanded_distally expanded_laterally_in_dorsal_view_to_form_"spine_table";

{294 Scars_for_interspinous_ligaments terminate_at_apex_of_neural_spine_in_dorsal_vertebrae terminate_below_apex_of_neural_spine;

{295 Neural_spines_of_posterior_dorsals broadly_rectangular_and_approximately_as_dorsoventrally_high_as_anteroposteriorly_long high_rectangular,_significantly_dorsoventrally_higher_than_anteroposteriorly_long;

{296 Hook-like_extension_on_anterior_end_of_dorsal_neural_spines_in_lateral_view absent present_(with_associated_depression_immediately_caudal_to_the_projection_for_spinous_ligament_attachment);

{297 Parapophyses_of_posterior_dorsal_vertebrae flush_with_neural_arch distinctly_projected_on_pedicels;

{298 Parapophyses_in_posteriormost_dorsals on_same_level_as_transverse_process distinctly_below_transverse_process;

{299 Transverse_processes_of_anterior_dorsal_vertebrae proximodistally_long_and_anteroposteriorly_thin proximodistally_short,_anteroposteriorly_wide;

{300 Notarium_of_dorsal_vertebrae absent present;

{301 Number_of_sacral_vertebrae two three four five six seven eight nine_or_more;

{302 Pleurocoels_in_centra_of_sacral_vertebrae absent present_on_anterior_sacrals_only present_on_all_sacrals;

{303 Ventral_surface_of_posterior_sacral_centra gently_rounded,_convex flattened_ventrally,_sometimes_with_shallow_sulcus centrum_strongly_constricted_transversely,_ventral_surface_keeled;

{304 Transverse_dimensions_of_mid-sacral_centra_relative_to_other_sacral_centra subequal mediolaterally_narrower mediolaterally_wider;

{305 Sacral_vertebrae with_unfused_zygapophyses with_fused_zygapophyses_forming_a_sinuous_ridge_in_dorsal_view;

{306 Last_sacral_centrum_ with_flat_posterior_articulation_surface convex_articulation_surface;

{307 Fenestrae_between_neural_spines_of_sacral_vertebrae present absent;

{308 Sacral_ribs slender_and_well-separated forming_a_more_or_less_continuous_sheet_in_ventral_or_dorsal_view very_massive_and_strongly_expanded;

{309 Sacral_neural_arch_pneumaticity absent_to_moderate extreme;

{310 Number_of_caudal_vertebrae more_than_40 25-40 fewer_than_25;

{311 Pygostyle absent present,_centra_of_distal_caudal_vertebrae_fused;

{312 Pleurocoels_in_centra_of_anterior_caudal_vertebrae absent present;

{313 Caudal_centra amphiplatyan procoelus;

{314 Shape_of_anterior_caudal_centra oval subrectangular_and_box-like laterally_compressed_with_a_ventral_keel;

{315 Ventral_surface_of_anterior_caudals rounded with_a_distinct_keel_sometimes_bearing_a_narrow,_shallow_groove_on_its_midline grooved;

{316 Relative_length_of_distal_caudal_centra significantly_elongated_in_relation_to_centrum_height not_elongated_in_relation_to_centrum_height;

{317 Caudal_vertebrae with_distinct_transition_point,_from_shorter_centra_with_long_transverse_processes_proximally_to_longer_centra_with_small_or_no_transverse_processes_distally homogeneous_in_shape,_with_no_transition_point;

{318 Position_of_transition_point distal_to_the_tenth_caudal_vertebra between_the_7th_and_10th_caudal_vertebrae proximal_to_the_7th_caudal_vertebra;

{319 Location_of_transverse_processes_of_proximal_caudals centrally_positioned_on_centrum anteriorly_displaced;

{320 Centrodiapophyseal_laminae_of_anterior_caudal_vertebrae weak prominent,_as_well_developed_as_those_of_dorsal_vertebrae;

{321 Neural_spines_on_distal_caudals form_a_low_ridge spine_absent midline_sulcus_in_center_of_the_neural_arch;

{322 Neural_spines_of_caudal_vertebrae simple,_undivided separated_into_anterior_and_posterior_alae_throughout_much_of_caudal_sequence;

{323 Neural_spines_of_mid-caudals rod-like_and_posteriorly_inclined rod-like_and_vertical subrectangular_and_sheet-like;

{324 Prezygapophyses_of_distal_caudal_vertebrae between_1/3_and_whole_centrum_length with_extremely_long_extensions_of_the_prezygapophyses_(up_to_10_vertebral_segments_in_some_taxa) strongly_reduced_as_in_Archaeopteryx_lithographica;

{325 Anterior_margin_of_neural_spines_of_anterior_mid-caudal_vertebrae straight with_distinct_kink,_dorsal_part_of_anterior_margin_more_strongly_inclined_posteriorly_than_ventral_part;

{326 Long,_hair-like_cervical_ribs absent present;

{327 Shaft_of_cervical_ribs slender_and_longer_than_vertebra_to_which_they_articulate broad_and_shorter_than_vertebra;

{328 Posterior_cervical_ribs_and_centra separate fused;

{329 Ossified_uncinate_processes absent present;

{330 Ossified_sternal_ribs absent present;

{331 Lateral_gastral_segment shorter_than_medial_one_in_each_arch distal_segment_longer_than_proximal_segment;

{332 Cranial_process_at_base_of_chevrons absent present;

{333 Proximal_surface_of_chevrons distinct_transverse_ridge_dividing_surface_into_anterior_and_posterior_facets no_ridge,_low_mounds_may_be_present_laterally;

{334 Proximal_end_of_chevrons_of_proximal_caudals_ short_anteroposteriorly,_shaft_cylindrical proximal_end_elongate_anteroposteriorly,_flattened_and_plate-like;

{335 Mid-caudal_chevrons rod-like_or_only_slightly_expanded_ventrally L-shaped;

{336 Distal_chevrons rod-like_or_L-shaped skid-like;

{337 Distal_caudal_chevrons_ simple anteriorly_bifurcate bifurcate_at_both_ends;

{338 Ossified_sternal_plates separate_in_adults fused;

{339 Ventral_keel_on_sternum absent present;

{340 Sternum without_distinct_lateral_xiphoid_process_posterior_to_costal_margin with_lateral_xiphoid_process;

{341 Furcula absent present;

{342 Furcula_shape v-shaped u-shaped,_with_bowed_epicleidea;

{343 Hypocleidium_on_furcula absent present;

{344 Articular_facet_of_coracoid_on_sternum_(conditions_may_be_determined_by_the_articular_facet_on_coracoid_in_taxa_without_ossified_sternum anterolateral_or_more_lateral_than_anterior almost_anterior;

{345 Anterior_edge_of_sternum grooved_for_reception_of_coracoids without_grooves;

{346 Coracoid_in_lateral_view_ subcircular,_with_low_ventral_blade_and_no_or_small_posterior_process shallow_ventral_blade_with_elongate_posterior_process subquadrangular_with_extensive_ventral_blade strut-like,_very_tall_ventral_blade_with_little_or_no_posterior_process;

{347 Posterior_edge_of_coracoid not_or_shallowly_indented_below_glenoid posterior_edge_of_coracoid_deeply_notched_just_ventral_to_glenoid,_glenoid_lip_everted;

{348 External_surface_of_coracoid_ventral_to_glenoid_fossa_and_along_dorsal_margin_of_posterventral_blade unexpanded expanded,_forms_triangular_subglenoid_fossa_bounded_laterally_by_coracoid_tuber;

{349 Coracoid_tubercle absent present;

{350 Coracoid_tubercle_form anteroposteriorly_short,_mound-like anteroposteriorly_elongated,_ridge-like;

{351 Coracoid_foramen present absent;

{352 Scapula_shape short_and_broad_(ratio_length/minimal_height_of_shaft_<9) slender_and_elongate_(ratio_>10);

{353 Scapulocoracoid_junction_anterior_surface indented_or_notched_between_the_scapular_acromial_process_and_the_coracoid_suture smoothly_curved_and_uninterrupted_across_the_contact_between_the_scapula_and_coracoid;

{354 Acromion_margin_of_scapula continuous_with_blade anterior_edge_enlarged_and_projects_anteriorly_at_approximately_a_right_angle;

{355 Flange_on__supraglenoid_buttress_on_scapula_(see_Nicholls_and_Russell,_1985)_ absent present;

{356 Distal_end_of_scapula expanded not_expanded;

{357 Glenoid_fossa_ faces_posteriorly_or_posterolaterally faces_laterally;

{358 Scapula_and_coracoid_ separate fused_into_scapulocoracoid;

{359 Scapula_and_coracoid_orientation continuous_arc_in_posterior_and_anterior_views coracoid_inflected_medially,_scapulocoracoid_L_shaped_in_lateral_view;

{360 Scapula_length longer_than_humerus shorter_than_humerus;

{361 Deltopectoral_crest_length less_than_one_quarter_humeral_length approximately_one_third_humeral_length greater_than_one_half_humeral_length;

{362 Deltopectoral_crest large_and_distinct,_proximal_end_of_humerus_quadrangular_in_anterior_view less_pronounced,_forming_an_arc_rather_than_being_quadrangular very_weakly_developed,_proximal_end_of_humerus_with_rounded_edges extremely_long_(as_in_Shuvuuia_and_Mononykus);

{363 Deltopectoral_crest_orientation longitudinal oblique_distolaterally_and_distal_end_of_crest_oriented_laterally_rather_than_anteriorly_from_the_humeral_shaft;

{364 Lateral_surface_of_distal_end_of_deltopectoral_crest smooth with_distinct_muscle_scar_near_lateral_edge_along_distal_end_of_crest_for_insertion_of_biceps_muscle;

{365 Ratio_femur/humerus more_than_2.5 between_1.2_and_2.2 less_than_1;

{366 Outline_of_proximal_articular_facet_of_humerus broadly_oval_(more_than_twice_as_broad_transversely_than_anteroposteriorly) distinctly_rounded,_often_globular_(less_than_twice_as_broad_anteroposteriorly_than_transversely);

{367 Internal_tuberosity_of_humerus small_and_confluent_with_humeral_head offset_from_humeral_head_by_distinct_notch,_often_projects_proximally_above_humeral_head hypertrophied_but_not_distinct_from_humeral_head_(as_in_Suchomimus);

{368 Shape_of_internal_tuberosity_on_humerus_in_anterior_view triangular,_often_rounded rectangular;

{369 Humerus_in_lateral_view sigmoidal straight;

{370 Transverse_width_of_distal_humerus greater_than_2.7_times_shaft_width between_2_and_2.5_times_humeral_shaft_width less_than_twice_shaft_width;

{371 Ectepicondyle_of_humerus_(lateral_epicondyle) small,_often_rectangular_and_does_not_form_articular_surface large,_rounded_and_forms_articular_surface;

{372 Entepicondyle_of_humerus_(medial_epicondyle) absent_or_small_and_tabular large,_projects_medially_from_ulnar_condyle_as_a_distinct_process_and_is_distally_separated_from_ulnar_condyle_by_a_groove;

{373 Distal_humeral_condyles primarily_developed_on_distal_end_of_humerus,_but_may_also_have_some_articular_surface_extending_to_anterior_edge limited_to_anterior_surface,_condylar_surfaces_not_present_on_distal_end;

{374 Ulnar_shaft straight bowed;

{375 Olecranon_process_of_ulna absent_or_weakly_developed well-developed hypertrophied;

{376 Shape_of_olecranon_process transversely_broad mediolaterally_thin,_blade-like;

{377 Crest_extending_along_posterior_surface_of_ulnar_shaft_from_olecranon_process absent present;

{378 Proximal_surface_of_ulna_ single_continuous_articular_facet divided_into_two_distinct_fossae;

{379 Proximal_end_of_the_ulna_in_proximal_view without_extensive_coronoid_process_and_radial_process_on_radial_side_of_proximal_end coronoid_and_medial_processes_large;

{380 Distal_articular_surface_of_ulna_ flat convex,_semilunate_surface;

{381 Distal_condyle_articular_surface_of_ulna unexpanded_or_spatulate,_articular_surface_limited_to_distal_end bulbous,_trochlear_articular_surface_extends_onto_dorsal_surface_of_ulna;

{382 Radius_length more_than_half_the_length_of_humerus less_than_half_the_length_of_humerus;

{383 Radial_shaft straight bowed_laterally;

{384 Radius_and_ulna well-separated with_distinct_adherence_or_syndesmosis_distally;

{385 Ossified_carpals absent present;

{386 Lateral_proximal_carpal_(ulnare?)_ quadrangular triangular_in_proximal_view;

{387 Trochlea_on_the_proximal_surface_of_distal_carpal_1 absent present;

{388 Two_distal_carpals_ in_contact_with_metacarpals,_one_covering_the_base_of_Mc_I_(and_perhaps_contacting_Mc_II)_,_the_other_covering_the_base_of_Mc_II two_distal_carpals_not_present,_single_distal_carpal_capping_Mc_I_and_II;

{389 Distal_carpals_ not_fused_to_metacarpals fused_to_metacarpals,_forming_carpometacarpus;

{390 Rectangular_buttress_on_ventrolateral_surface_of_proximal_end_of_Mc_I absent present;

{391 Length_of_Mc_I approximately_half_the_length_of_Mc_II subequal_in_length_to_Mc_II;

{392 Shape_of_Mc_I significantly_longer_than_broad very_stout,_approximately_as_long_as_broad;

{393 Contact_between_Mc_I_and_Mc_II metacarpals_contact_each_other_at_their_bases_only Mc_I_closely_appressed_to_Mc_II,_at_least_the_proximal_half_of_McI_flattened;

{394 Medial_tab_on_proximal_end_of_Mc_I_('proximo-radial_process_of_Gishlick_and_Gauthier,2007) absent_or_poorly_developed well-developed,_extending_far_medially;

{395 Distal_end_of_Mc_I condyles_more_or_less_symmetrical condyles_strongly_asymmetrical,_the_medial_condyle_being_positioned_more_proximally_than_the_lateral;

{396 Distal_articular_end_of_metacarpal_I ginglymoid rounded_and_smooth;

{397 Medial_side_of_Mc_II expanded_proximally not_expanded;

{398 Distal_articular_end_of_McII ginglymoid without_ginglymus;

{399 Shaft_of_Mc_III subequal_in_width_to_Mc_II considerably_more_slender_than_Mc_II_(less_than_70%_of_the_width_of_Mc_II);

{400 Proximal_articular_end_of_Mc_III expanded_and_similar_in_width_to_Mc_I_and_II not_expanded,_very_slender_when_compared_to_Mc_I_and_II;

{401 Proximal_outline_of_Mc_III subrectangular triangular,_apex_dorsal;

{402 Shaft_of_Mc_III straight bowed_laterally;

{403 Extensor_pits_on_the_dorsal_surface_of_the_distal_end_of_metacarpals absent_or_poorly_developed deep,_well-developed;

{404 Number_of_manual_digits_with_one_or_more_phalanges five four three two;

{405 Number_of_metacarpals five four three;

{406 Paired_flexor_processes_on_proximal_ventral_surfaces_of_proximalmost_phalanges absent present;

{407 Flexor_surface_of_manual_phalanx_I-1 convex_or_flat concave,_'axial_furrow'_along_proximodistal_axis;

{408 Shaft_diameter_of_phalanx_I-1 less_than_shaft_diameter_of_radius greater_than_shaft_diameter_of_radius;

{409 Proximodistal_length_of_phalanx_I-1/length_of_Mc_I 1_or_less between_1_and_1.5 more_than_1.5;

{410 Penultimate_phalanx_of_the_second_finger shorter_than_first_phalanx longer_than_first_phalanx;

{411 Penultimate_phalanx_of_the_third_finger as_long_as,_or_shorter_than,_more_proximal_phalanges longer_than_each_of_the_more_proximal_phalanges longer_than_both_proximal_phalanges_taken_together;

{412 Length_of_third_manual_digit longer_than_second_finger shorter_than_or_equal_in_length_to_second_finger;

{413 Proximal_articular_surface_of_manual_ungual_I-2 dorsoventrally_much_taller_than_mediolaterally_wide mediolaterally_as_broad_as_tall;

{414 Unguals_on_all_manual_digits generally_similar_in_size digit_I_bearing_large_ungual_and_unguals_of_other_digits_distinctly_smaller;

{415 Transverse_ridge_immediately_dorsal_to_the_articulating_surface_of_unguals absent present;

{416 Flexor_tubercle_placement proximal distal absent;

{417 Curvature_of_ventral_surface_manual_ungual_I strongly_curved weakly_curved straight;

{418 Curvature_of_ventral_surface_of_manual_unguals_II_and_III strongly_curved weakly_curved_ straight;

{419 Flexor_tubercle_size large_(>_1/3_articular_facet_height) small_(<_1/3_articular_facet_height);

{420 Lateral_grooves_of_manual_ungual_I-2_in_ventral_view unenclosed proximal_end_of_grooves_partially_enclosed_by_lateral_notches proximal_end_of_grooves_passes_through_foramena_on_ventral_surface_of_ungual;

{421 Fusion_of_pelvic_elements_in_adults absent present;

{422 Ilium brachyiliac dolichoiliac;

{423 Ilium_pneumaticity little_or_none large_external_pneumatic_foramina_and_internal_spaces;

{424 Dorsal_margin_of_ilium subhorizontal_or_gently_inclined_relative_to_axis_of_pubic_and_ischial_contact rises_steeply_as_it_extends_anteriorly,_at_least_30_degree_angle_from_the_axis_of_the_pubic_and_ischial_contact;

{425 Ventral_edge_of_anterior_ala_of_ilium_ straight_or_gently_curved ventral_edge_hooked_anteriorly;

{426 Form_of_hook_of_preacetabular_ala_of_ilium weak strong;

{427 Preacetabular_part_of_ilium significantly_shorter_than_postacetabular_part subequal_in_length_to_postacetabular_part significantly_longer_than_postacetabular_process;

{428 Anterior_rim_of_ilium shallowly_convex_or_straight strongly_convex_or_pointed_anteriorly;

{429 Dorsally-positioned,_anteriorly-concave_notch_on_anterior_rim_of_ilium absent present;

{430 Preacetabular_part_of_ilium_(height) approximately_as_high_as_postacetabular_part_(excluding_the_ventral_expansion) significantly_higher_than_postacetabular_part significantly_lower_than_the_postacetabular_part;

{431 Cuppedicus_fossa absent present;

{432 Form_of_cuppedicus_fossa deep,_ventrally_concave fossa_shallow_or_flat,_with_no_lateral_overhang;

{433 Cuppedicus_fossa_position ridge_bounding_fossa_terminates_rostral_to_acetabulum_or_curves_ventrally_onto_anterior_end_of_pubic_peduncle rim_extends_far_posteriorly_and_is_confluent_or_almost_confluent_with_acetabular_rim;

{434 Preacetabular_portion_of_ilium parasagittal moderately_laterally_flaring;

{435 Brevis_fossa_shape shelf-like,_narrow_with_subparallel_margins deeply_concave,_expanded_posteriorly_with_lateral_overhang;

{436 Brevis_fossa_lateral_view Poorly_developed_adjacent_to_ischial_peduncle,_without_lateral_overhang_and_medial_edge_of_the_brevis_fossa_is_visible well_developed_fossa_along_full_length_of_postacetabular_blade,_lateral_overhang_extends_along_full_length_of_fossa,_medial_edge_of_brevis_fossa_covered_in_lateral_view;

{437 Medial_brevis_shelf strongly_developed,_projects_medially low_ridge_on_medial_surface_of_postacetabular_ala;

{438 Shape_of_postacetabular_ala_of_ilium_in_lateral_view squared acuminate;

{439 Postacetabular_ala_of_ilium_in_lateral_view ventral_edge_flat ventral_edge_concave ventral_edge_concave_and_distal_end_extends_ventrally_below_level_of_the_ventral_margin_of_the_ischial_peduncle;

{440 Articulation_of_iliac_blades_with_sacrum vertical,_well-separated_above_sacrum strongly_inclined_mediodorsally,_almost_contacting_each_other_or__sacral_neural_spines_at_midline;

{441 Vertical_ridge_on_iliac_blade_above_acetabulum absent low_ridge_with_associated_foramina well-developed;

{442 Shape_of_pubic_peduncle_of_ilium transversely_broad_and_roughly_triangular_in_outline anteroposteriorly_elongated_and_narrow;

{443 Iliac_pubic_peduncle_length_relative_to_iliac_ischial_peduncle significantly_longer_than_ischial_peduncle,_ischial_peduncle_tapering_ventrally_and_without_clearly_defined_articular_facet subequal_in_length_to_ischial_peduncle anteroposteriorly_shorter_than_the_ischial_peduncle;

{444 Articulation_facet_of_pubic_peduncle_of_ilium facing_more_ventrally_than_anteriorly,_and_without_a_pronounced_kink with_pronounced_kink_and_anterior_part_facing_almost_entirely_anteriorly;

{445 Anterior_margin_of_pubic_peduncle straight_or_convex concave;

{446 Supraacetabular_crest absent_ present_;

{447 Form_of_supraacetabular_crest forms_hood_over_femoral_head reduced,_not_forming_hood;

{448 Antitrochanter_posterior_to_acetabulum_ absent_or_poorly_developed prominent;

{449 Postacetabular_blades_of_ilia_in_dorsal_view_ parallel diverge_posteriorly;

{450 Tuber_along_dorsal_edge_of_ilium,_dorsal_or_slightly_posterior_to_acetabulum_ absent present;

{451 Dorsal_margin_of_postacetabular_ala_in_lateral_view convex_or_straight concave,_brevis_shelf_extends_caudal_to_lateral_ilium_making_it_appear_concave_in_lateral_view;

{452 Caudal_end_of_postacetabular_ala_in_dorsal_view rounded_or_squared_in_dorsal_view lobate,_with_brevis_shelf_extending_caudally_beyond_caudal_terminus_of_the_postacetabular_ala;

{453 Ilium_and_ischium_articulation flat_or_slightly_concavo-convex with_process_projecting_into_socket_in_ischium;

{454 Pubic_orientation propubic vertical moderately_posteriorly_oriented opisthopubic;

{455 Strongly_expanded_pubic_boot absent present;

{456 Pubic_boot_projects anteriorly_and_posteriorly with_little_or_no_anterior_process only_expanded_anteriorly;

{457 Ratio_length_of_pubic_boot_to_length_of_pubic_shaft less_than_0.3 more_than_0.5;

{458 Pubic_boot_outline,_distal_view triangular narrow,_with_subparallel_margins;

{459 Pubic_apron present absent;

{460 Form_of_pubic_apron extends_medially_from_middle_of_cylindrical_pubic_shaft shelf_extends_medially_from_anterior_edge_of_anteroposteriorly_flattened_shaft;

{461 Pubic_apron about_half_of_pubic_shaft_length less_than_1/3_of_shaft_length;

{462 Pubic_apron completely_closed with_medial_opening_distally_above_the_pubic_boot;

{463 Pubic_obturator_foramen present absent;

{464 Form_of_pubic_obturator_foramen completely_enclosed open_ventrally_(obturator_notch);

{465 Pubic_fenestra_below_obturator_foramen absent present;

{466 Pubic_shafts_in_lateral_view straight anteriorly_convex anteriorly_concave;

{467 Lateral_face_of_pubic_shafts smooth with_prominent_lateral_tubercle_about_halfway_down_the_shaft;

{468 Length of 'Ischium^n^n''' more_than_two-thirds_pubis_length two_thirds_or_less_of_pubic_length;

{469 Obturator_process_of_ischium_ absent present;

{470 Position_of_obturator_process proximal_in_position located_near_middle_of_ischiadic_shaft located_at_distal_end_of_ischium;

{471 Ischial_shaft Rodlike anteroposteriorly_wide_and_plate_like;

{472 Lateral_blade_of_ischium flat_or_laterally_convex laterally_concave with_longitudinal_ridge_subdividing_lateral_surface_into_anterior_(including_obturator_process)_and_posterior_parts;

{473 Ischium,_lateral_view straight distally_curved_anteriorly distally_curved_posteriorly;

{474 Ischium,_anterior_view straight laterally_convex twisted_at_midshaft_and_with_flexure_of_obturator_process_toward_midline_so_that_distal_end_is_horizontal laterally_concave;

{475 Contact_of_obturator_process_of_ischium does_not_contact_pubis contacts_pubis;

{476 Ventral_notch_at_distal_edge_of_ischial_obturator_process_ absent,_grades_smoothly_into_ischial_shafts present;

{477 Obturator_process_on_ischium confluent_with_pubic_peduncle offset_from_pubic_peduncle_by_a_distinct_notch;

{478 Morphology_of_offset_triangular_obturator_process_of_ischium wide_base_along_ischiac_shaft,_rostral_process_short narrow_base,_rostral_process_elongate;

{479 Distal_end_of_ischium strongly_expanded,_forming_ischial_"boot" slightly_expanded tapering;

{480 Distal_ends_of_ischia_ form_symphysis approach_one_another_but_do_not_form_symphysis widely_separated;

{481 Distally_placed_process_on_caudal_margin_of_ischium absent present;

{482 Tubercle_on_anterior_edge_of_ischium absent present;

{483 Posterior_process_(ischial_tuberosity)_on_posteroproximal_part_of_ischium absent_ well-developed;

{484 Form_of_posteroproximal_ischial_process_(ischial_tuberosity) small,_tablike large,_proximodorsally_hooked_and_separated_from_the_iliac_peduncle_by_a_notch;

{485 Semicircular_scar_on_posterior_part_of_the_proximal_end_of_the_ischium absent present;

{486 Femoral_length longer_than_tibia shorter_than_tibia;

{487 Femoral_head without_fovea_capitalis circular_fovea_present_in_center_of_medial_surface_of_head;

{488 Oblique_ligament_groove_on_the_posterior_surface_of_femoral_head absent_or_very_shallow deep,_bound_medially_by_a_well-developed_posterior_lip;

{489 Femoral_head_and_greater_trochanter confluent_with_greater_trochanter separated_from_greater_trochanter__by_a_distinct_cleft;

{490 Femoral_head_direction_anteroposterior directed_anteromedially directed_strictly_medially;

{491 Femoral_head_direction_dorsoventral ventromedial horizontal dorsomedial;

{492 Greater_trochanter anteroposteriorly_narrow_and_narrowing_from_medial_to_lateral anteroposteriorly_expanded,_forming_a_trochanteric_crest;

{493 Lesser_trochanter separated_from_greater_trochanter_by_a_deep_cleft trochanters_separated_by_small_groove completely_fused_(or_absent)_to_form_crista_trochanteris;

{494 Lesser_trochanter_shape alariform cylindrical_in_cross_section very_short_and_ridge-like;

{495 Proximal extent of lesser 'trochanter^n^n''' at_distal_end_of_femoral_head more_proximally_placed,_but_distal_to_greater_trochanter as_proximal_or_more_proximal_than_greater_trochanter;

{496 Accessory_trochanteric_crest_on_distal_end_of_lesser_trochanter absent present;

{497 Posterolateral_trochanter absent_or_represented_only_by_rugose_area posterior_trochanter_distinctly_raised_from_shaft,_mound-like;

{498 Fourth_trochanter_on_femur_ present absent;

{499 Broad_groove_on_extensor_surface_of_distal_femur absent_or_poorly_developed well_developed;

{500 Femoral_medial_epicondyle_(medial_distal_crest,_expanded_medial_lamella) stout_ridge_or_absent flange_like,_medially_extensive;

{501 Popliteal_fossa_on_distal_end_of_femur open_distally closed_off_distally_by_contact_between_distal_condyles;

{502 Infrapopliteal_ridge_present_posteriorly_between_medial_condyle_and_crista_tibiofibularis absent present;

{503 Distal_end_of_femur anteroposteriorly_broad_and_distally_flattened less_broad_and_well_rounded;

{504 Lateral_femoral_distal_condyle distally_rounded distally_conical;

{505 Distal_projection_of_lateral_femoral_distal_condyle approximately_the_same_level_as_the_medial_condyle distinctly_further_than_medial_condyle_and_distal_surface_of_medial_condyle_is_flattened;

{506 Anteroposterior_length_of_proximal_end_of_tibia_in_proximal_view exceeds_mediolateral_width less_than_mediolateral_width;

{507 Cnemial_crest_proximal_projection approximately_at_the_same_level_as_posterior_condyles projects_strongly_proximal_to_posterior_condyles;

{508 Anteroposterior_length_of_cnemial_crest prominent_but_not_expanded anteroposteriorly_expanded;

{509 Accessory_ridge_on_lateral_surface_cnemial_crest absent present;

{510 Medial__cnemial_crest_and_lateral_cnemial_crest(also_called_the_cranial_cnemial_crest_in_birds) absent present;

{511 Fibular_condyle_on_proximal_end_of_tibia confluent_with_cnemial_crest_anteriorly_in_proximal_view strongly_offset_from_cnemial_crest;

{512 Medial_proximal_condyle_on_tibia round_in_proximal_view arcuate_and_posteriorly_angular_in_proximal_view;

{513 Posterior_cleft_between_medial_part_of_the_proximal_end_of_the_tibia_and_fibular_condyle absent present;

{514 Fibular_crest_(ridge_on_lateral_side_of_tibia_for_connection_with_fibula) absent present;

{515 Form_of_fibular_crest extending_from_proximal_articular_surface_distally clearly_separated_from_proximal_articular_surface;

{516 Shape_of_fibular_crest quadrangular low_and_rounded_;

{517 Fibular_crest_distal_extension proximally_positioned extends_to_midshaft_of_tibia;

{518 Fibular_crest_length short,_less_than_one_fifth_tibial_length long,_between_one_quarter_and_one_third_tibial_length;

{519 Bracing_for_ascending_process_of_astragalus_on_anterior_side_of_distal_tibia distinct_'step'_running_obliquely_from_mediodistal_to_lateroproximal anterior_side_of_tibia_flat Step-like_ridge_running_proximodistally_rather_than_obliquely;

{520 Fibula reaches_proximal_tarsals short,_tapering_distally,_and_not_in_contact_with_proximal_tarsals_;

{521 Lateral_surface_of_proximal_fibula shallow_longitudinal_trough_situated_posteriorly trough_absent_or_weak_groove_present,_surface_convex;

{522 Proximal_fibular_margin subhorizontal cranial_portion_extends_proximally_beyond_level_of_posterior_portion;

{523 Fibular_proximal_dimensions_in_proximal_view anterior_portion_subequal_to_posterior_portion_in_mediolateral_width anterior_portion_mediolaterally_wider_than_posterior_portion;

{524 Insertion_of_m._iliofibularis_on_fibular_shaft not_especially_marked present_as_a_well-developed_anterolateral_tubercle;

{525 Position_of_insertion_of_m._iliofibularis_on_fibular_shaft proximal midshaft;

{526 Ridge_on_medial_side_of_proximal_end_of_fibula,_that_runs_anterodistally_from_the_posterproximal_end absent present;

{527 Medial_surface_of_proximal_end_of_fibula concave_along_long_axis flat;

{528 Deep_oval_fossa_on_medial_surface_of_fibula_near_proximal_end absent present;

{529 Astragalus_and_Calcaneum condyles_indistinct_or_poorly_separated distinct_condyles_separated_by_prominent_vertical_tendinal_groove_on_anterior_surface;

{530 Astragalus_and_calcaneum separate_from_tibia fused_to_each_other_and_to_the_tibia_in_late_ontogeny;

{531 Fibular_facet_on_astragalus large_and_facing_partially_proximally reduced_and_facing_laterally_or_absent;

{532 Height_of_ascending_process_of_the_astragalus lower_than_astragalar_body higher_than_astragalar_body more_than_twice_the_height_of_astragalar_body;

{533 Shape_of_ascending_process_of_the_astragalus broad,_covering_most_of_anterior_surface_of_distal_end_of_tibia narrow,_covering_only_lateral_half_of_anterior_surface_of_tibia;

{534 Notch_on_medial_edge_of_ascending_process_of_the_astragalus absent present;

{535 Fossa_on_anterior_surface_of_mesial_base_of_ascending_process_of_astragalus,_sometimes_bearing_accessory_fenestrations absent present;

{536 Ascending_process_of_astragalus_and_astragalar_body confluent_or_only_slightly_offset_from_astragalar_body offset_from_astragalar_body_by_a_pronounced_groove;

{537 Astragalar_condyles almost_entirely_below_tibia_and_face_distally significantly_expanded_proximally_on_anterior_side_of_tibia_and_face_anterodistally;

{538 Horizontal_groove_across_astragalar_condyles_anteriorly absent present;

{539 Calcaneum without_facet_for_tibia well-developed_facet_for_tibia_present;

{540 Distal_tarsals separate,_not_fused_to_metatarsals form_metatarsal_cap_with_intercondylar_prominence_that_fuses_to_metatarsal_early_in_postnatal_ontogeny;

{541 Metatarsals_coossification not_co-ossified coossified;

{542 Shafts_of_metatarsals_II-IV not_closely_appressed_beyond_proximal_half_of_metatarsus closely_appressed_throughout_most_of_metatarsus,_adjacent_surfaces_flattened_for_contact;

{543 Maximum_length_of_metatarsals greater_than_50%_tibia_length less_than_50%_tibia_length;

{544 Metatarsal_I present absent;

{545 Metatarsal_I attenuates_proximally_,without_proximal_articulating_surface proximal_end_of_Mt_I_similar_to_that_of_Mt_II-IV;

{546 Metatarsal_I contacts_the_ankle_joint does_not_contact_the_ankle_joint;

{547 Position_of_distally-placed_Mt_I reduced,_elongated_and_splint-like,_articulates_in_the_middle_of_the_medial_surface_of_Mt_II broadly_triangular_and_attached_to_the_distal_quarter_of_Mt_II;

{548 Metatarsal_II_proximal_end_of_flexor_surface flat_or_small_tab_present large_quadrangular_flange_present;

{549 Distal_end_of_metatarsal_II smooth,_not_ginglymoid with_developed_ginglymus;

{550 Tuber_along_extensor_surface_of_MtII absent present;

{551 Posteromedial_margin_MtII_diaphysis well-developed_flange_absent_or_area_rugose with_flange_projecting_caudally_or_medially;

{552 Distal_end_of_metatarsal_III smooth,_not_ginglymoid with_developed_ginglymus;

{553 Metatarsal_III subequal_in_width_to_Mt_II_and_IV_proximally pinched_between_II_and_IV_and_not_visible_in_anterior_view_proximally does_not_reach_the_proximal_end_of_the_metatarsus mediolaterally_much_wider_than_either_II_or_IV;

{554 Metatarsal_III_shape_of_proximal_end rectangular,_medial_and_lateral_surfaces_pinched hourglass-shaped,_medial_and_or_lateral_surface(s)_concave;

{555 Medial_side_of_anterior_surface_of_distal_end_of_MtIII unexpanded expanded;

{556 Metatarsal_III_shape_of_shaft_in_cross_section rectangular wedge-shaped,_plantar_surface_pinched;

{557 Shaft_of_MT_IV round_or_thicker_dorsoventrally_than_wide_in_cross_section shaft_of_Mt_IV_mediolaterally_widened_and_flat_in_cross_section;

{558 Length_of_MtIV subequal_to_Mt_II markedly_longer_than_Mt_II;

{559 Posterolateral_margin_of_MtIV_diaphysis well-developed_flange_absent_or_area_rugose with_flange_projecting_caudally_or_laterally;

{560 Metatarsal_V with_rounded_distal_articular_facet strongly_reduced_and_lacking_distal_articular_facet short,_without_articular_surface,_transversely_flattened_and_bowed_anteriorly_distally;

{561 Pedal_digit_IV significantly_shorter_than_III_and_subequal_in_length_to_II,_foot_is_symmetrical significantly_longer_than_II_and_only_slightly_shorter_than_III,_foot_is_asymmetrical;

{562 Extensor_ligament_pits_on_dorsal_surface_of_phalanges_of_pedal_digit_IV shallow,_extensor_ridges_not_sharp deep_and_extensive_proximally,_corresponding_extensor_ridges_sharply_defined_in_dorsal_view;

{563 Pedal_phalanges_of_digit_IV anteroposteriorly_short,_with_proximal_and_distal_articular_surfaces_very_close_together,_particularly_in_distal_elements anteroposteriorly_long,_proximal_and_distal_articular_surfaces_well-separated;

{564 Shape_of_ventral_surface_of_pedal_unguals ventrally_concave_in_lateral_view straight_in_lateral_view;

{565 Ungual_and_penultimate_phalanx_of_pedal_digit_II similar_to_those_of_III highly_modified_for_extreme_hyperextension,_ungual_more_strongly_curved_and_about_50%_larger_than_that_of_III;

{566 Ventral_surface_of_pedal_unguals without_a_flexor_fossa,_ventral_surface_of_proximal_end_convex with_a_pronounced_flexor_fossa_on_ventral_surface_of_proximal_end;

{567 Form_of_flexor_fossa_on_pedal_unguals without_development_of_flexor_tubercle small_flexor_tubercle_present_within_flexor_fossa;

;

ccode + 73 81 99 118 124 131 149 178 182 218 225 226 232 235 236 262 263 271 272 277 281 301 302 310 318 321 323 324 337 346 361 365 367 370 375 404 405 409 411 416 418 420 441 443 454 478 480 493 495 532 553 560 *;

proc /;

comments 160

{0 436 It's uncertain whether herrarasaurus has a brevis fossa, however, a distinct muscle attachment "furrow" is visible in the same position, therefore I regard it as homologous;

{1 153 Tentative scoring;

{1 391 Although the first metacarpal is still much shorter than the second metacarpal, the ratio of length I to II is more than 0.5, scored this as 0 to homologize the condition with Harpymimus;

{1 562 Need to get Billy to confirm this scoring;

{2 69 Although three foramena exist in this region (Eddy, 2008), only the largest appears to be a true lacrimal pneumatic recess.;

{2 512 this character is the same as Benson, 2009 #201;

{5 81 Here the prefrontal only contributes slightly to the orbital margin so I've scored it a 1, double check this at the USNM;

{5 159 Benson, 2009 codes them as narrowly separated, but they are clearly outside the lateral condylar margins in Madsen, 1979;

{5 213 Benson, 2009 codes it as open, but it appears that the element may be damaged and the foramen is fully enclosed;

{5 250 Rauhut and Kirkland et al disagree here - Rauhut considers the neural spine of the axis to be cylindrical and mediolaterally compressed. Based on Madsen 1976, I agree with Kirkland et al that the neural spine is transversely flared.;

{5 279 Benson, 2009 codes this as weak or absent but I disagree;

{5 372 the entepicondyle of Allosaurus is very weakly developed, contrary to Benson, 2009;

{5 435 Here Rauhut and Kirkland et al disagree. Further research;

{6 255 Here I disagree with Novas over whether Alvarezsaurus has a cervical centrum with pleurocoels - the cervical centrum i found in the collection doesn't have them, but isn't very similar to what Novas 1996 figures.;

{6 276 A hypapophysis does appear to be present, if weakly developed, on the isolated centrum. Not sure of its position in the cervical series, but it appears to be anterior.;

{6 301 This is speculative, but based on the divergence of the postacetabular ala and the position of the other sacral vertebrae, Alvarezsaurus definitely had at least four, likely five, and probably not six.;

{6 303 There is a keel on the last preserved vertebrae, but it isn't as well-developed as in Shuvuuia. The middle sacrals are ventrally grooved and the anterior sacral is convex ventrally.;

{6 306 It's possible here that Bonaparte got the pelvis backwards on the initial description - double check this. Should really be marked as a question mark.;

{6 314 This coding taken from Chiappe, but the holotype MUCPv 54 no longer includes this material. There are two partially articulated vertebrae that seem to match the description, though, and they are ventrally keeled, although they don't have the ridiculous condition that is present in Shuvuuia. ;

{6 435 Looking at the holotype MUCPv 54 clearly shows that the brevis fossa is mediolaterally broad. This character should be revised, it's unclear what is meant by lateral overhang.;

{6 497 There is a distinct low swelling on both proximal femora of the holotype MUCPv 54;

{6 498 The slightest suggestion of a low ridge is present in this position on the right femur of the holotype, and it is even weaker on the left femur of the holotype.;

{6 519 Proximal to the flattened surface for articulation with the ascending process, there is what appears to be a medially positioned, short grooved extending proximally. Breakage of the specimen in this region, however, makes it hard to determine whether this feature is real. It would be an interesting homology with Farragochela. ;

{6 529 The groove is obliquely oriented, but is present;

{6 561 This is impossible to tell - Bonaparte's reconstruction infers a lot about phalanges that really can't be figured out without a complete preparation of the specimen.;

{7 183 HERE THERIZINOSAUROIDS ONLY HAVE THE CONVEXITY BECAUSE OF THE SYMPHYSEAL DOWNTURN, THAT IS THERE'S NO DORSAL EMINENCE SO THEY GET A 0;

{10 63 A small posterior process is present;

{15 19 This is labeled ifc on the Makovicky et al 2003 paper. Should check it at the AMNH;

{16 75 Although there is a posterodorsal process, witmer et al 2007 identify this as a fused prefrontal, a likely scenario given the rugose skull;

{17 392 Here Rauhut and Kirkland et al disagree. Do further research;

{17 477 Rauhut and Kirkland et al disagree here. Further research required;

{17 494 Here, Rauhut and Kirkland disagree again on the shape of the lesser trochanter. Further research necessary;

{17 553 Here, rauhut and Kirkland et al disagree. Research further;

{18 149 Benson codes them as straight, but Madsen and Welles show them as ventrolaterally projecting;

{20 85 The postorbital forms the majority of the orbital margin, unlike most other theropods, but there is a clear groove on the lateral surface of the frontal that articulates with the frontal process of the postorbital;

{21 247 These are figured in Colbert, 1989, Fig 46B p71;

{22 29 Benson, 2009 incorrectly codes this as pointed;

{22 85 Scored from Makovicky, personal communication in reference to Makovicky and Turner 2008;

{22 99 scored from Peyer 2006;

{22 265 Peyer 2006 says that compsognathus has opisthocoelus cervical centra, but this really isn't true, especially compared to the markedly opisthocoelus centra of the alvarezsaurids;

{22 302 Peyer 2006: there may be a small pleurocoel close to sutural boundary with neural arch;

{22 354 Here, Rauhut and Kirkland et al disagree. Further research necessary;

{22 476 Benson codes this as present but Peyer 2006 figures it as absent;

{25 69 Smith et al 2007 don't consider this a lacrimal foramen, but it is developed in the same place as that of eustrptospondylus.;

{26 214 I'm questioning what exactly constitutes "forked";

{28 6 Angle appears greater than 75 degrees, but body is longer in front of naris;

{28 8 Difficult to determine from article;

{28 265 Make sure to check this character state, as Peyer considered it synapomorphic of the Alvarezsaurs;

{29 476 Notch is present figured by Welles, 1984 not absent as in Benson, 2009;

{34 13 This area is damaged on the right side, but the left side doesn't reveal any evidence of a snf;

{35 15 It appears that a maxillary fenestra is present on the right maxilla of the mounted specimen.;

{36 34 Zanno et al, 2009 has the maxilla labeled backwards, so that the medial labels appear on the lateral picture and vice versa;

{42 13 Benson, 2009 considers it absent, but it is present on the paratype;

{42 44 Benson, 2009 codes this character as unfused for Guanlong, but the dorsal surface is fused in the larger skull, with a ventral suture visible. A similar situation is present in Eotyrannus, so I'm keeping it coded as fused.;

{42 119 There is a hole in the dorsal surface of the ectopterygoid, but I interpret this as a preservational artifact.;

{42 144 This really isn't a basisphenoid depression at all, as it's contained within the subcondylar recess which is clearly formed by the union of the basioccipital and the exoccipital/opisthotic;

{42 193 Though there is a small groove on the labial surface of the left dentary in the smaller specimen, it is very shallow.;

{42 206 retroarticular process is broken in the smaller specimen;

{42 228 The anterior two maxillary teeth have both anterior and posterior carinae, but the more posterior teeth are missing the mesial (anterior) carina, as in Proceratosaurus;

{42 291 The anterior dorsals have separated hyposphenes, while the posterior dorsals have hyposphenes that meet to form a lamina.;

{42 294 On the posterior surface of the neural spine, they terminate close to the apex, and on the anterior surface, well below the apex.;

{42 435 The brevis shelf is unusually wide at the distal end and funnel shaped, though there isn't any lateral expansion as in ornithomimosauria;

{42 496 The lesser trochanter has a novel shape to it, consisting of two bulges, one located more proximally and one more distally;

{43 26 Must check on this state for Shuvuuia, as the maxilla does have an offset, although it is dorsally and posteriorly displaced;

{43 68 Check this on the CT data;

{43 298 here, the posteriormost parapophyses are just a small bit more ventrally located than the diapophyseal facets;

{43 299 very tentative scoring - I don't know this character very well;

{43 406 Haplocheirus has a single ventral proximal tubercle that is similar to the condition present in Falcarius, where the tubercle is offset.;

{43 476 Unfortunately, this notch is very different in form from the notch in Allosaurus and it's probably not homologous. Refine the states;

{44 262 The cervical vertebrae have been greatly compressed mediolaterally during preservation, but they are very long, and probably in the area of 3-5 times as long as wide.;

{44 270 Cervicals only presented in lateral view, discussion is limited and they are badly crushed;

{44 387 The distal carpal bears a shallow trochlea proximally;

{47 13 I tentatively score this character as present, as there is a small foramen on the maxillary posterior process of the premaxilla.;

{49 496 Turner et al, 2007 label this as lr? in their Figure 2;

{50 200 A foramen is present,but it is in a very anterior position, which we tentatively homologize with the more posteriorly positioned surangular foramina of other theropods;

{52 20 Carrano, Sampson and Forster only mention the possibility of the homologous condition to a true promaxillary fenestra.;

{52 237 This character diagnostic of Masiakasaurus - procumbent anterior maxillary teeth with carinae that have been rotated 90 degrees out of their usual plane;

{52 238 Anterior teeth are spatulate, posterior teeth are mediolaterally flattened and recurved;

{52 487 Take a look at the proximal sulcus with Jim. It appears that this sulcus is a fovea capitalis.;

{52 496 Is this the same as the trochanteric shelf of Hutchinson, 2001?;

{52 513 Fibular condyle of Rauhut = lateral condyle of other others;

{56 250 Again, Rauhut and Kirkland et al disagree. Further study needed;

{56 494 Kirkland and Rauhut disagree again;

{57 444 Benson codes this as facing anteriorly, but I disagree;

{59 13 Benson, 2009 considers it absent, but I saw it on the holotype;

{59 512 Benson codes this as bulbous, but it's arcuate in my opinion;

{61 85 Suggestion of a groove is present on both sides of the skull but scoring is provisional due to damage;

{61 294 Or so say Carpenter, Miles, Ostrom and Cloward, 2005;

{61 435 Here Rauhut and Kirkland disagree;

{61 479 Here Rauhut and Kirkland et al disagree. More research needed. (Looking at the AMNH #619 specimen shows almost no distal expansion of the ischium, hence state 0)

;

{64 511 The incisurua tibialis is very shallow, as in Eustreptospondylus;

{65 277 Chiappe et al 2003 say that the dorsal pleurocoels are absent in all alvarezsaurids;

{65 349 There is no coracoid tuber in Patagonykus, but like Haplocheirus, the posteroventral blade features a triangular low ridge on the lateral surface that extends for the length of the blade and terminates just posteroventral to the coracoid foramen. This is not well-developed in Alvarezsaurus, but little of the coracoid is preserved in that taxon. Makovicky calls this the subglenoid fossa, and it is well-developed in ornithomimosaurs and also in Nqwebasaurus.;

{65 359 Turner has this one wrong, the coracoid is clearly inflected medially in Novas 1997, figure 145;

{65 389 There is no evidence for fusion of the distal carpals into the carpometacarpus.;

{65 393 There is a clear surface on the proximal half of the lateral surface of McI for articulation with the second metacarpal. The lateral surface of the distal condyle also bears a small flattened area that suggests it contacted the distal portion of McII.;

{65 407 The ventral surface does bear an axial furrow, but the median portion of this furrow has a small, X-shaped area where two ridges cross each other and form a raised area with two lateral foramina. This is best developed in PVPH 102;

{65 416 Coded from PVPH 102;

{65 458 Novas got the identification wrong, it's actually the left pubis.;

{65 467 Not to be confused with the more proximally located pubic tubercle, which is the attachment for m. ambiens and m. rectus abdominis.;

{65 509 It appears from the photographs that the medial cnemial crest of the tibia in parvicursorines may actually pertain to the cnemial crest, and that the lateral of the two pertains to the laterally-developed cnemial crest because in Patagonykus the lateral cnemial crest is very proximal and looks like an incipient version of parvicursorines.;

{65 510 Patagonykus has an incipient lateral cnemial crest developed as a triangular tuber immediately anterior to the fibular condyle.;

{65 538 A slight suggestion of a groove is present on the right astragalar medial condyle, but I've elected to score it as absent because the contralateral side does not preserve any groove.;

{66 36 An incipient ridge is present, but it is very weakly developed;

{66 158 Although the basal tubera are clearly visible, they are not fused and I can't sort this character out.;

{66 178 The mandibular foramen looks large in the sense that it stretches from the posterior end of the surangular below the postorbital anteriorly to a point just ventral to the lacrimal.;

{66 187 The lower jaw of the holotype is close against the maxilla and there is a slight downward deflection of the anterior end so that there is a gap between the premaxilla and the dentary that isn't present between the maxilla and the dentary;

{66 202 The presence of this ridge is confirmed on the left side of the skull by a poorly developed lateral flange just dorsal to the surangular foramen and a corresponding lateral flange on the anterior end of the surangular near the dentary contact. In between, the ridge is covered by the jugal laterally.;

{66 218 There are six;

{66 230 Amazingly, the teeth resemble those of Falcarius closely, but they don't have serrations;

{66 270 Inspection of the right side of the skull block reveals that an extensive connection between the postzygapophyses was likely present;

{67 165 Rauhut 2004 figures a dorsal depression, but it isn't developed as a pneumatic recess as in the braincase of troodontids;

{68 65 The articular facet on the jugal is only slightly anterior to the infratemporal fenestra, but the anterior tip of the quadratojugal lies posterior to posterior margin;

{68 247 Although striations are present on some maxillary teeth (Rauhut et al, 2009), they do not resemble those of spinosaurids, being rounded and larger;

{73 447 A very small ridge-like crest extends from the posteroventral base of the pubic peduncle along the rim of the acetabulum, ending approximately 2/3 of the way to the ischial peduncle.;

{73 450 The tuber is present, but isn't as pronounced as in adasaurus mongoliensis. Tuber is directly above ischial peduncle.;

{73 524 Clearly defined tubercle that Rauhut illustrates well in Figure 48 (2003)

;

{73 531 The fibular facet is a small deep pocket that faces proximately and laterally and posteriorly. Here i code it as reduced.;

{73 539 It appears to me that the calcaneum and astragalus are fused. If this is true, the calcaneum merely forms the lateral edge of the fibular facet, without being excavated or emarginated, so it doesn't have a true facet.

;

{75 19 This scoring very tentative - an embayment is described in this region by Kobayashi et al, but no illustration is presented. The specimen is not adequately prepared to score this character.;

{75 270 Does not have cervicals;

{76 68 There does seem to be a slit-like foramen opening beneath the lateral lacrimal fold, but this is full of matrix in IGM 100/0977 and is not preserved adequately in IGM 100/1001;

{76 75 It is listed as present in Chiappe et al 2002;

{76 133 The dorsal recess on the basipterygoid makes them hollow, but this differs from the inflated condition present in derived troodontids, Chirostenotes and ornithomimosaurs;

{76 329 I'm pretty sure they're present on IGM 100/0977;

{78 19 Athough Xu and Wu reconstruct the skull without this foramen, the specimen appears to be damaged in this region, so is scored as uncertain;

{81 55 Benson, 2009 notes that there is a ventral process under the antorbital fossa, but I haven't seen this so I've kept it as state 0;

{81 141 Another instance where the basisphenoid is oriented almost at a 45 degree angle to the horizontal plane.;

{81 159 Benson codes them as narrowly separated, but they are clearly lateral to the occipital condyle;

{81 511 Benson, 2009 has this coded wrong;

{82 263 Crushing in the specimen and mediolateral deformation preclude the assessment of this character;

{82 434 Crushing in the specimen and mediolateral deformation preclude the assessment of this character;

{82 449 Crushing in the specimen and mediolateral deformation preclude the assessment of this character;

{82 464 Probably a foramen, there is broken bone surrounding the rim and the pubic apron is poorly developed that high up so it can't be a major contribution of the pubic apron.;

{82 473 The distal ends of both ischia abandon the straight trajectory of the shaft and curve ventrally.;

{82 485 The semicircular scar is a rugose patch in tyrannosaurus, since it gets a 'present' I give the mildly rugose patch in Stokesosaurus a 1;

{82 499 There is at least a suggestion of the presence of this groove on both sides.;

{85 141 Interestingly, the basisphenoid of syntarsus is mor vertically oreiented than in many coelurosaurs. Might have to change the states to account for this.;

{85 367 A small notch separates the internal tuberosity, which is located well distal to the humeral head.;

{86 354 Benson, 2009 codes this as a 0 but he is wrong;

{86 498 Benson codes it as absent, it's clearly present;

{89 379 Benson, 2009 codes this as absent, but from the Galton and Jensen, 1979 publication it is clearly present;

{90 111 MOR 116 from the Judith river is an isolated broken right quadrate and the ventral portion reveals a completely hollow quadrate. Also, a pneumatic foramen exists in the medial side of the quadrate.;

{90 117 MOR 116 and 170 clearly show a quadrate foramen opening between the quadrate and quadratojugal.;

{90 180 The interdental plates seem tightly fused to the dentary and there are individual foramina between the plates, but there does seem to be some plate development in MOR 563 and 553, particularly at the anterior end of the dentary.;

{92 17 Here, I've scored it as posterior because on the medial surface there is a large promaxillary recess rostral to the opening of the maxillary fenestra

;

{92 33 Must measure this;

{93 283 The most posterior neural spine is ever so slightly anteriorly oriented, but not the derived condition for sure;

{93 292 This expansion is slight but present;

{93 432 The anterior end of the fossa has a well-developed lateral overhang of the preacetabular hook, but the posterior end has no overhang and is laterally directed;

{93 447 State 0 edited by JNC;

{93 451 This posterior extension of the brevis shelf is a very subtle feature, but it is present;

{93 457 It's hard to tell because the distal end of the pubic boot still has matrix adhering to its surface and this the length of the boot may be exaggerated.;

{93 468 This really should be a question mark - the distal end of the ischium is broken.;

{93 488 A groove in this postion actually appears to be insect damage postmortem;

{93 495 No way to tell this on PVPH 78, the proximal end of the lesser trochanter is broken;

{93 497 A distinct ridge is present here, and adjacent is a large, hollowed out area but this appears to be damage to the specimen;

{96 20 While Clark does not consider the depression to be a fenestra promaxillaris, I do. Talk to Jim to get his opinion;

{96 220 Based on the size of the root, all premaxillary teeth are of equal size;

{96 265 Here the anterior articular surface is flat or very slightly rounded anteriorly, but the posterior articulation is deeply excavated.;

;
